# In silico deconjugation of glucuronide conjugates enhances tandem mass spectra library annotation of human samples

**DOI:** 10.1007/s00216-022-03899-7

**Published:** 2022-01-26

**Authors:** Carolin Huber, Martin Krauss, Vera Reinstadler, Sara Denicolò, Gert Mayer, Tobias Schulze, Werner Brack, Herbert Oberacher

**Affiliations:** 1 Department of Effect-Directed Analysis, Helmholtz Center for Environmental Research-UFZ, Permoserstraße 15, 04318 Leipzig, Germany; 2 Institute of Ecology, Diversity and Evolution, Goethe University Frankfurt Biologicum, Campus Riedberg, Max-von-Laue-Str. 13, 60438 Frankfurt am Main, Germany; 3 Institute of Legal Medicine and Core Facility Metabolomics, Medical University of Innsbruck, 6020 Innsbruck, Austria; 4 Department of Internal Medicine IV (Nephrology and Hypertension), Medical University of Innsbruck, 6020 Innsbruck, Austria

**Keywords:** Drug monitoring/drug screening, Glucuronide, Human biomonitoring, Mass spectrometry, Metabolites, Spectral library search

## Abstract

Mass spectral library annotation of liquid chromatography-high resolution tandem mass spectrometry (LC-HRMS/MS) data is a reliable approach for fast identification of organic contaminants and toxicants in complex environmental and biological matrices. While determining the exposure of humans or mammals, it is indispensable to include phase I and phase II metabolites (conjugates) along with the parent compounds, but often, tandem mass spectra for these are unavailable. In this study, we present and evaluate a strategy for annotating glucuronide conjugates in LC-HRMS/MS scans by applying a neutral loss search for detection, then truncating the spectra which we refer to as in silico deconjugation, and finally searching these against mass spectral libraries of the aglycones. The workflow was tested on a dataset of in vitro*–*generated glucuronides of reference standard mixtures and a dataset of 51 authentic urine samples collected from patients with known medication status, acquired on different instrumentations. A total number of 75 different glucuronidated molecular structures were identified by in silico deconjugation and spectral library annotation. We also identified specific molecular structures (sulfonamides, ether bonds, di-glucuronides), which resulted in slightly different fragmentation patterns between the glucuronide and the unconjugated compound. This led to a decreased spectral matching score and in some cases to a false-negative identification. Still, by applying this method, we revealed a reliable annotation of most common glucuronides, leading to a new strategy reducing the need for deconjugation steps or for recording many reference glucuronide spectra for screening approaches.

**Graphical Abstract:**

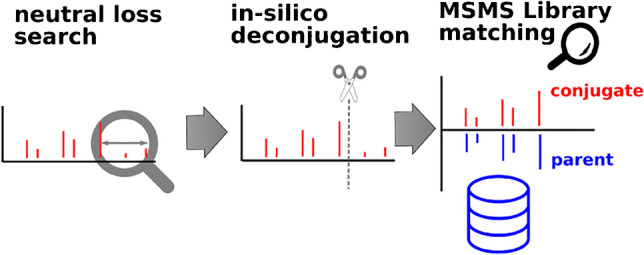

**Supplementary Information:**

The online version contains supplementary material available at 10.1007/s00216-022-03899-7.

## Introduction

Screening and non-targeted methods have increased in popularity and application for qualitative analysis of hundreds of compounds simultaneously [1]. With an increasing number of compounds being available in mass spectral libraries, the application of a database search of tandem mass (MS/MS) spectra in fast annotation workflows can be performed to identify compounds at a high confidence level (level 2A of the Schymanski scale [2]).

Metabolite detection is necessary for many mass spectrometry applications, including medical studies, pharmaceutical research, forensic science, doping control, metabolomics, human biomonitoring, wastewater-based epidemiology and others. Mammalian metabolites can be grouped in phase I metabolites, occurring mainly from oxidation reactions and phase II metabolites, formed by conjugation reactions of parent compounds or phase I metabolites. Glucuronidation is one major conjugation reaction in phase II metabolism [3]. Functional groups, like carboxyl, hydroxy, or primary and secondary amino groups, are known to get conjugated with glucuronic acid involving a family of uridine 5′-diphospho-glucuronosyl-transferase enzymes [4]. The conjugation of a xenobiotic compound increases its water solubility and therefore enhances urinary excretion [5]. Although glucuronidation is in general considered as a detoxifying process, some glucuronides, especially on acyl groups, seem to be responsible for the observed drug toxicity [6]. Based on the clearance mechanisms presented on RxList (drug index for prescription drug information, interactions and side effects), glucuronidation is listed as a relevant excretion pathway for approximately one in ten of the top 200 drugs prescribed [7].

Detection of glucuronides with targeted and screening methods is hampered by the fact that glucuronides are only rarely available as authentic standards or hardly covered by spectral libraries. Therefore, during sample preparation, glucuronides are often converted by enzymatic or acidic hydrolysis to the corresponding aglycone [8, 9]. The application of such pre-treatment steps, in addition to the disadvantage of being work-intensive and reported to be less reproducible [10, 11], may also lead to unexpected and unintended alterations in the sample composition [12].

Some conjugates produce a specific neutral loss (NL) in tandem mass spectrometry [13], which can be utilized as an analytical tool to detect those species [6]. Neutral loss scans have already been applied for detecting glucuronides and sulfates [14–16] as well as mercapturic acids [17, 18] in biological samples.

Already in 2003, Yan and co-workers studied the in-source dissociation and fragmentation patterns of commercially available glucuronides and glucuronides generated from microsomal incubations [19]. They reported that “the neutral loss of *m/z* 176 was detected for all glucuronides”. Furthermore, they concluded that “in-source dissociation of glucuronides generates fragment ions identical to those of the precursor ions of the original drugs”. Due to the fact that the in-source fragments and the aglycones behave similarly in MS/MS experiments, unequivocal detection and quantification of the aglycones might be hampered, if the interfering species were not efficiently separated [20].

If the collision-induced dissociation (CID) of a glucuronide might give rise to the production of a set of fragment ions that represent a superset of the observed aglycone fragment ions, then it should be possible to (1) detect a glucuronide based on its specific neutral loss of *m*/*z* (C_6_H_8_O_6_) = 176.0321 and (2) convert the glucuronide spectrum into the aglycone spectrum for a subsequent annotation by MS/MS spectral library search.

Herein, we present an in silico workflow for annotating glucuronidated compounds in data-dependent tandem mass spectrometry datasets. This method combines (1) neutral loss screening to detect MS/MS spectra likely stemming from glucuronides, (2) deconjugation at the spectral level to produce aglycone-specific MS/MS spectra, (3) a retention time comparison between the known retention time of the unconjugated compound versus the measured glucuronidated feature and (4) MS/MS spectral library search. To better understand the instrumental requirements for a successful application of this method, we studied how fragmentation depends on the collision energy of reference glucuronide spectra. We applied and evaluated the method on two datasets in positive ionization mode containing in vitro– and in vivo–generated glucuronides: Solvent standard mixtures of chemicals were exposed to human liver S9 fractions with activated glucuronidase enzymes (in vitro) for glucuronide synthesis, and authentic urine samples were obtained from patients with known medication status that were participating in a clinical trial (in vivo). The statistical evaluation involved determination of false-negative and false-positive rates. Several reasons for false-negative detections of glucuronides with a detected signal of the expected *m*/*z* value in the full-scan analysis but revealing a non-matching library result with this method were identified.

## Material and Methods

### Basic in silico deconjugation procedure

The in silico deconjugation workflow for annotating glucuronidated compounds in data-dependent tandem mass spectrometry datasets consists of the following steps:

1. *Neutral loss screening*: During fragmentation of a glucuronide, the glucuronide moiety (C_6_H_8_O_6_) is cleaved from the precursor ion, with the positive charge remaining on the aglycone, leading to a specific neutral loss of *m*/*z* 176.0321. We applied the neutral loss screening for glucuronide-specific losses retrospectively on all acquired data-dependent MS/MS to determine those MS/MS spectra which contained the glucuronide-specific neutral loss and were therefore most likely stemming from a glucuronidated compound.
2. S*pectral modification*: The spectra related to glucuronides were truncated in a way that only fragments with *m*/*z* values lower than the expected precursor mass of the aglycone remained in the spectra.
3. *Spectral library search*: All truncated spectra were compared in a library search algorithm with a reference spectral library containing spectra of the aglycones. To this end, we changed the precursor *m*/*z* values in the respective spectra to those of the corresponding aglycones.

### Reference spectra of glucuronides

Reference spectra of glucuronides included in the “Wiley Registry of Tandem Mass Spectral Data” (WRTMSD) [21] were used as a training set to develop and optimize the in silico deconjugation workflow. These included spectra of codeine-6-glucuronide, oxazepam-glucuronide, naloxone-3-beta-d-glucuronide, dihydrocodeine-6-beta-d-glucuronide, dihydromorphine-3-beta-d-glucuronide, buprenorphine-3-beta-d-glucuronide and morphine-3-beta-d-glucuronide. The corresponding reference standards were obtained from Lipomed (Arlesheim, Switzerland). The reference spectra were acquired on a QqTOF instrument (TripleTOF 5600 + , Sciex, Toronto, Canada) as described elsewhere [22].

### Human liver S9 incubation and measurement

To generate a wider variety of spectra originating from glucuronidated compounds, nine mixtures of compounds including pharmaceuticals, pesticides, steroids, amines and industrial chemicals available in the laboratory were incubated at a concentration of each compound of 0.2 ng/mL. We used 5 µL of a highly pooled human liver S9 Mix (Sekisui Xenotech Xtreme, LLC, Kansas City, USA; containing 200 pooled human liver S9 fractions of mixed gender at a concentration of 20 mg/mL), 40 µg MgCl_2_ (purity > 98%, Merck) and 5 µg of alamethicin (Sigma-Aldrich) diluted with 95 µL of 0.05 M TRIS–HCl buffer (Alfa Aesar Co., Inc.) with pH 7.4. The cofactors were added with an incubation concentration of 1 M for uridine 5′-diphosphoglucuronic acid trisodium salt (UDPGA, purity > 98%, Sigma-Aldrich) and 0.2 M of reduced nicotinamide adenine dinucleotide phosphate (NADPH, Sigma-Aldrich). The mixtures were kept in an incubator (neoLab® neoMix 7–0921, Heidelberg, Germany) at 37.5°C for 5 h under constant shaking, and the reaction was stopped with an equivalent volume of methanol. The organic phase was removed under a gentle nitrogen stream at room temperature. A clean-up procedure was applied including solid-phase extraction (Strata-X Microelution SPE, Phenomenex, Aschaffenburg, Germany) with a method described elsewhere [23].

Liquid chromatography separation was achieved with a Thermo Ultimate 3000 LC system (Thermo Fisher Scientific, Waltham, USA) by gradient elution with water and methanol on a reversed-phase column (Waters UPLC BEH C18 1.7 μm, 2.1 mm × 100 mm, with pre-column) with a run time of 15 min followed by an isocratic run time of 6 min per sample. 0.1% of formic acid and 0.2% of 1 M ammonium formate were used as eluent additives. The temperature of the column was kept constant at 50 °C, and the flow rate was 300 μL/min. The injection volume was set to 50 µL.

Mass spectrometric detection was accomplished by a QExactive Plus instrument (Thermo Fisher Scientific, Waltham, USA) with a nominal resolving power of 70,000 (FWHM at *m*/*z* 200) and a scan range of 75–1000 m/*z* employing heated electrospray ionization (HESI) in positive ionization mode. The spray voltage was set to 3.5 kV. Auxiliary gas flow was set to 11, stealth gas flow to 48 arbitrary units. The capillary temperature was set to 256 °C, the auxiliary gas heater to 410 °C. Data-dependent acquisition control was used to acquire MS/MS data, using two experiments with collision energies of HCD 35 and 50 NCE with a resolving power of 35,000 (FWHM at *m*/*z* 200) and a scan range of 75–1000 m/*z*, using an inclusion list of the calculated *m*/*z* values for the expected [M-Gluc + H]^+^ ions. A duty cycle in the data-dependent acquisition mode included a single MS scan (maximum injection time of 200 ms) followed by four data-dependent MS/MS scans (maximum accumulation time, 50 ms each) and an isolation window of 1 m/*z*.

### Non-target analysis of authentic urine samples

Authentic urine samples were obtained from 51 patients participating in the PROVALID study, a non-interventional, prospective cohort study including 4000 patients with type 2 diabetes mellitus in five different European countries (Austria, Hungary, UK, Poland and The Netherlands). The study design and baseline characteristics of PROVALID are published elsewhere [24]. Signing an informed consent form was a prerequisite for study participation in all countries.

A volume of 100 µL of each urine sample was prepared by solid-phase extraction (Strata-X Microelution SPE, Phenomenex, Aschaffenburg, Germany) according to the manufacture instructions. The obtained eluate (50 µL) was diluted with 50 µL of water (+ 0.1% HFBA) before LC–MS analysis. A detailed description of the LC–MS/MS system and the operating conditions can be found in Hornek-Gausterer et al. [25]. The LC–MS/MS experiments were performed on a Waters ACQUITY UPLC (Waters, Manchester, UK) coupled to a TripleTOF 5600 + mass spectrometer (Sciex, Toronto, Canada). For chromatographic separations, a reversed-phase column (Kinetex 2.6 µm Biphenyl 100 Å, 100 × 2.1 mm, Phenomenex) with a 15 min linear gradient of 2–98% ACN in aqueous 0.5% acetic acid solution (v/v) was employed. The column temperature was 50 °C, and the applied flow rate was 200 μL/min. Sample aliquots of 7.5 µL were injected using the “partial loop overfill” mode.

Mass spectrometric detection was accomplished with positive electrospray ionization (ESI) using a DuoSpray ion source. The spray voltage was 5.5 kV. Gas flows of 10 arbitrary units for the nebulizer gas and 50 arbitrary units for the turbo gas were used. The temperature of the turbo gas was adjusted to 400 °C. An approximate mass resolving power of 30,000 for MS and 15,000 for MS/MS was used for operation and automatically recalibrated every ten sample injections using APCI positive calibration solution delivered via a calibration delivery system (Sciex, Darmstadt, Germany). The scan range was *m*/*z* 100–700 for MS and *m*/*z* 50–700 for MS/MS. The duty cycle in the data-dependent acquisition mode included a single MS scan (accumulation time, 100 ms) followed by eight dependent MS/MS scans (accumulation time, 100 ms each) in the high-sensitivity mode with dynamic background subtraction. The intensity threshold for switching to MS/MS experiments was set to 100 counts. MS/MS spectra were acquired at 35 ± 10 eV. Former target ions were excluded for 30 s after two occurrences.

### Data analysis

All samples were converted into .mgf and .mzML files using the ProteoWizard version v3.0.18265 function msconvert [26]. The data analysis workflow was performed in R version 4.0.3. All R code used during this study is available at https://github.com/chufz/deconjugatoR for reproducibility purposes. Each .mgf file was split into .txt files for each spectrum prior to the analysis. These .txt files were screened in parallel for the abundance of a NL in a mass window of ± 0.001 mu. We selected the loss of anhydroglucuronic acid (C_6_H_8_O_6_) with Δ*m*/*z* of 176.0321, which is specific for all glucuronides and which we will refer to as Gluc-NL. We additionally screened for a NL of Δ*m*/*z* 194.0426 mu, which we will refer to as GlucA-NL, since this loss of glucuronic acid (C_6_H_10_O_7_) was reported for glucuronides formed with benzylic or acylic bonds [13]. All spectra fulfilling these criteria were copied to a separate folder, the precursor mass was modified to the *m*/*z* value of the NL fragment, and the spectra were truncated above the newly assigned precursor. If an additional GlucA-NL was abundant, the respective fragment was also removed. We will refer to this data as in silico–deconjugated spectra in the following.

Tandem mass spectra library search was accomplished with “MSforID Search” [27, 28]. The search algorithm determines the similarity between a sample spectrum and library spectra. The estimation of similarity starts with the identification of fragment ions that are present in both of the spectra being compared. These ions are called “matching fragments”. The spectral information retrieved is used to calculate the “reference spectrum specific match probability” (*MP*). If the mass spectral library contains multiple spectra per reference compound, multiple *MP* values per reference compound are obtained. To combine all these compound-specific *MP* values to one value that specifies the similarity between the unknown and the specific reference compound, the compound-specific *MP* values are averaged and normalized to yield the compound-specific “average match probability” (*AMP*). The *AMP* values range between 0 and 100. A high compound-specific *AMP* value indicates high similarity between the unknown and the reference compound. The substance with the highest *AMP* value is considered to be the best library match.

As a reference library, the WRTMSD [21] was used in batch mode with a program written in Pascal using Delphi 6 for Windows (Borland Software Corporation, Scotts Valley, CA, USA; now Embarcadero Technologies, Inc., San Francisco, CA, USA). We applied a Δ*m*/*z* =  ± 0.01 and an intensity cut-off factor of 0.01 as search parameters. A library search annotation was considered as putatively correct if the precursor ion mass error was within ± 0.01 of the predicted ion mass and the *AMP* value was greater than 5.0. The correctness of tentative identifications was then checked by visually reviewing the head-to-tail plots. The resulting information of this spectral matching algorithm is presented as *AMP *values.

Full-scan MS^1^ screening and extraction of chromatograms for the predicted ion masses of all known or with the library approach detected compounds and their glucuronide metabolites was performed with the package peakPantheR [29] for calculating the signal intensities. Samples with a full-scan signal peak of a glucuronide metabolite, which were not detected by the spectral library approach, were further examined by extracting the MS/MS information and manually evaluating the spectra. Further manual structure elucidation and evaluation of the fragmentation trees was then performed with Sirius version 4.7.3 [30]. For further evaluation cases, dot product scores were calculated by using the compareSpectra() function in the spectra package [31]. We refer to this value as *scores* in the further description.

## Results and discussion

### Evaluating the impact of the collision energy with QqTOF reference spectra

For successful application of the in silico deconjugation approach, the presence of the fragment generated by the Gluc-NL in the glucuronide-specific MS/MS spectrum is necessary. The detection of the aglycone-specific fragment ion enables identification of a glucuronide and assignment to the corresponding aglycone by spectral library search. In MS/MS, the collision energy is the most important parameter for controlling signal intensities in fragment ion mass spectra. For the presented in silico deconjugation approach, the collision energy should be sufficiently high to enable the Gluc-NL and further fragmentation pattern of the molecule for a successful spectral matching. However, a complete decomposition of the aglycone-specific fragment should be avoided.

**Fig. 1:**
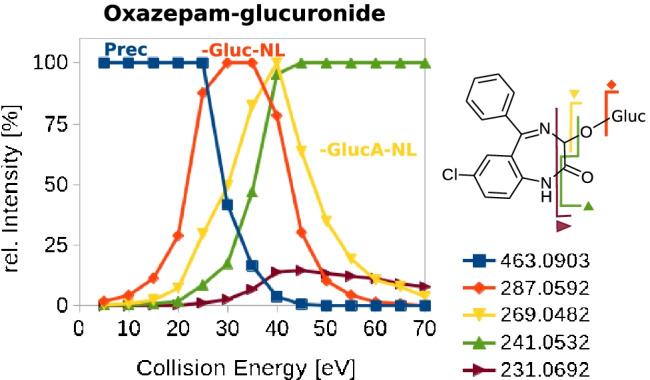
Impact of the collision energy on the relative signal intensity of the five most prominent fragments of oxazepam-glucuronide acquired on a QqTOF instrument

We evaluated the impact of collision energy settings on the fragmentation patterns of glucuronides and the corresponding aglycones with a training set taken from an established MS/MS spectral library (WRTMSD) [21]. The glucuronide spectra of seven different compounds were acquired on a QqTOF instrument by CID employing different collision energies of 5–70 eV. Figure 1 shows the relative signal intensity of the five most prominent fragments for oxazepam-glucuronide as a function of the collision energy. The corresponding figures for the other studied compounds are given in the SI, Figures S1–6. For all compounds, we could observe a fragment associated to the Gluc-NL. The minimum energy needed for a visible Gluc-NL (above 10% of the base peak intensity) ranged from 25 to 30 eV. For the glucuronide of naloxone, 30% of the relative base peak intensity was already observed for the fragment of the aglycone at a collision energy of 20 eV. For buprenorphine, we observed the same relative signal intensity not until energy values rose above 35 eV.

However, high collision energies may cause a loss of the fragment associated to the Gluc-NL, which will result in a non-detection of the glucuronide. For high collision energies, we observed decreasing intensities for the Gluc-NL fragment, but only for the compound oxazepam a complete loss of the fragment ion signal was observed above 65 eV.

**Fig. 2:**
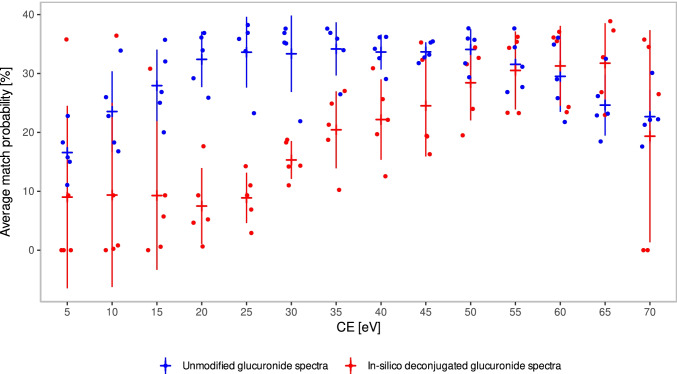
Effect of the collision energy on the average *AMP *values obtained in a spectral library search for the in silico deconjugated glucuronide spectra (red) and the unmodified glucuronide spectra (blue), which were all present in the library obtained on the same QqTOF instrument (*N* = 5). Mean and ± standard deviation are added

Figure 2 compares the *AMP *values for five glucuronides measured at different collision energies for a library search of the in silico–deconjugated spectra and the unmodified glucuronide spectra, for which entries in the library on the same instrumentation were available. The unmodified glucuronide spectra show a distribution of *AMP *values which is typical for a small molecule library search [32], with lower values for very low and high collision energies, but rather comparable values for a broad range from 20 to 55 eV. For the in silico deconjugation approach, we observed increasing matching values with increasing collision energies. Similar *AMP *values as for the conventional library search are only obtained above 50 eV. This can be explained by the fact that a significant part of the internal energy is absorbed during the fragmentation process and therefore is lost with the fission of the glucuronide bond. This results in a decrease in the overall fragmentation efficiency of the remaining part of the molecule, and therefore, a higher overall collision energy is required to obtain diagnostic fragments resulting in a higher match value.

To summarize, for the proposed in silico deconjugation approach followed by a conventional library search using collision energies in the medium range around CE 35 ± 20 eV, we conclude an applicability of this method due to the expected presence of the fragment associated to the Gluc-NL in QqTOF datasets and eminent fragmentation of the molecule to gain sufficient spectral matching values.

### Proof of concept with Q-Orbitrap spectra

To evaluate the applicability of the in silico deconjugation approach with spectra generated on a Q-Orbitrap, in vitro incubation experiments were used to generate glucuronides of different molecular structures. To avoid toxicity effects, the reference mixture concentrations were kept low; therefore, only glucuronides of compounds with high conjugation activity are expected to be found. Out of nine in-house reference mixtures containing in total 660 compounds incubated in vitro, we could in total extract 148 MS/MS spectra assigned to 33 glucuronidated compounds. All glucuronides were only detected in the incubations of the mixtures with the aglycone being present, leading to the conclusion that no false positives are introduced by this method. Literature reports of glucuronide conjugation reaction of all detected glucuronidated drugs and most of the other chemicals support our findings (Table S1 in SI). Head-to-tail plots are depicted in the SI, Figures S7–39. For all of these compounds, reference spectra for the aglycone were available through MassBank [33] or the WRTMSD [21]. Fragmentation was accomplished with higher-energy collisional dissociation (HCD) employing two different normalized collision energies (NCE) of 35 and 50, which are values typically used on this type of instrument for the LC–MS/MS screening of small molecules [32].

For 17 of the detected glucuronides, the fragment associated with the Gluc-NL was present in both applied collision energies. For nine of the compounds, the spectra with higher collision energies (HCD 50 NCE) showed no fragment related to Gluc-NL, but was still present at HCD 35 NCE. For the glucuronides of montelukast, imatinib, losartan, the trifloxystrobin metabolite CGA 321113, bezafibrate, clotrimazole and tebuconazole, no Gluc-NL was observed for both collision energies applied (HCD 35 and 50 NCE), but a spectral match with the aglycone still validates the glucuronide conjugate being present. The presence of a NL of the glucuronide moiety is mandatory for a successful filtering of spectra from probable glucuronide metabolites in the envisaged strategy. We therefore conclude for datasets acquired by Q-Orbitrap involving HCD to include lower collision energies (HCD < 35 NCE), if the in silico deconjugation approach should be applied.

For all spectra associated with glucuronides present in the mixtures, we revealed for the library search with in silico–deconjugated spectra an average dot product score of 0.78 and an average *AMP *of 30. Best spectral matching was observed for 1,2-benzoisothiazolinone (dot product 0.98, *AMP *52), while the lowest score was achieved for 4-aminoazobenzene (dot product 0.4, *AMP* 27). For all in silico–deconjugated spectra generated with HCD 50 NCE, higher spectral matching scores were obtained as compared to those generated with HCD 35 NCE. This observation is consistent with the QqTOF results reported above.

Tandem mass spectra acquired at HCD < 35 NCE enable efficient glucuronide detection by neutral loss screening. Tandem mass spectra acquired at HCD > 35 NCE show better performance in spectral matching to aglycon reference spectra. Ideally, for each feature, spectra acquired at low- and high-NCE settings should be available. With a Q-Orbitrap, this can be accomplished by repeated analysis of a sample, or in one run by applying the stepped collision energy option.

### Annotation of glucuronides in authentic urine samples

As part of a study on medication adherence within the PROVALID study, 51 authentic urine samples were screened for pharmaceutical compounds and their metabolites. The results were used to assess medication compliance of the corresponding patients. The analysis was performed on a QqTOF instrument, with data-dependent acquisition for non-targeted analysis in positive ionization mode. Overall, by applying the in silico deconjugation approach, glucuronides related to 43 pharmaceutical compounds were annotated in this dataset. The detection rate (DR) of the individual glucuronide species found in the dataset are plotted in Fig. 3. A detailed overview of the signal intensities and *AMP *values retrieved from tandem mass spectral library search are given in the SI, Tables S2 and S3. Head-to-tail plots of the spectral comparison for the best matching spectra are given in SI Section S6, Figures S40–84.

**Fig. 3:**
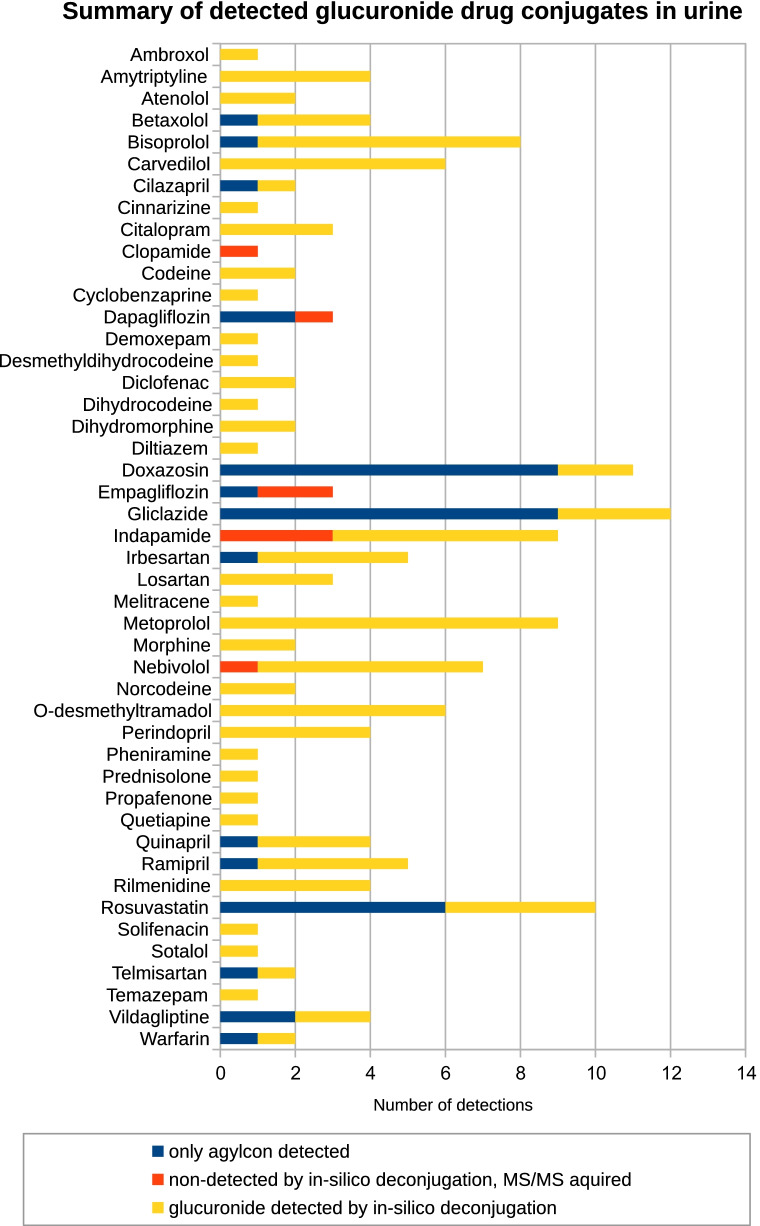
Number of detections of (i) glucuronidated drugs in the analysed urine dataset of 51 patients by the in silico deconjugation approach (yellow), (ii) manually assigned glucuronides based on MS/MS spectra which were not detected by this method (orange) and (iii) aglycones based on spectral library search, for which no spectral MS/MS information for the glucuronide was generated (blue)

Besides glucuronides related to pharmaceutical compounds, we have further detected glucuronides of the steroids 1,7-hydroxyprogesterone (DR 86%) and 6-beta-hydroxytestosterone (DR 51%), the flavonoid catechin (DR 8%), and the isoflavonoid genistein (DR 18%), as well as the cinchona alkaloids quinine (DR 2%) and hydroquinine (DR 2%). For all identified glucuronides, the best spectral match resulting in the highest *AMP* values for the in silico deconjugation approach was obtained for quetiapine and telmisartan with an *AMP *value of 48. The average *AMP* for all detected glucuronides was 23, which was lower than the average *AMP* for the aglycones or metabolites of 34. With the exception of four drugs (melitracen, clopamide, quinapril and rilmenidine), the excretion of a glucuronidated metabolite was already reported in the literature (see references in Table S2–3). Except for ramipril in two samples, for all glucuronides, either the aglycone or another metabolite of the corresponding drug (perindoprilat for perindopril and the hydroxy-metabolite for nebivolol) was found in exactly the same samples as the glucuronide. Still, we observed a low MS^1 ^signal abundance of ramipril and ramiprilat in the same detection pattern among the samples. We therefore assume that the samples with a glucuronide detection of a specific drug stem from a consumption thereof. Thus, we concluded that no false-positive detections arose applying the in silico deconjugation approach.

There are three reasons for false-negative results retrieved by our method. Non-detection of glucuronides resulted either from the absence of any MS/MS spectrum, from the missing of the specific neutral loss (step 1: neutral loss screening), or from insufficient similarity of the modified tandem mass spectrum with the corresponding aglycone reference spectra (step 3: spectral library search).

Considering the glucuronides which were detected at least in one of the analysed samples, we screened the remaining samples for a MS^1^ signal of same *m*/*z* and retention time. In 20 cases, extracted ion chromatograms indicated the presence of a confirmed glucuronide. In none of these cases was tandem mass spectral information available to verify the MS^1^ annotation.

To further identify possible false-negative results, we evaluated the known drug prescription of the PROVALID study whether additional glucuronide conjugates could be present in other samples by full-scan MS^1^ EIC extraction. For five of the prescribed drugs (amlodipine, benazepril, urapidil, ticagrelor and duloxetine), a low MS^1^ signal could be detected in 11 cases, but no data-dependent MS/MS spectra were acquired except for duloxetine in one sample. In that case, the obtained MS/MS spectrum was impacted by impurities.

For five glucuronidated compounds assigned by the detection of a neutral loss (dapagliflozin, empagliflozin, indapamide, clopamide, nebivolol), the spectral matching of the corresponding eleven spectra showed *AMP *values < 5 and consequently these were considered as not detected by the in silico deconjugation method. These spectra were manually reviewed and confirmed as glucuronidated compounds with a deviating fragmentation pattern in the in silico deconjugation approach, which is further described below.

The number of false-negative results due to missing MS/MS spectra (*N* = 20) was much higher than the number of false-negative results retrieved due to problems related to the in silico deconjugation approach (*N* = 12). This observation indicates that the in silico deconjugation method can reliably detect glucuronides by converting and matching the respective tandem mass spectral data.

### Molecular structures with a deviating fragmentation

In some instances, we found abundant fragment ions that contain parts of glucuronide or the glucuronide group as a whole. As long as their *m*/*z* values are larger than the *m*/*z* values of the corresponding aglycones, the fragments are removed from the in silico–deconjugated spectrum. If the *m*/*z* values are smaller, the fragments remain in the truncated spectrum as unwanted interference. To estimate the likelihood for occurrence of fragments only observed in the glucuronide spectra, all available in silico–deconjugated spectra were examined for interfering fragment ions. In total, 16 molecular structures were found with prominent (> 5% of base peak intensity) fragments being present only in the spectra corresponding to the glucuronidated compound, but not in the reference spectra of the aglycone.

**Fig. 4:**
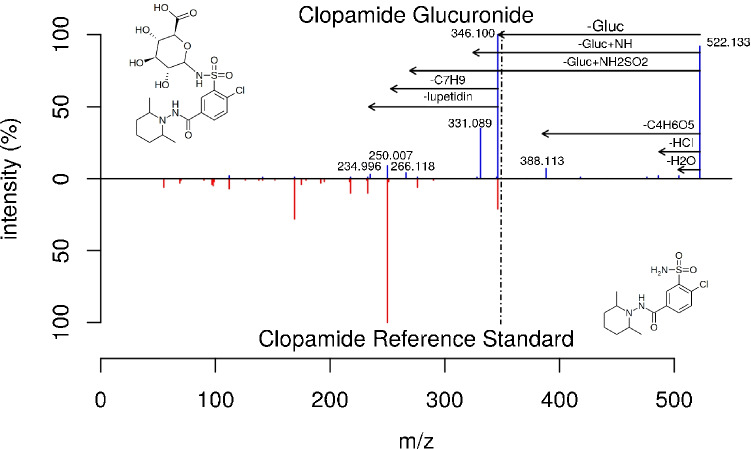
Head-to-tail plot to compare the best matching reference spectra of clopamide (CE 35) and clopamide-glucuronide from the authentic urine dataset as an example for a conjugated sulfonamide, acquired on a QqTOF instrument. The conjugation on the sulfonamide group results in a different fragment by the NL of [Gluc + NH], leading to a decreased spectral similarity

The occurrence of additional fragments led to a decrease in the scoring value of the spectral match and in four cases for two compounds (indapamide and clopamide) to non-detections. Both compounds contain a sulfonamide group, which is most likely the functional group being conjugated [34]. Figure 4 shows the comparison of the in silico–deconjugated spectra for clopamide-glucuronide and the reference spectra. Here, we could observe a fragmentation with a remaining amine group on the glucuronide moiety. We can explain this fragmentation by a fission of the S–N bond instead of the N–O bond to the glucuronide, leading to this additional fragment for conjugated sulfonamides. The same fragmentation was also observed for indapamide.

The drugs dapagliflozin and empagliflozin, both containing a glucuronide group in the parent molecule, were detected as di-glucuronides, but the di-glucuronidation resulted in a different fragmentation. Both compounds showed no similarity of the in silico–deconjugated spectra to the reference spectra.

**Fig. 5:**
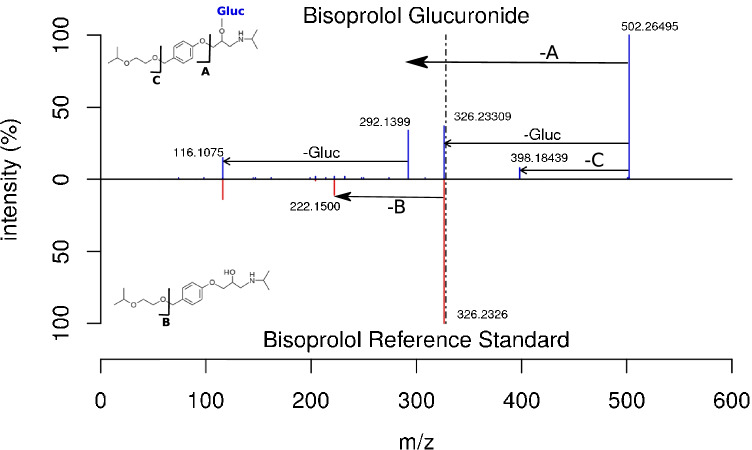
Head-to-tail plot to compare the best matching reference spectra of bisoprolol (CE 15) and bisoprolol-glucuronide from the authentic urine dataset acquired on a QqTOF instrument. It was chosen as an example for a glucuronide containing a weak ether bond in beta-position to the conjugated hydroxy-group, leading to a different fragmentation [-A] and therefore a low spectral similarity

Additional fragments in the in silico–deconjugated spectra were also formed in the case of bisoprolol and vildagliptine with base peak intensity. As a consequence, *AMP *values were considerably lower than for the compounds with the conventional fragmentation pattern. All beta blockers under consideration including atenolol, betaxolol, bisoprolol, carvediol, metoprolol, nebivolol and propafenon have an ether group in common, which is presumably a weak bond in ß-position to the hydroxyl group that acts as the likely position of conjugation [35]. This resulted in (I) a fission of the ether bond with a neutral loss larger than 176.0321 or (II) a heterolytic cleavage with the positive charge remaining on the part of the molecule with the glucuronic acid conjugate attached. As an example, this is shown for bisoprolol in Fig. 5. Thus, the observed fragmentation pattern does not resemble that of the corresponding aglycones. All beta blockers were still detected by the spectral library search, as the neutral loss of 176.0321 was visible as well, but with a decreased *AMP *value for the match due to the additional fragments in the spectra. For vildagliptine, the GlucA-NL was observed to generate the base peak of the in silico–deconjugated spectrum (Figure S64), which decreased the spectral similarity, if not removed. For bezafibrate, doxazosin, gliclazide, irbesartan and cilazapril (see respective Figures S11, S50, S51, S53 and S44 in SI), the observed deviating fragmentation could not be explained.

### Retention time correlation between glucuronide and aglycone

**Fig. 6:**
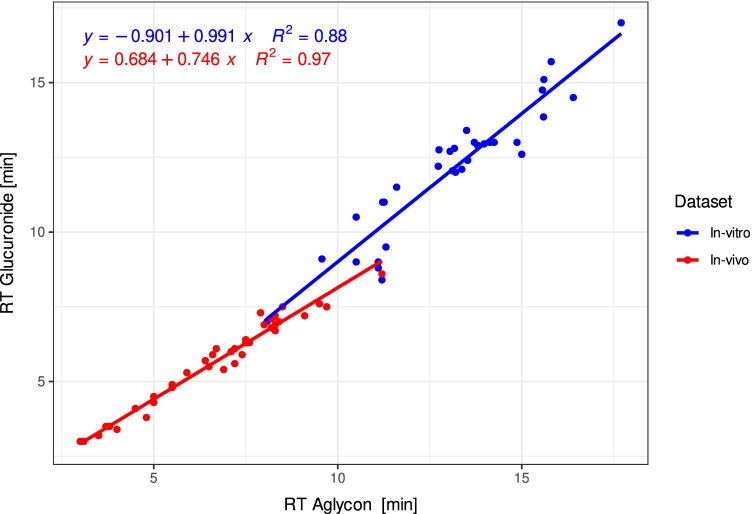
Retention time correlation of all detected glucuronides in the measured in vitro dataset measured with a C-18 column (BEH C18 1.7 µm, 100 mm × 2.1 mm, Waters) and the in vivo dataset measured with a biphenyl column (Kinetex 2.6 µm Biphenyl 100 Å, 100 mm × 2.1 mm, Phenomenex)

Beyond spectral similarity values, retention times are often used for datasets measured by liquid chromatography for increasing the confidence in compound identification. We therefore studied the relation of the retention times of the glucuronides to the aglycone for both the in vitro and in vivo datasets, which involves two different liquid chromatography systems. In both systems, we observed a chromatographic separation between glucuronide and aglycone for all detected glucuronides. We revealed a linear correlation (*R*^2^ = 0.88, Fig. 6) and an even better correlation for the annotated glucuronides in the in vivo dataset (*R*^2^ = 0.97, Fig. 6). We observed a mean shift of the glucuronide signal compared to the aglycone of − 0.97/ − 1.02 min (in vitro/in vivo dataset). These close relationships suggest that it is possible to use retention time information to exclude false positives and to predict the retention time of the corresponding glucuronide based on the known retention time of the aglycone.

## Conclusions

With both studied datasets acquired on different instruments in positive ionization mode, we proved the applicability of an in silico deconjugation method for the annotation of glucuronide conjugates with unconjugated reference library entries for screening purposes. The selectivity of the approach is very high, as no false positives were observed in the evaluation of a dataset of authentic urine samples from patients with known drug prescription. False negatives might arise, if either (i) no MS/MS scan was triggered to search for the neutral loss or (ii) the neutral loss fragment is not present in the MS/MS scan or (iii) some glucuronides show different fragmentation patterns depending on molecular structures and position of the charge. The spectral library match quality of the in silico deconjugation method is sufficient for many of the studied compounds. In contrast, for compounds showing a different fragmentation pattern, among them clopamide, spectral match quality drops, resulting also in false negatives depending on the defined minimum match score. However, with an availability of larger data MS/MS datasets, machine learning approaches could be trained to predict from the spectra entries the probability of fingerprints of molecular structures resulting in a deviating fragmentation.

The choice of appropriate MS/MS experimental settings has to consider that the fragment caused by the NL of the glucuronide should be present, but also the remaining part of the molecule should be fragmented to yield a considerable number of fragment ions for a reliable library search. Thus, stepped or ramped CE or two different MS/MS experiments with different CEs are suggested to improve the performance and sensitivity of the approach.

Although this proof-of-concept study focused on the application of the in silico deconjugation approach for the detection of modified pharmaceutical compounds with positive ionization mode detection, we assume that the in silico deconjugation approach can also be employed in negative ionization mode, as a neutral loss of *m*/*z* 176.0321 (C_6_H_8_O_6_) was also reported [13]. Furthermore, its applicability to other compound classes, including endogenous compounds, is anticipated.

Overall, the proposed method greatly enhances the capabilities for the annotation of glucuronides in complex biological and environmental samples.

## Supplementary Information

Below is the link to the electronic supplementary material.

Supplementary file1 (PDF 1827 KB)

## Data Availability

All R code used during this study is available at https://github.com/chufz/deconjugatoR.
