# Supplementary material for: In silico deconjugation of glucuronide conjugates enhances tandem mass spectra library annotation of human samples
Source: Anal Bioanal Chem. 2022 Jan 26;414(8):2629–40. doi: 10.1007/s00216-022-03899-7 (PMC8888480; doi:10.1007/s00216-022-03899-7)
Supplement: Supplementary file 1 — Supplementary file1 (PDF 1827 KB) [file 216_2022_3899_MOESM1_ESM.pdf]

Supporting information
In-Silico Deconjugation of Glucuronide Conjugates Enhances Tandem Mass Spectra Library Annotation of Human Samples

Carolin Huber,[*,†,‡] Martin Krauss,[†] Vera Reinstadler,[¶] Sara Denicolò,[§] Gert Mayer,[§] Tobias Schulze,[†] Werner Brack,[†,‡] and Herbert Oberacher[*,¶]

†*Department of Effect-Directed Analysis, Helmholtz Centre for Environmental Research - UFZ, Permoserstraße 15, 04318 Leipzig, Germany.*

‡*Institute of Ecology, Diversity and Evolution, Goethe University Frankfurt Biologicum, Campus Riedberg Max-von-Laue-Str. 13 60438 Frankfurt am Main, Germany.*

¶*Institute of Legal Medicine and Core Facility Metabolomics, Medical University of Innsbruck, 6020 Innsbruck, Austria.*

§*Department of Internal Medicine IV (Nephrology and Hypertension), Medical University of Innsbruck, 6020 Innsbruck, Austria.*

E-mail: carolin-elisabeth.huber@ufz.de; herbert.oberacher@i-med.ac.at

Contents

S1 Dependency of the collision energy **S3**

S2 Descriptive statistics table of found glucuronides in the in-vitro generated data set **S7**

S3 Head-tailplots of found glucuronides in S9-incubated mixtures **S9**

S4 Descriptive statistics table of found glucuronides in urine data set **S26**

S5 Head-tailplots of detected glucuronidated pharmaceuticals in urine data set **S28**

References **S51**

S1 Dependency of the collision energy

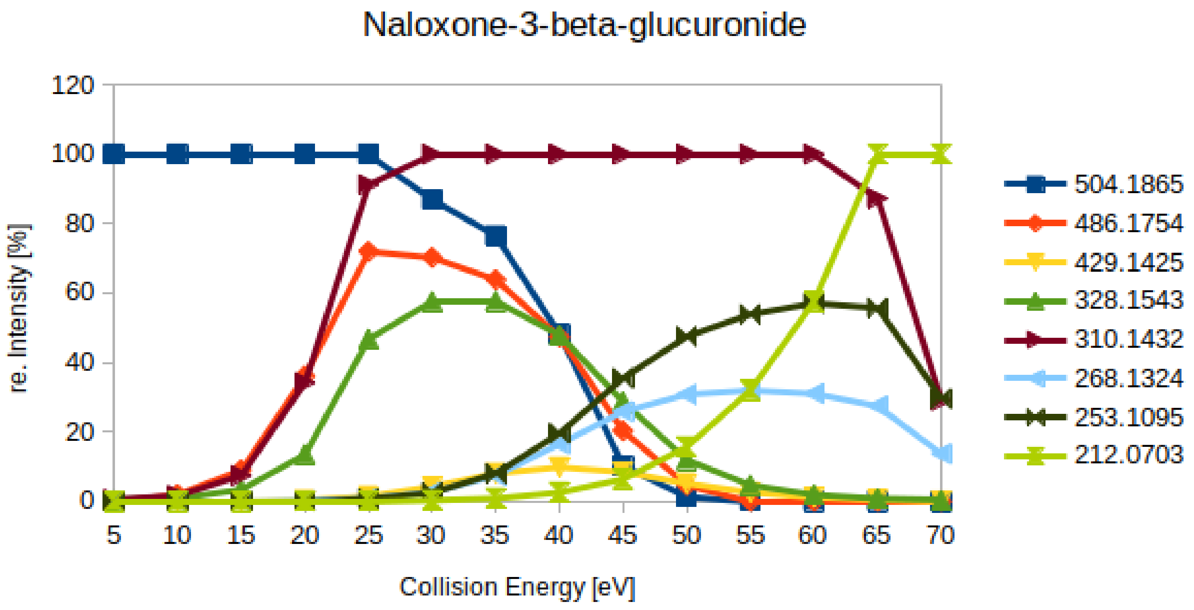

Figure S1: Dependency of the six most prominent fragments of naloxone-3-beta-glucuronide on the collision energy acquired on a QqTOF instrument.

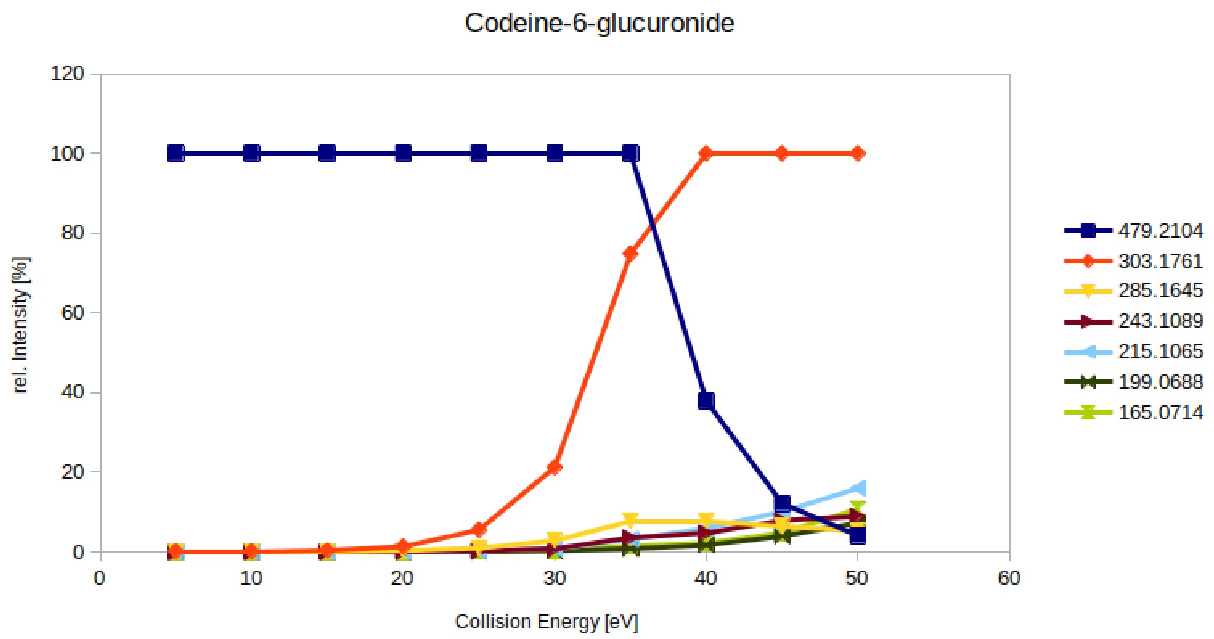

Figure S2: Dependency of the six most prominent fragments of codeine-6-glucuronide on the collision energy acquired on a QqTOF instrument.

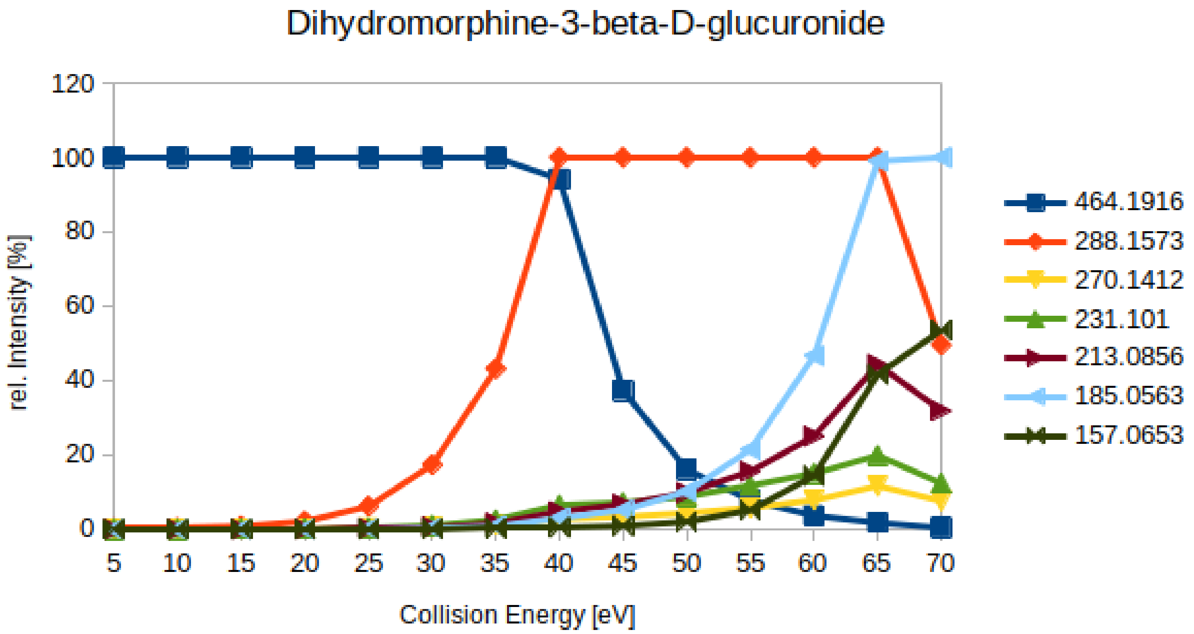

Figure S3: Dependency of the six most prominent fragments of dihydromorphine3-beta-D-glucuronide on the collision energy acquired on a QqTOF instrument.

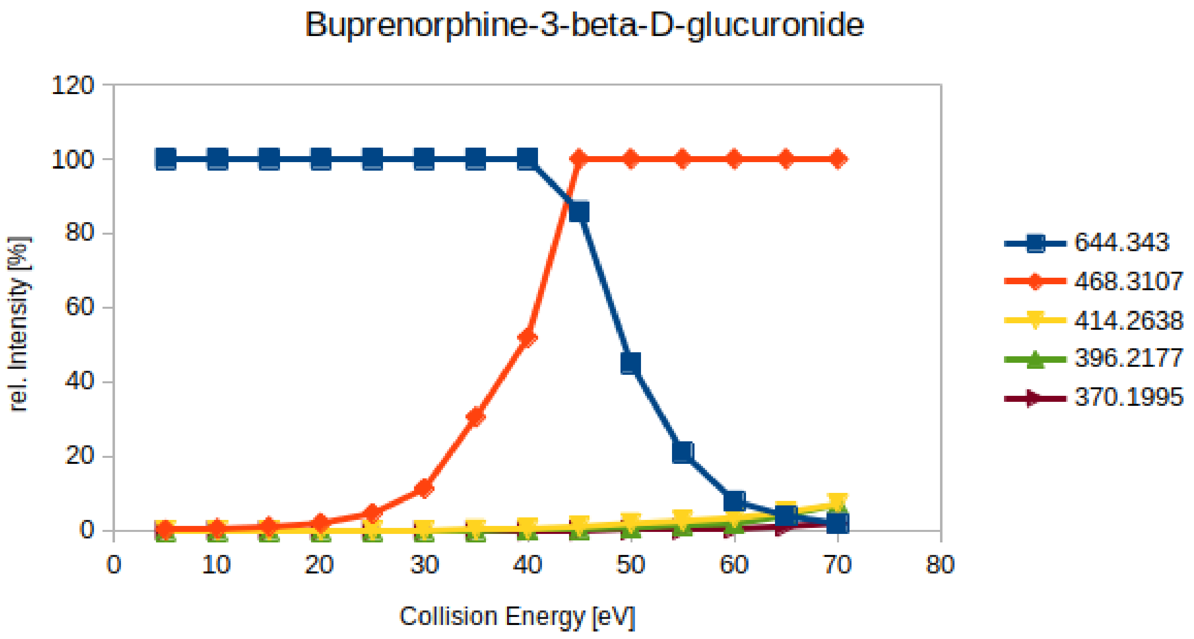

Figure S4: Dependency of the six most prominent fragments of buprenophine-3-beta-D-glucuronide on the collision energy acquired on a QqTOF instrument.

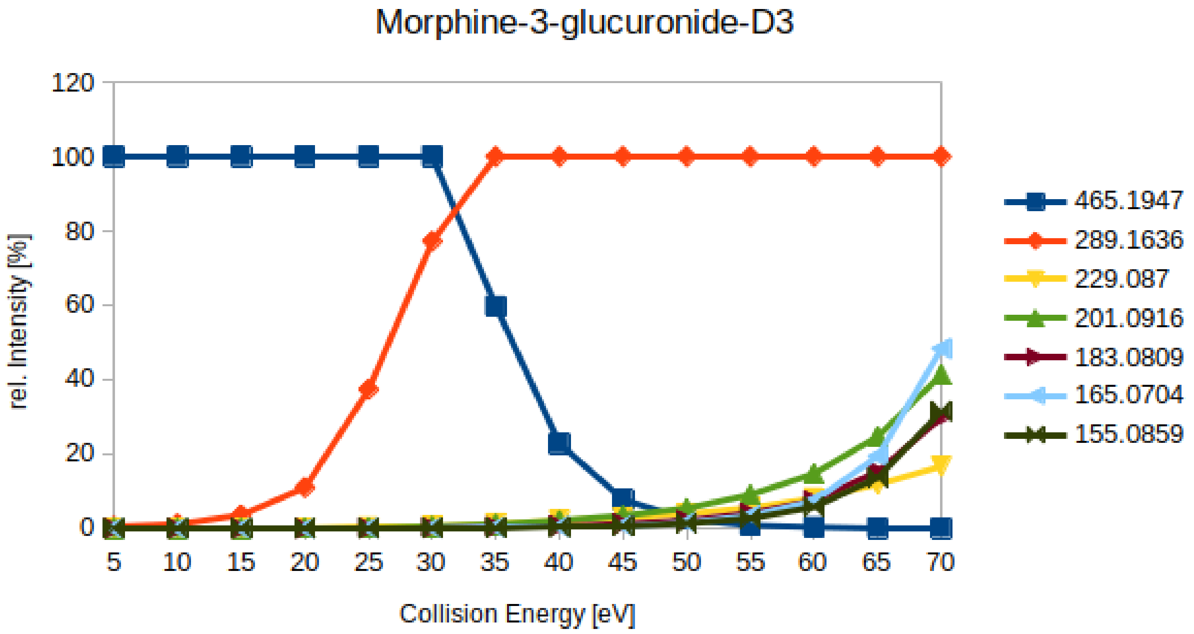

Figure S5: Dependency of the six most prominent fragments of morphine-3-glucuronide-D3 on the collision energy acquired on a QqTOF instrument.

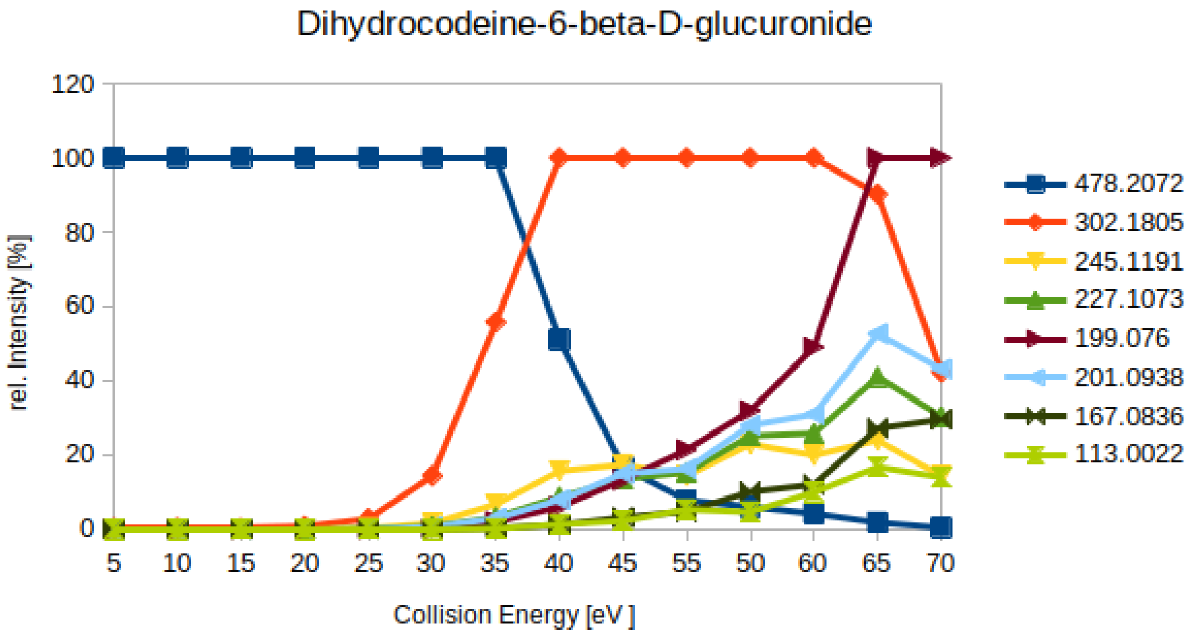

Figure S6: Dependency of the six most prominent fragments of dihydrocodeine-6-beta-D-glucuronide on the collision energy acquired on a QqTOF instrument.

S2 Descriptive statistics table of found glucuronides in the in-vitro generated data set

Table S1: Descriptive statistics of parent and conjugated drugs, including signal intensity, theoretical precursor mz (Prec m/z) for the [M+H]+ ion, retention time (RT), dot product score and the average match probability of the spectral library search algorithm.

**Compound**	**Signal Intensity**	Prec m/z	**RT** [min]	**Dot product**	**AMP**
**1,2-Benzisothiazolinone**	1.80E+06	152.0170	10.50		
1.2-Benzisothiazolinone glucuronide	4.91E+06	328.0491	9.00	0.98	52.00
**4-Aminoazobenzene**	1.10E+04	198.0998	8.08		
4-Aminoazobenzene glucuronide[1]	4.60E+03	374.1319	7.01	0.40	27.00
**Amitriptyline**	4.17E+08	278.1908	13.05		
**Amitriptyline glucuronide**[2]	1.78E+07	454.2229	12.70	0.81	37.00
**Azelastine**	2.68E+08	382.1686	12.75		
Azelastine glucuronide	3.36E+05	558.2007	12.75	0.86	8.42
**Bezafibrate**	6.80E+07	362.1158	13.98		
Bezafibrate glucuronide[3]	3.60E+05	538.1479	12.95	0.63	1.58
**Climbazole**	1.14E+08	293.1056	13.12		
Climbazole glucuronide	2.47E+06	469.1377	12.05	0.84	27.67
**Clotrimazole**	6.95E+04	345.1158	13.80		
Clotrimazole glucuronide[4]	3.37E+07	521.1479	12.90	0.88	24.71
**Daidzein**	1.58E+07	255.0657	11.20		
Daidzein glucuronide[5]	2.25E+06	431.0978	8.40	0.78	18.88
**Diphenhydramine**	6.60E+08	256.1701	11.60		
Diphenhydramine glucuronide[6]	1.17E+07	432.2022	11.50	0.93	11.99
**Epoxiconazole**	1.31E+08	330.0809	14.87		
poxiconazole glucuronide[7]	8.18E+05	506.1130	13.00	0.93	36.12
**Icaridin**	1.51E+08	230.1756	13.71		
Icaridin glucuronide	2.02E+07	406.2077	13.00	0.80	76.68
**Imatinib**	1.32E+08	494.2668	11.10		
Imatinib glucuronide[8]	2.69E+05	670.2989	8.80	0.50	25.02
**Imazalil**	1.52E+08	297.0561	12.73		
Imazalil glucuronide	5.55E+06	473.0882	12.20	0.66	32.50
**Ketoconazole**	9.75E+07	531.1565	13.17		
Ketoconazole glucuronide	9.75E+05	707.1886	12.80	0.75	19.44

Table S1 continued

**Compound**	**Signal Intensity**	Prec m/z	**RT** [min]	**Dot product**	**AMP**
**Labetalol**	1.71E+08	329.1865	10.50		
Labetalol glucuronide[9]	1.75E+06	505.2186	10.50	0.70	17.65
**Losartan**	7.18E+07	423.1700	13.50		
Losartan glucuronide[10]	1.54E+06	599.2021	13.40	0.43	7.47
**Mefenamic-acid**	1.44E+07	242.1180	16.40		
Mefenamic-acid glucuronide[11]	8.58E+07	418.1501	14.50	0.88	14.69
**Miconazole**	5.94E+06	414.9971	15.60		
Miconazole glucuronide	1.06E+06	591.0292	15.10	0.83	61.54
**Mirtazapine**	6.31E+08	266.1657	9.57		
Mirtazapine glucuronide[12]	8.03E+05	442.1978	9.10	0.94	3.99
**Montelukast**	3.61E+06	586.2182	17.70		
Montelukast glucuronide[13]	9.82E+05	762.2503	17.00	0.72	36.52
**Oxazepam**	4.89E+07	287.0587	13.20		
Oxazepam glucuronide[14]	5.56E+05	463.0908	12.00	0.91	18.65
**Pioglitazone**	3.50E+08	357.1273	11.10		
Pioglitazone glucuronide[15]	4.20E+05	533.1594	9.00	0.59	15.32
**Propranolol**	6.45E+08	260.1650	11.22		
Propranolol glucuronide[16]	9.35E+05	436.1971	11.00	0.69	11.40
**Prothioconazole-desthio**	9.16E+07	312.0670	15.00		
Prothioconazole-desthio glucuronide[17]	1.93E+06	488.0991	12.60	0.88	42.80
**Raloxifene**	9.89E+06	474.1739	11.31		
Raloxifene glucuronide[18]	5.25E+07	650.2060	9.50	0.70	61.02
**Tamoxifen**	1.80E+07	372.2327	15.80		
Tamoxifen glucuronide[19]	2.16E+06	548.2648	15.70	0.86	33.72
**Tebuconazole**	3.43E+08	308.1529	15.59		
Tebuconazole glucuronide[20]	3.99E+05	484.1850	13.85	0.83	11.05
**Telmisartan**	3.16E+07	515.2447	14.14		
Telmisartan glucuronide[21]	6.59E+07	691.2768	13.00	0.70	48.01
**Temazepam**	1.52E+08	301.0743	13.53		
Temazepam glucuronide[22]	1.68E+06	477.1064	12.40	0.93	40.54
**Testosterone**	1.08E+08	289.2167	14.25		
Testosterone glucuronide[23]	8.19E+06	465.2488	13.00	0.51	33.85
**Tetracain**	4.86E+07	265.1916	11.26		
Tetracain glucuronide	3.86E+05	441.2237	11.00	0.56	29.05
**Trenbolone**	3.68E+08	271.1698	13.37		
Trenbolone glucuronide[24]	1.40E+06	447.2019	12.10	0.48	52.55
**Trifloxystrobin-CGA-321113**	8.80E+07	395.1218	15.56		
Trifloxystrobin-CGA-321113 glucuronide	1.86E+06	571.1539	14.75	0.78	47.82

S3 Head-tailplots of found glucuronides in S9-incubated mixtures

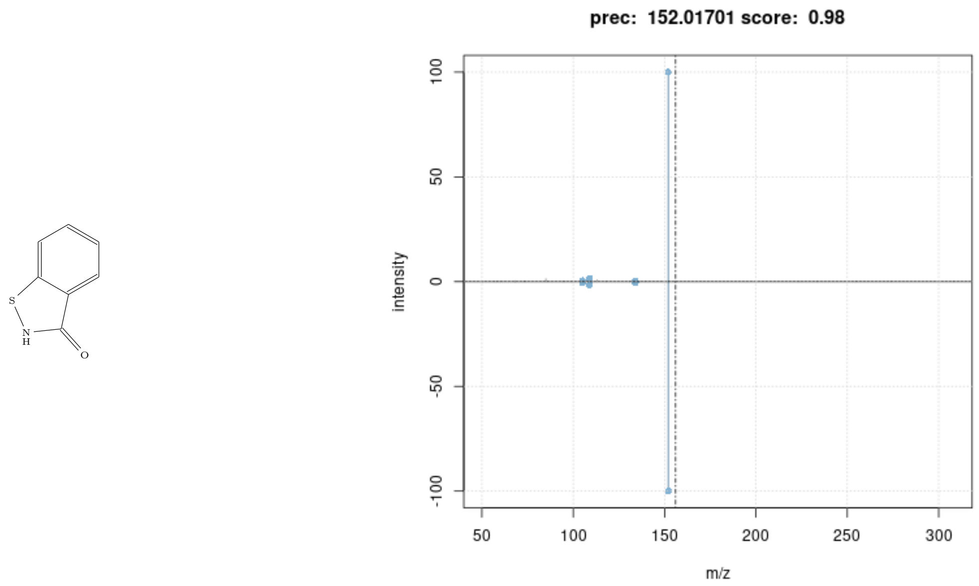

Figure S7: Spectra comparison between in-silico deconjugated spectra and reference spectra for 12-benzisothiazolinone.

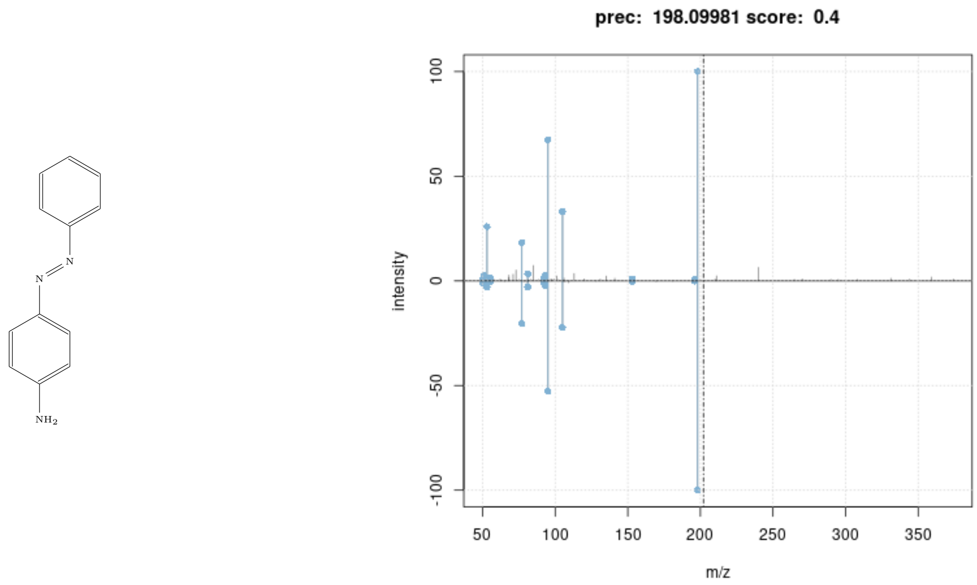

Figure S8: Spectra comparison between in-silico deconjugated spectra and reference spectra for 4-aminoazobenzene.

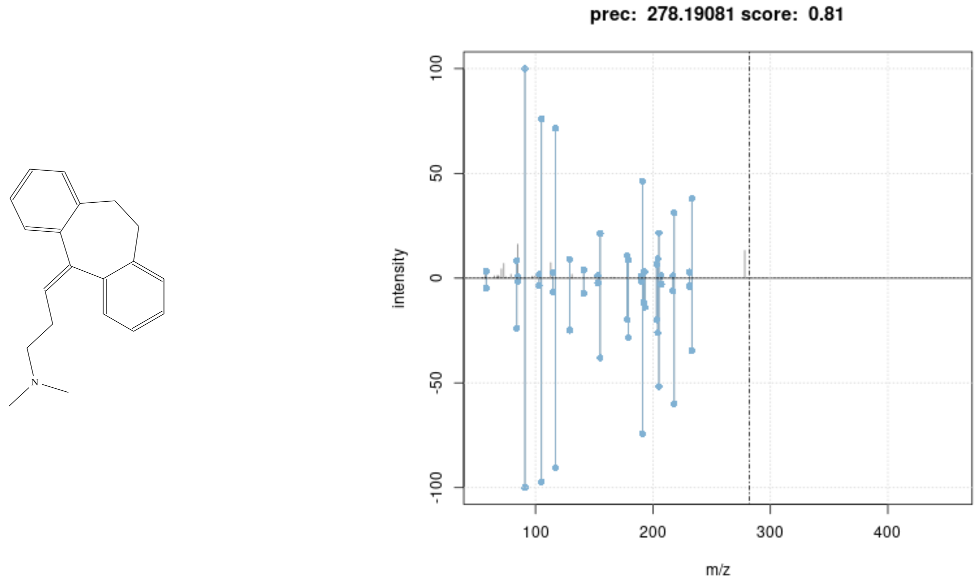

Figure S9: Spectra comparison between in-silico deconjugated spectra and reference spectra for amitriptyline.

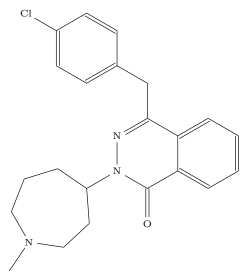

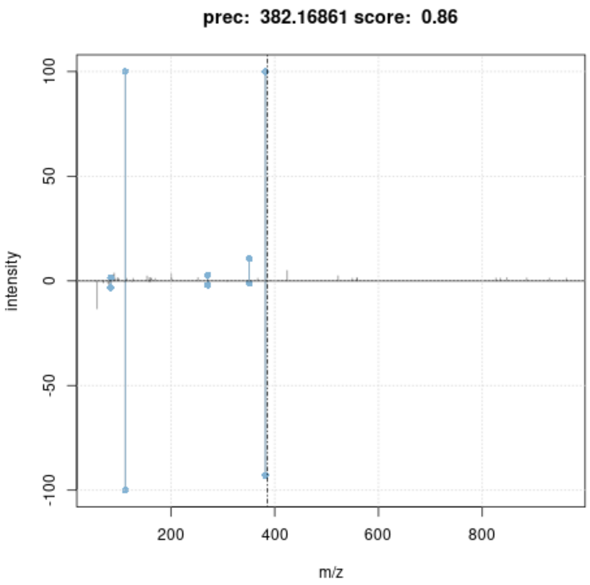

Figure S10: Spectra comparison between in-silico deconjugated spectra and reference spectra for azelastine.

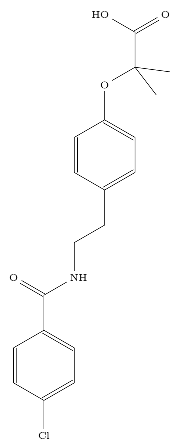

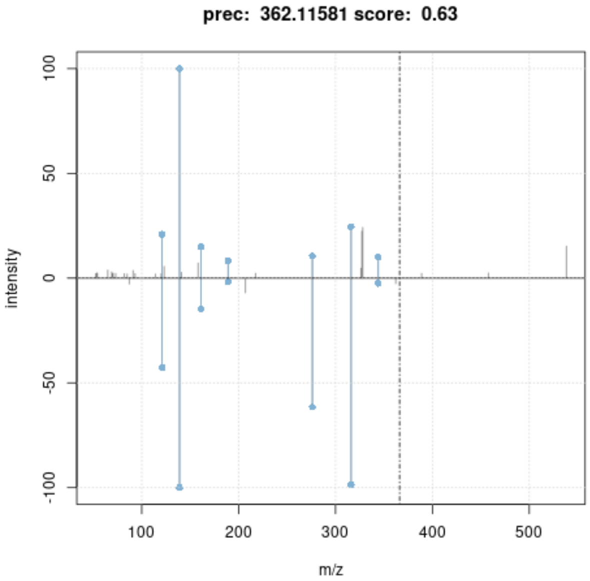

Figure S11: Spectra comparison between in-silico deconjugated spectra and reference spectra for bezafibrate.

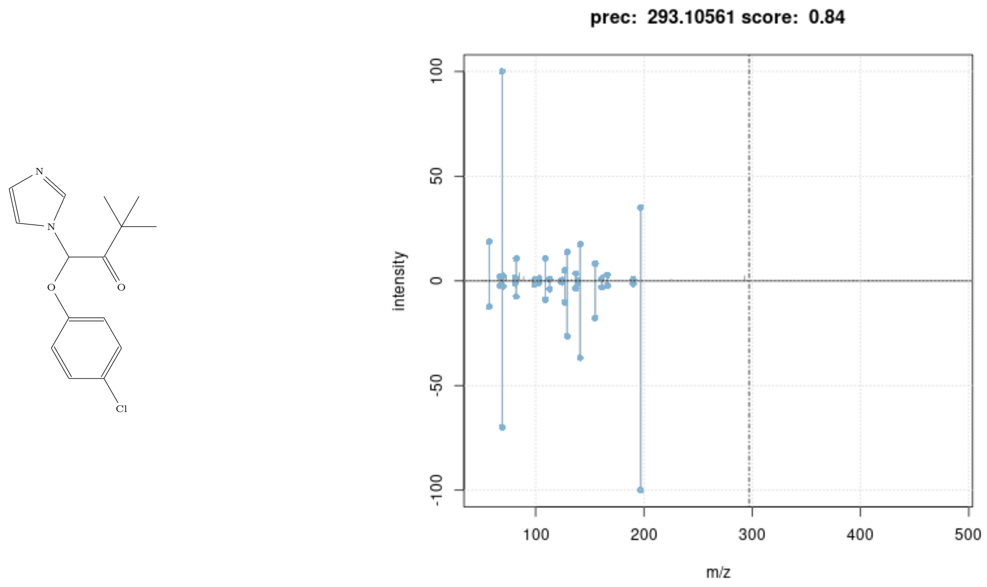

Figure S12: Spectra comparison between in-silico deconjugated spectra and reference spectra for climbazole.

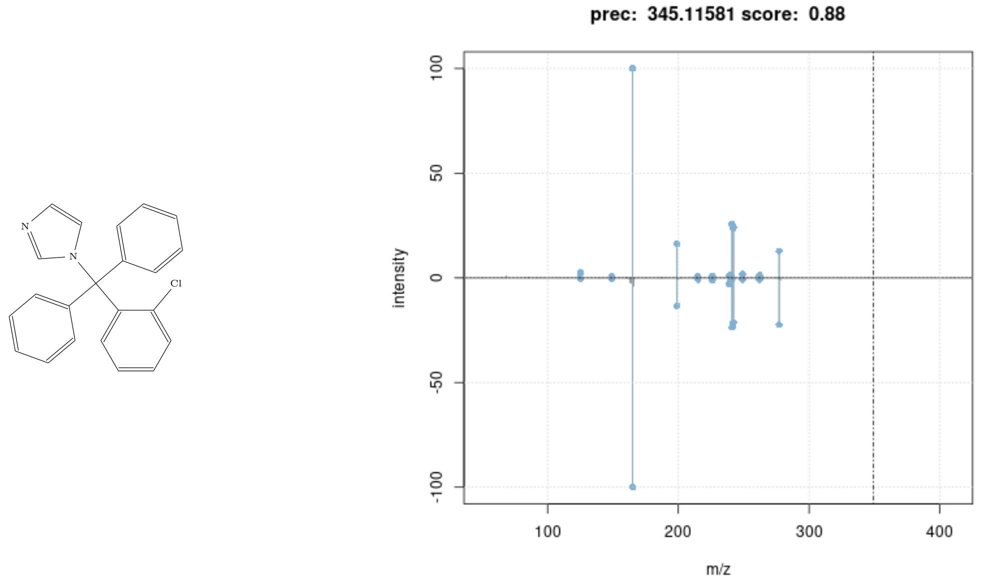

Figure S13: Spectra comparison between in-silico deconjugated spectra and reference spectra for clotrimazole.

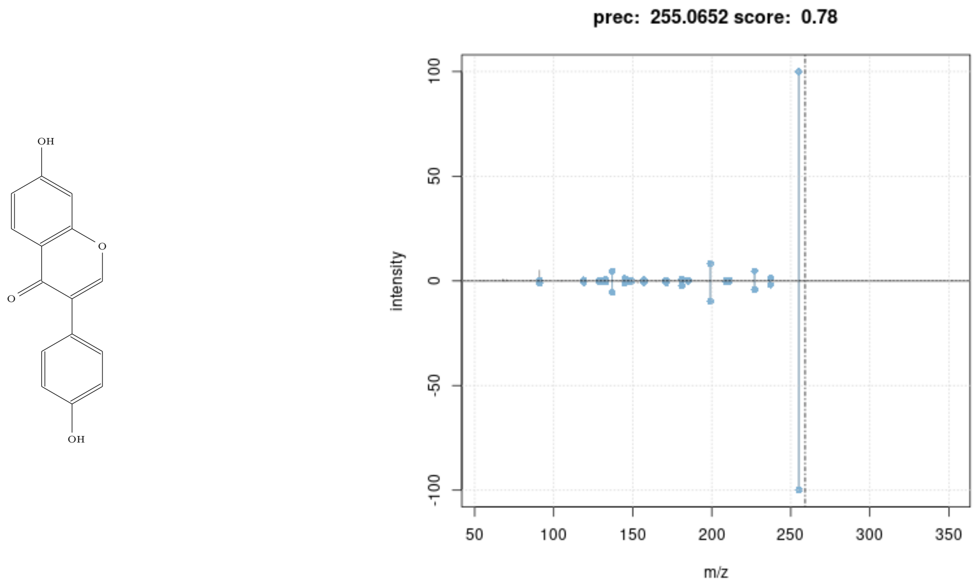

Figure S14: Spectra comparison between in-silico deconjugated spectra and reference spectra for daidzein.

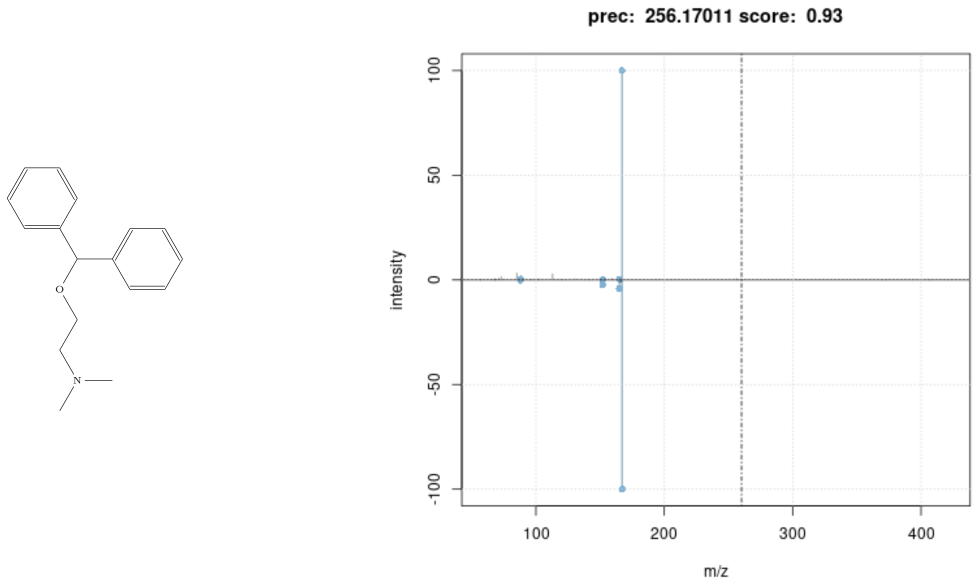

Figure S15: Spectra comparison between in-silico deconjugated spectra and reference spectra for diphenhydramine.

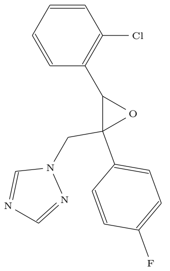

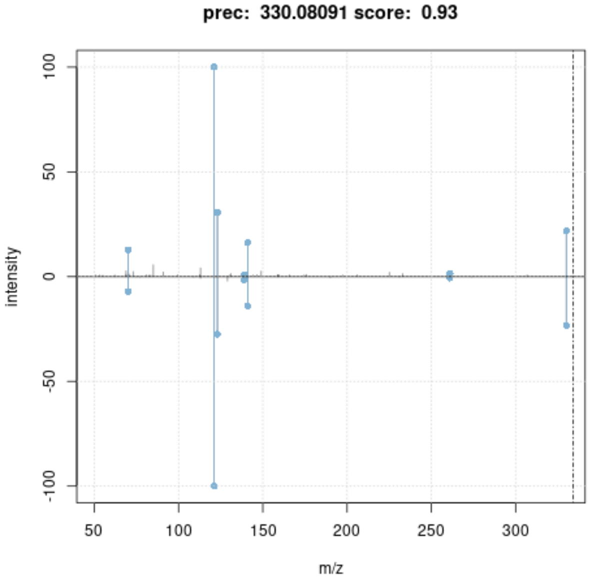

Figure S16: Spectra comparison between in-silico deconjugated spectra and reference spectra for epoxiconazole.

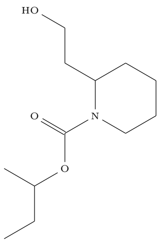

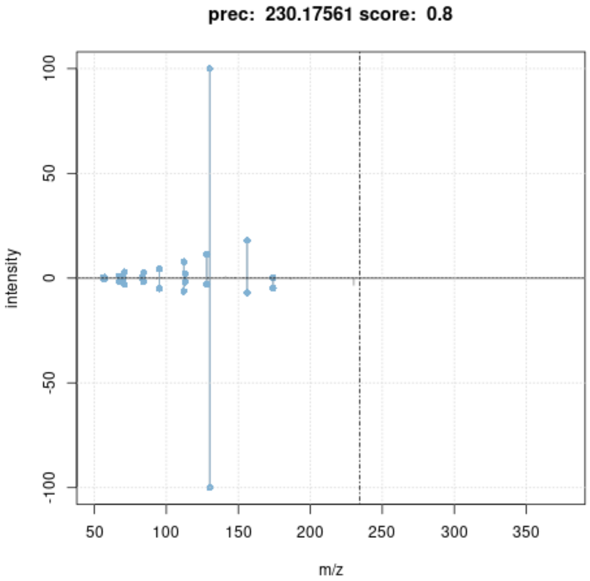

Figure S17: Spectra comparison between in-silico deconjugated spectra and reference spectra for icaridin.

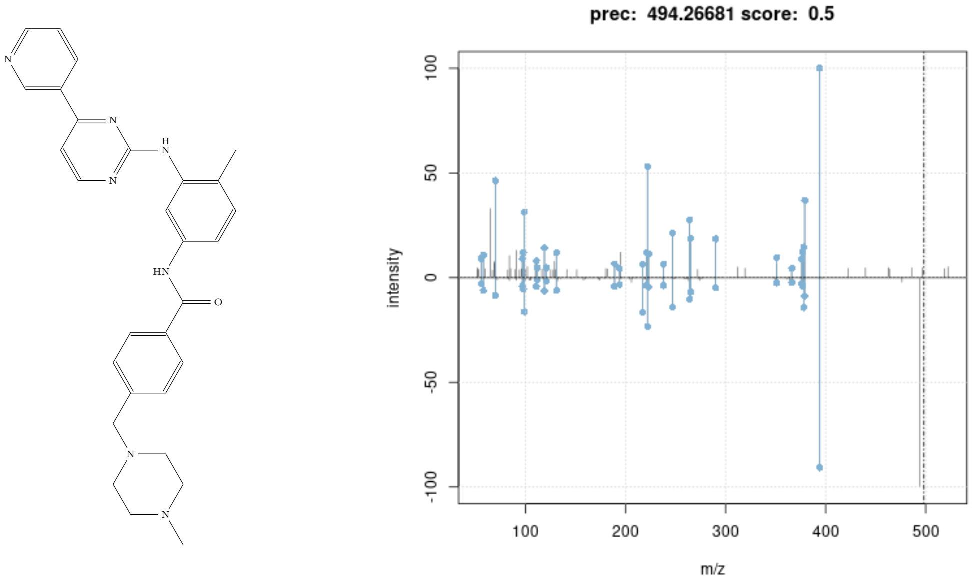

Figure S18: Spectra comparison between in-silico deconjugated spectra and reference spectra for imatinib.

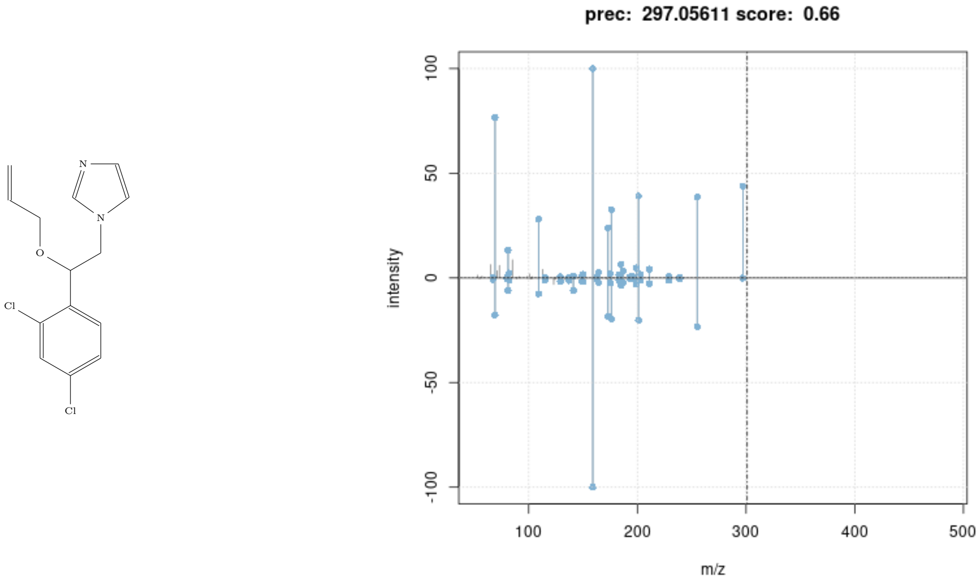

Figure S19: Spectra comparison between in-silico deconjugated spectra and reference spectra for imazalil.

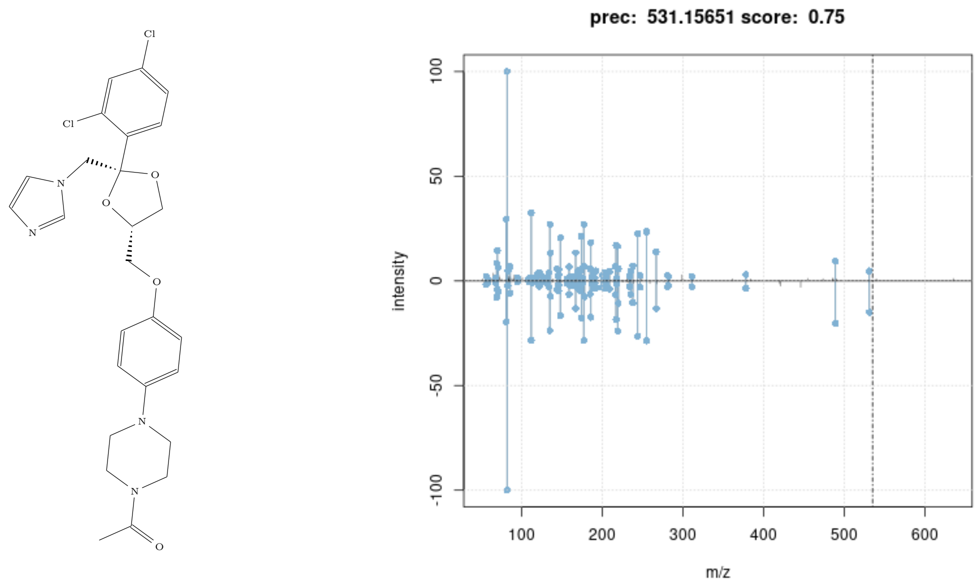

Figure S20: Spectra comparison between in-silico deconjugated spectra and reference spectra for ketoconazole.

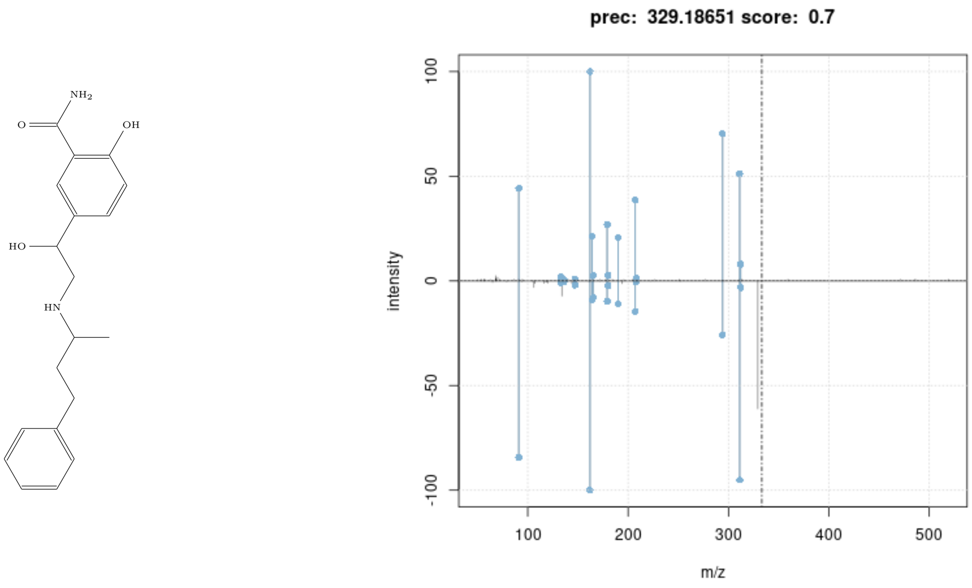

Figure S21: Spectra comparison between in-silico deconjugated spectra and reference spectra for labetalol.

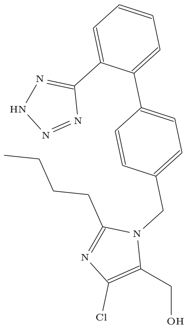

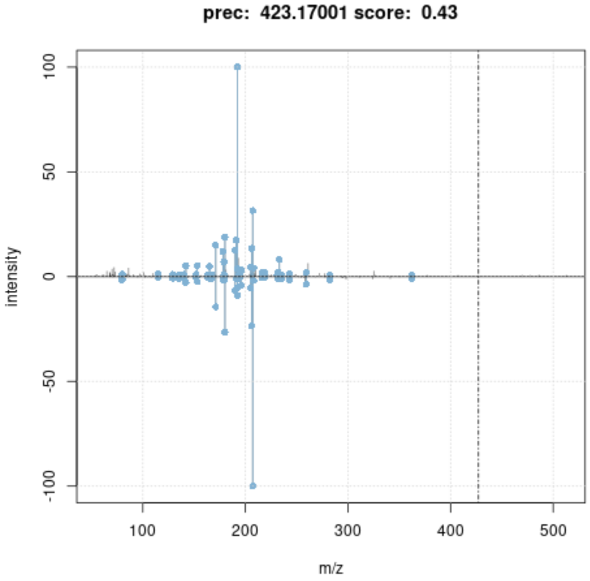

Figure S22: Spectra comparison between in-silico deconjugated spectra and reference spectra for losartan.

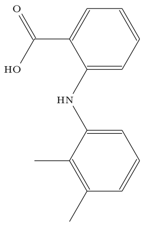

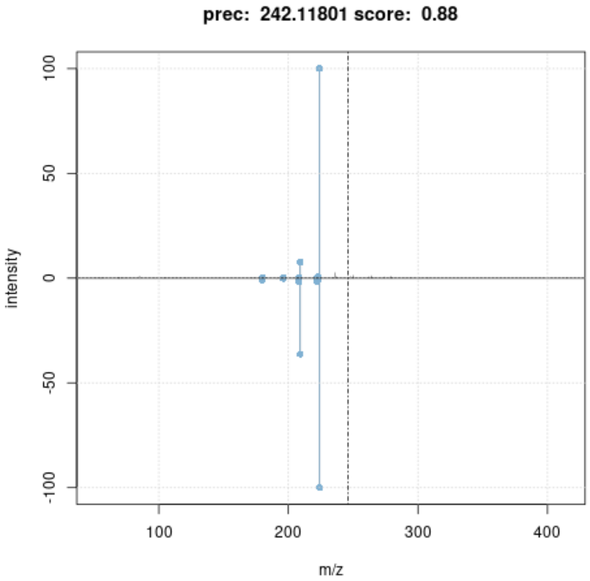

Figure S23: Spectra comparison between in-silico deconjugated spectra and reference spectra for mefenamic-acid.

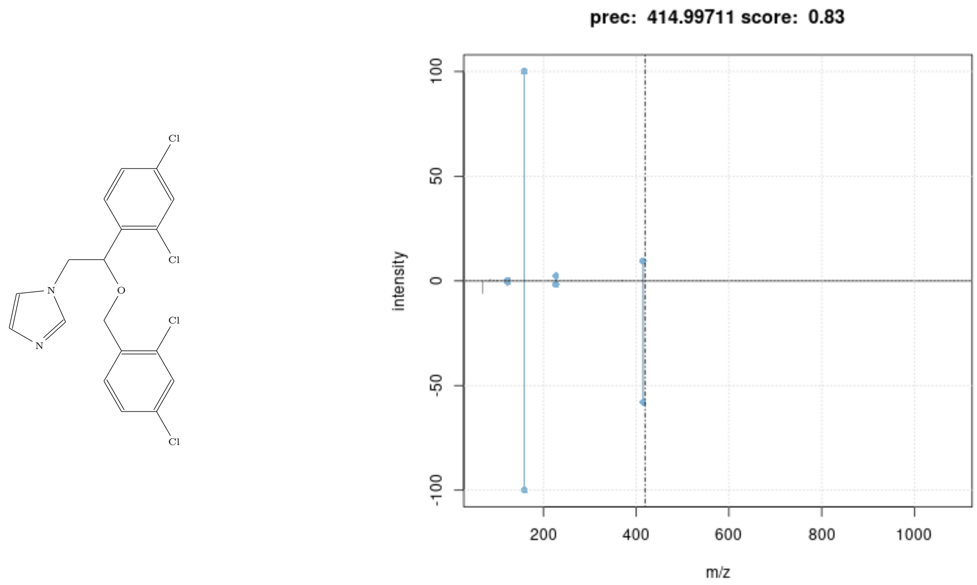

Figure S24: Spectra comparison between in-silico deconjugated spectra and reference spectra for miconazole.

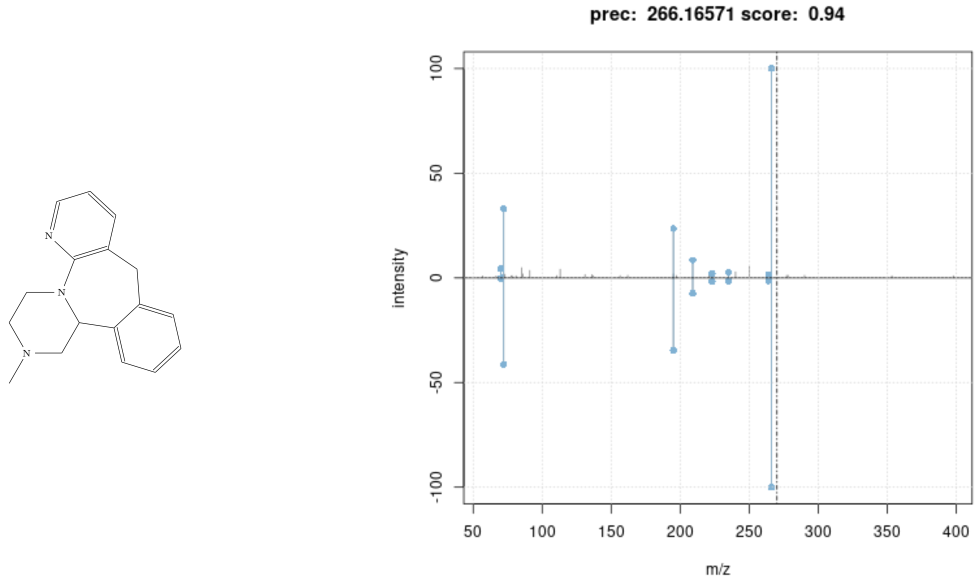

Figure S25: Spectra comparison between in-silico deconjugated spectra and reference spectra for mirtazapine.

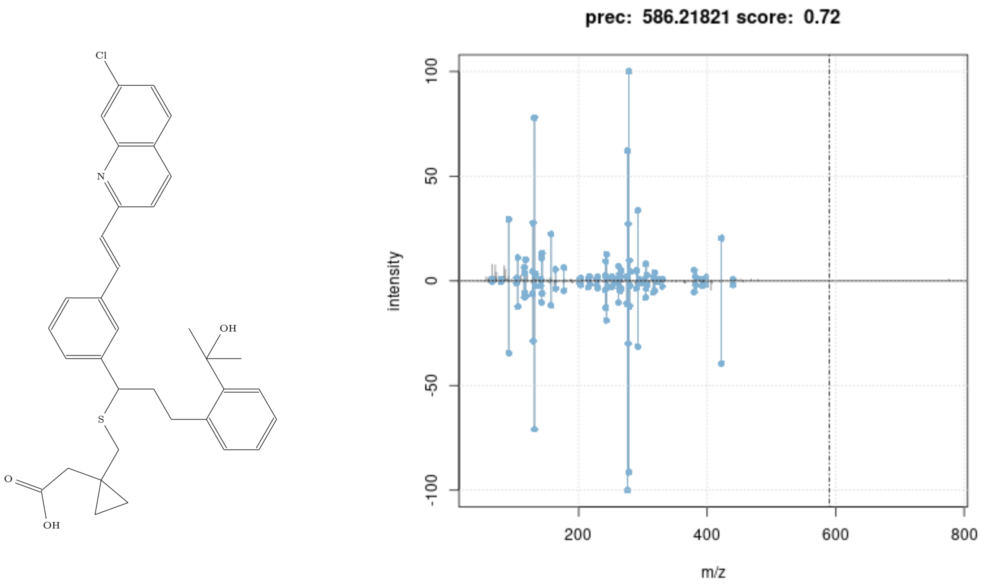

Figure S26: Spectra comparison between in-silico deconjugated spectra and reference spectra for montelukast.

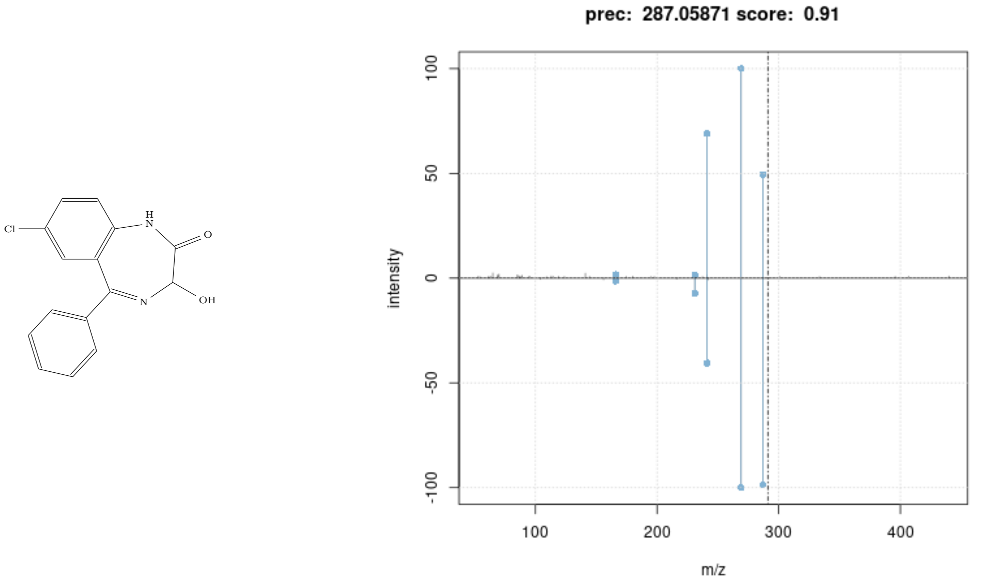

Figure S27: Spectra comparison between in-silico deconjugated spectra and reference spectra for oxazepam.

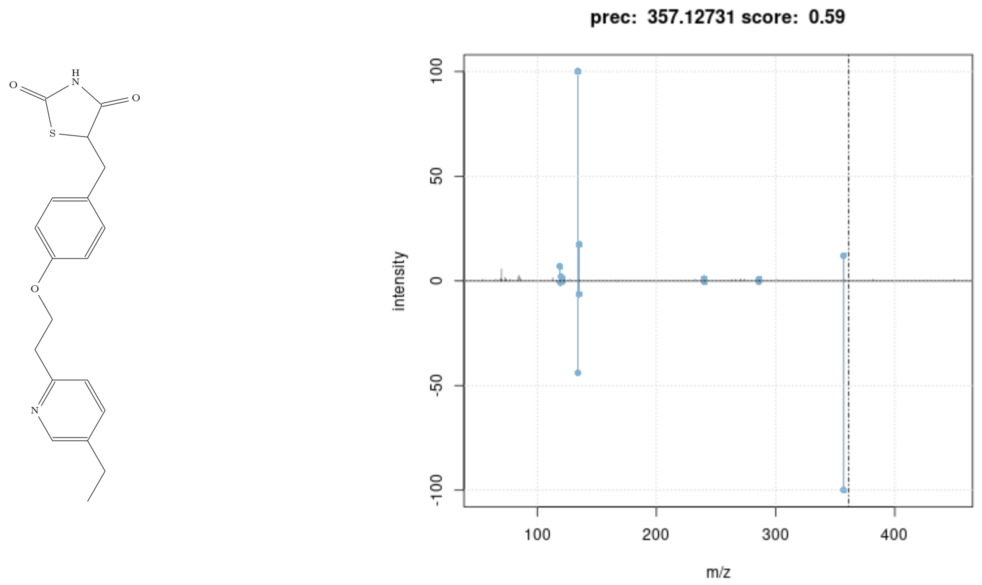

Figure S28: Spectra comparison between in-silico deconjugated spectra and reference spectra for pioglitazone.

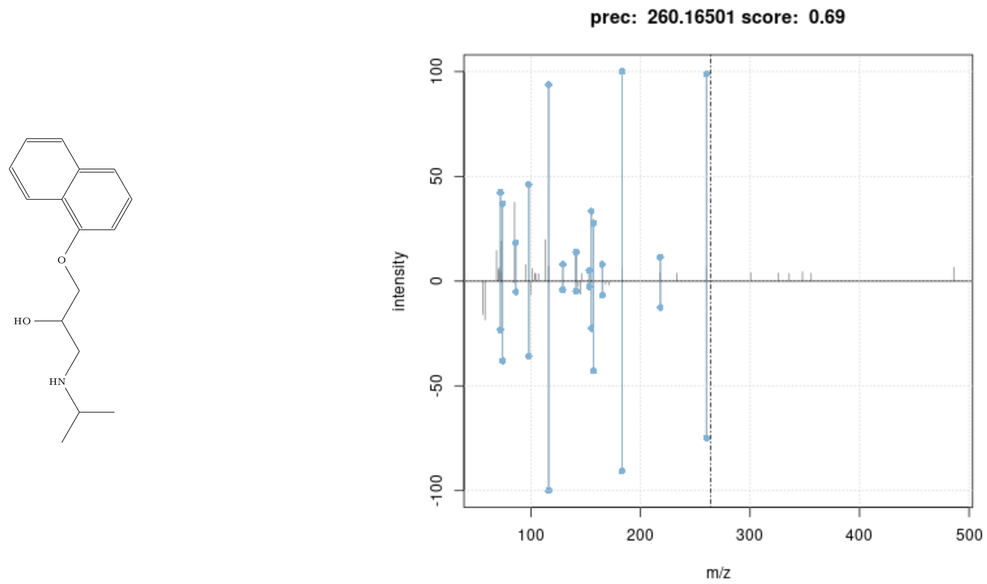

Figure S29: Spectra comparison between in-silico deconjugated spectra and reference spectra for propranolol.

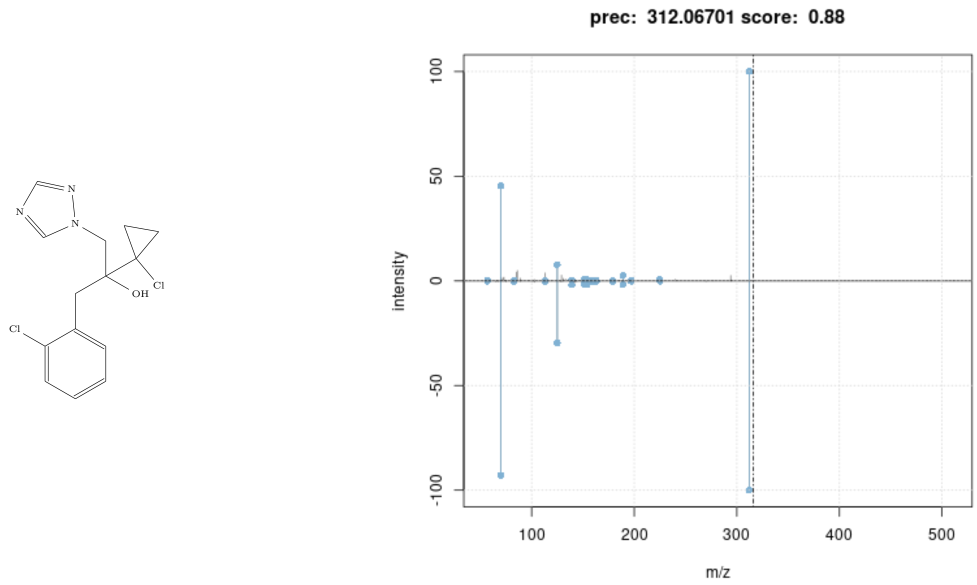

Figure S30: Spectra comparison between in-silico deconjugated spectra and reference spectra for prothioconazole-desthio.

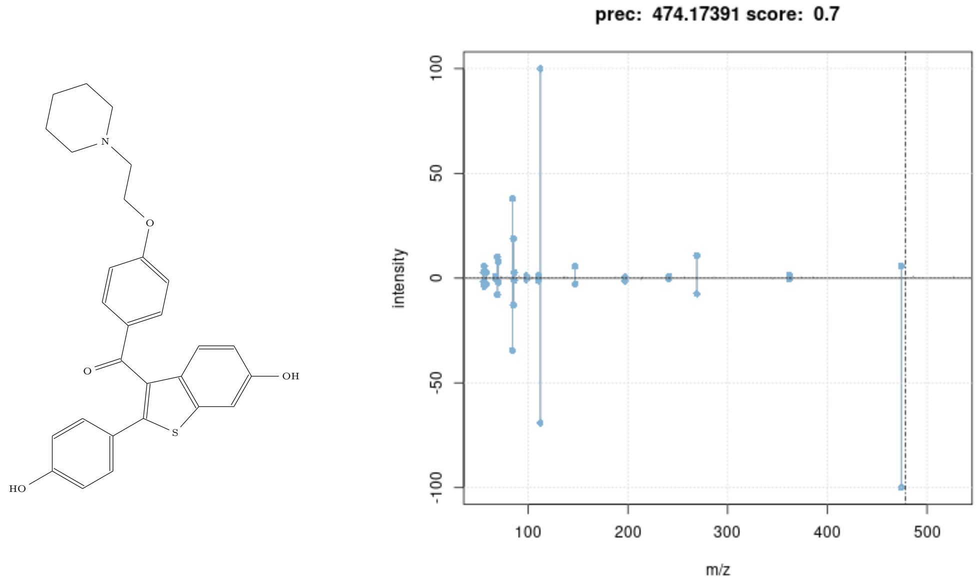

Figure S31: Spectra comparison between in-silico deconjugated spectra and reference spectra for raloxifene.

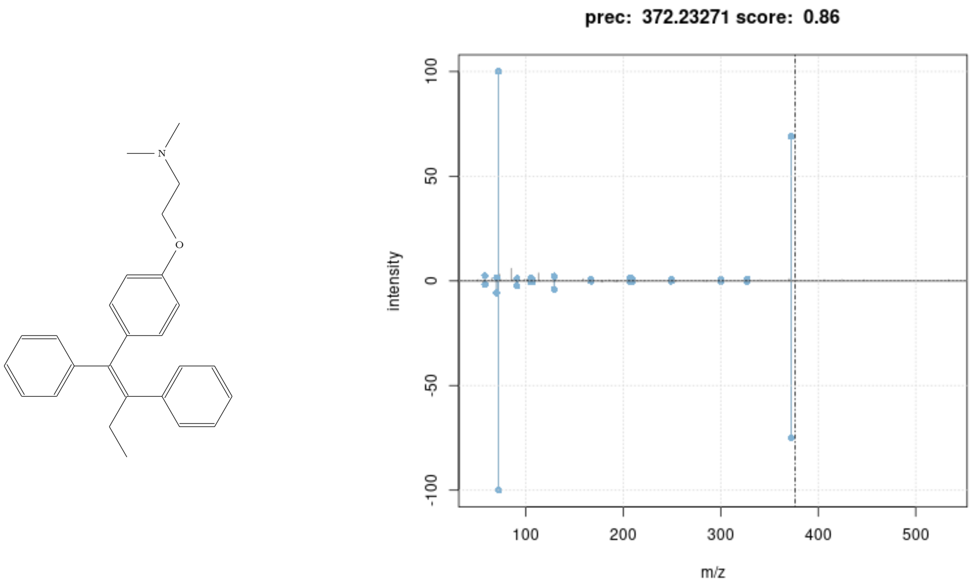

Figure S32: Spectra comparison between in-silico deconjugated spectra and reference spectra for tamoxifen.

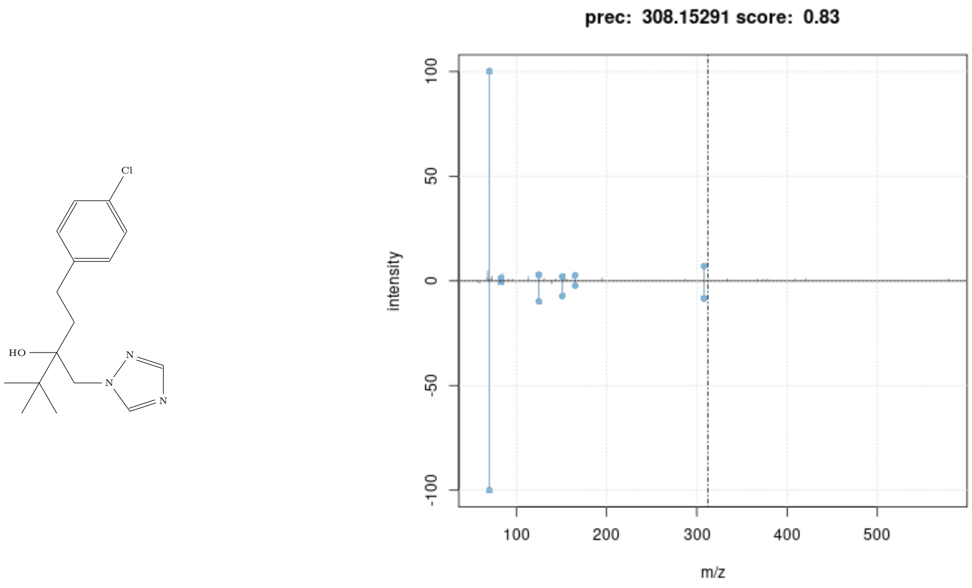

Figure S33: Spectra comparison between in-silico deconjugated spectra and reference spectra for tebuconazole.

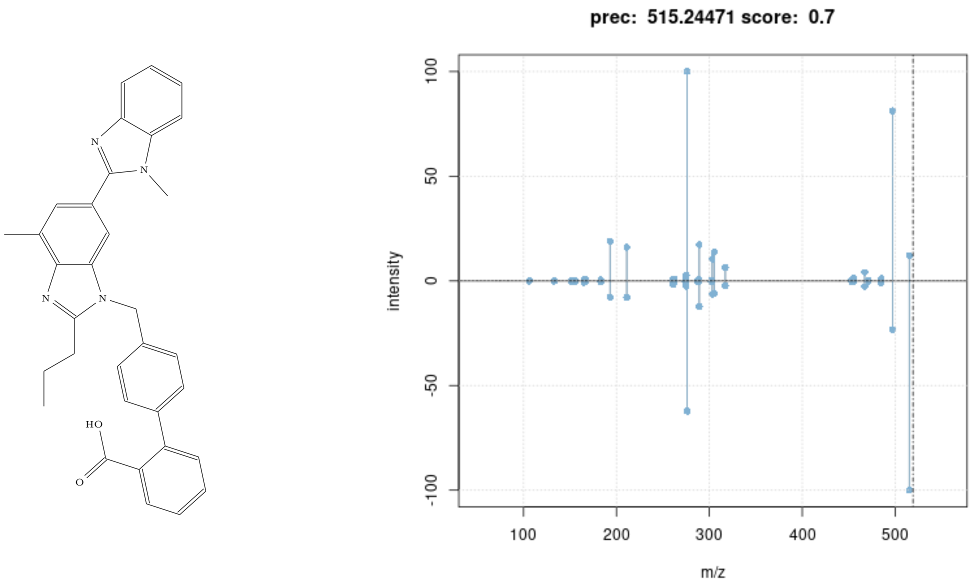

Figure S34: Spectra comparison between in-silico deconjugated spectra and reference spectra for telmisartan.

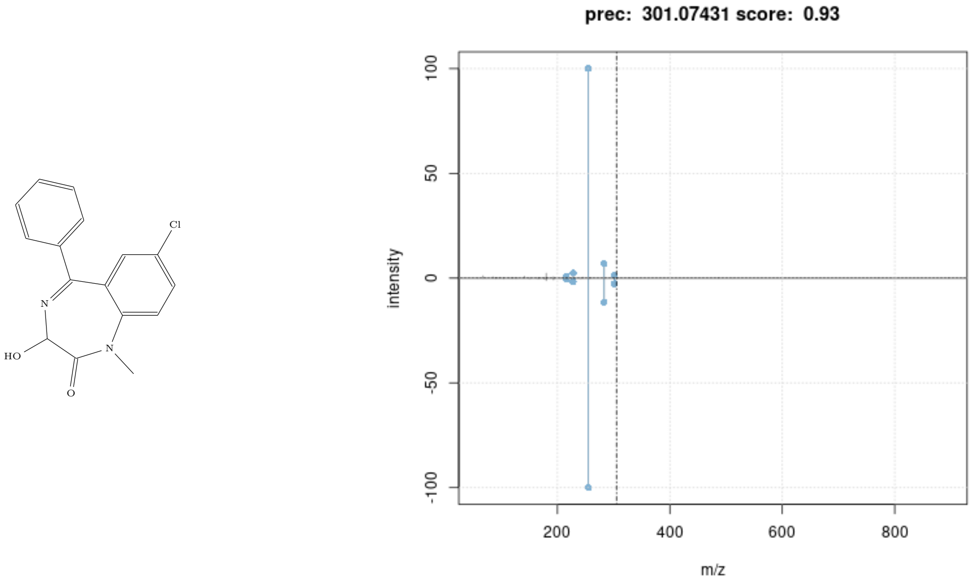

Figure S35: Spectra comparison between in-silico deconjugated spectra and reference spectra for temazepam.

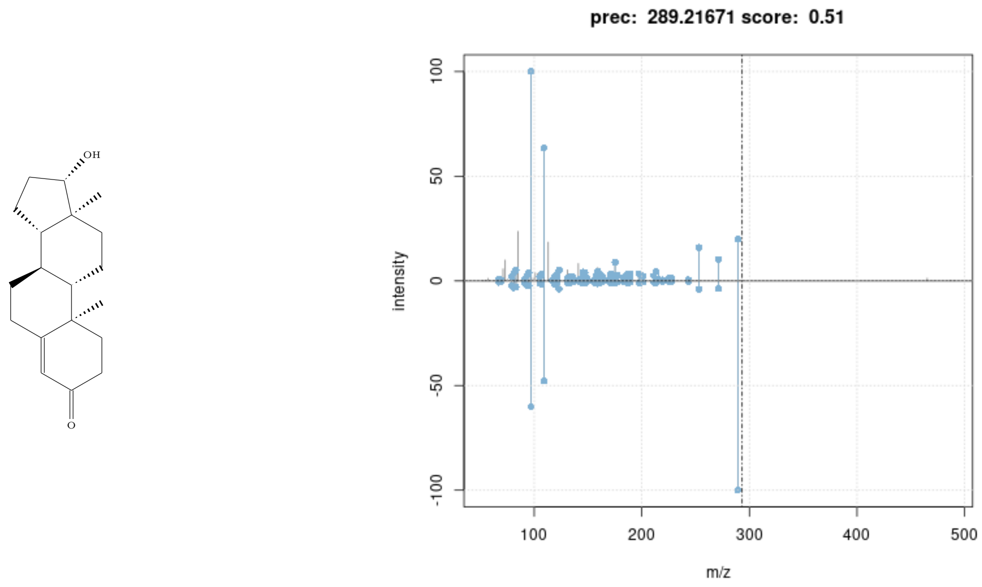

Figure S36: Spectra comparison between in-silico deconjugated spectra and reference spectra for testosterone.

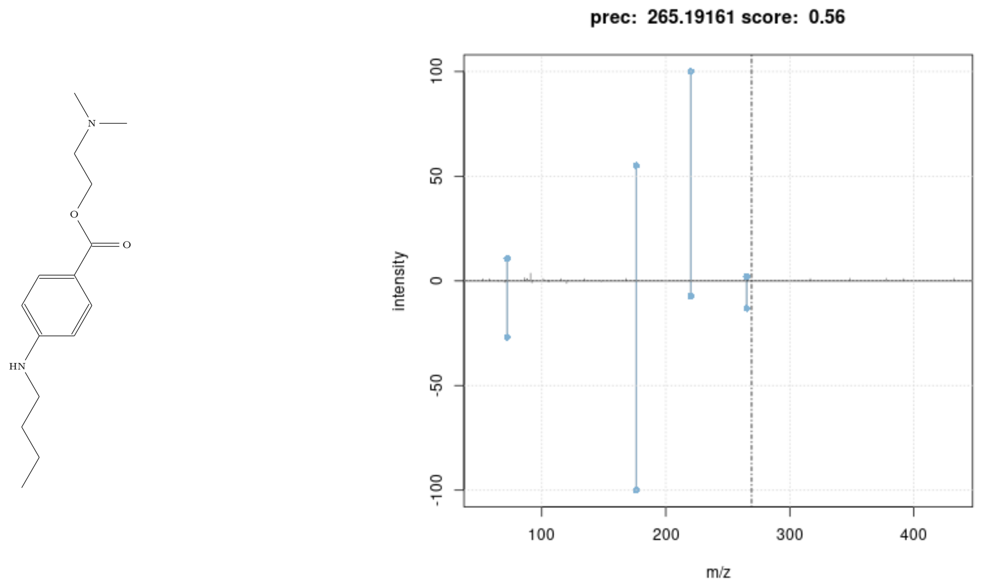

Figure S37: Spectra comparison between in-silico deconjugated spectra and reference spectra for tetracain.

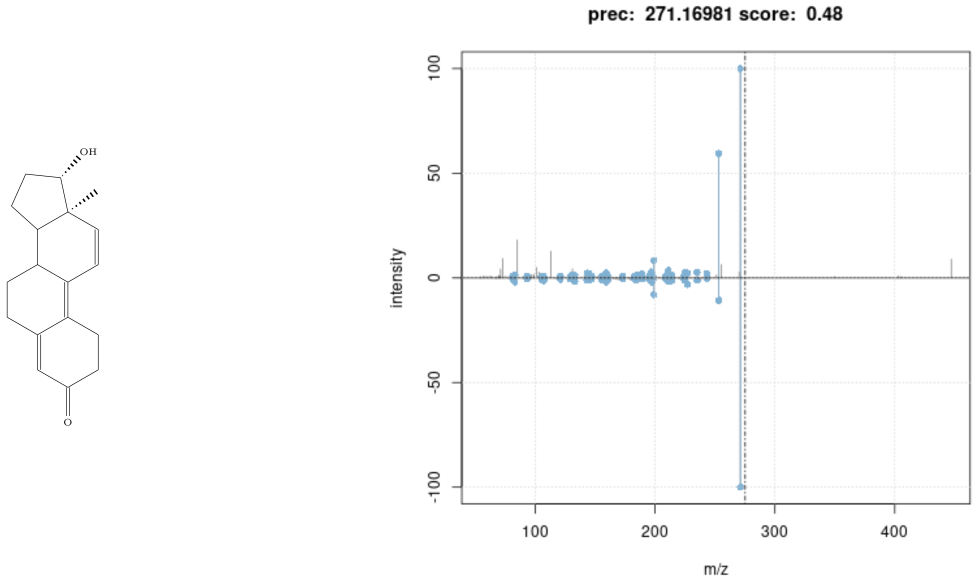

Figure S38: Spectra comparison between in-silico deconjugated spectra and reference spectra for trenbolone.

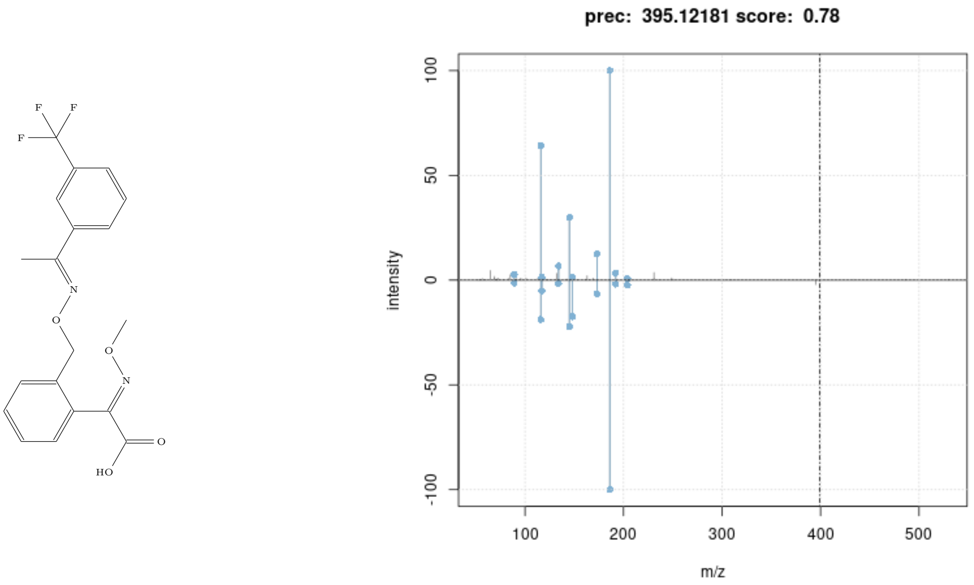

Figure S39: Spectra comparison between in-silico deconjugated spectra and reference spectra for trifloxystrobin-cga-321113.

S4 Descriptive statistics table of found glucuronides in urine data set

Table S2: Descriptive statistics of parent and conjugated drugs, including detection rate (DR), theoretical precursor mz (Prec m/z) and the average match probability of the spectral library search algorithm.

**Compound**	**DR**	**Signal Intensity**			**Prec**	**RT**	**AMP**		
	MS1/MS2	Min	Median	Max	m/z	[min]	Min	Median	Max
Ambroxol	1/1	2.93E+01	2.93E+01	2.93E+01	376.98641	5.5	20	20	20
Ambroxol glucuronide[25]	1/1	2.03E+02	2.03E+02	2.03E+02	553.01852	4.9	19	19	19
Amytriptyline	4/3	4.65E+03	4.65E+03	4.65E+03	278.19087	8.3	43	46	47
Amytriptyline glucuronide[2]	4/4	4.28E+03	1.37E+04	4.14E+04	454.22298	6.7	31	33	34
Cinnarizine	1/1	1.08E+02	1.08E+02	1.08E+02	369.23307	11.2	39	39	39
Cinnarizine glucuronide[26]	1/1	3.68E+01	3.68E+01	3.68E+01	545.26518	8.6	18	18	18
Citalopram	3/3	2.19E+03	5.23E+03	8.18E+03	325.17162	7.1	31	33	33
Citalopram glucuronide[27]	3/3	4.69E+02	2.37E+03	4.64E+03	501.20372	6.0	23	28	30
Cyclobenzaprine	0/1	0.00E+00	0.00E+00	0.00E+00	276.17522	-	45	45	45
Cyclobenzaprine glucuronide[22]	1/1	4.22E+01	4.22E+01	4.22E+01	452.20733	6.6	16	16	16
Demoxepam	1/1	1.53E+02	1.53E+02	1.53E+02	287.05873	6.9	40	40	40
Demoxepam glucuronide[28]	1/1	1.34E+02	1.34E+02	1.34E+02	463.09084	5.4	33	33	33
Desmethyldihydrocodeine	1/1	3.46E+03	3.46E+03	3.46E+03	288.15997	3.5	43	43	43
Desmethyldihydrocodeine glucuronide[29]	1/1	2.70E+02	2.70E+02	2.70E+02	464.19207	3.2	27	27	27
Hydroquinone	1/1	1.91E+03	1.91E+03	1.91E+03	327.20725	5.5	39	39	39
Hydroquinone glucuronide[30]	1/1	2.69E+01	2.69E+01	2.69E+01	503.23936	4.8	12	12	12
Melitracene	1/1	5.18E+02	5.18E+02	5.18E+02	292.20652	9.1	33	33	33
Melitracene glucuronide	1/1	5.00E+03	5.00E+03	5.00E+03	468.23863	7.2	36	36	36
Norcodeine	2/1	8.66E+02	9.13E+02	9.59E+02	286.14432	3.5	7	7	7
Norcodeine glucuronide[29]	2/2	6.24E+02	8.42E+02	1.06E+03	462.17642	3.2	24	25	25
Prednisolone	1/1	3.54E+02	3.54E+02	3.54E+02	361.20150	6.4	24	24	24
Prednisolone glucuronide[31]	1/1	1.38E+02	1.38E+02	1.38E+02	537.23360	5.7	19	19	19
Pheniramine	1/1	1.37E+03	1.37E+03	1.37E+03	241.17047	4.8	45	45	45
Pheniramine glucuronide[32]	1/1	1.33E+03	1.33E+03	1.33E+03	417.20258	3.8	45	45	45
Propafenone	1/1	2.86E+03	2.86E+03	2.86E+03	342.20692	8.2	38	38	38
Propafenone glucuronide[33]	1/1	4.22E+03	4.22E+03	4.22E+03	518.23902	6.8	12	12	12
Quinapril	4/4	7.75E+00	4.11E+02	7.68E+02	439.22330	8.2	35	42	45
Quinapril glucuronide	4/3	1.19E+01	3.94E+02	8.84E+02	615.25540	7.1	14	20	25
Quinidine	2/2	1.64E+01	1.79E+03	3.56E+03	325.19160	5.0	11	27	35
Quinidine glucuronide[34]	1/1	7.61E+01	7.61E+01	7.61E+01	501.22371	4.3	28	28	28
Solifenacin	1/1	2.20E+03	2.20E+03	2.20E+03	363.20725	8.4	48	48	48
Solifenacin glucuronide[22]	1/1	3.24E+02	3.24E+02	3.24E+02	539.23936	7.0	12	12	12
Temazepam	0/1	0.00E+00	0.00E+00	0.00E+00	301.07438	-	21	21	21
Temazepam glucuronide[22]	1/1	4.79E+02	4.79E+02	4.79E+02	477.10649	6.5	43	43	43
O-desmethyltramadol	6/6	8.24E+02	1.71E+03	3.11E+03	250.18070	4.0	15	45	73
O-desmethyltramadol glucuronide[35]	6/6	2.04E+03	7.48E+03	1.40E+04	426.21281	3.4	23	34	45
Warfarin	2/2	1.53E+01	2.00E+01	2.47E+01	309.11268	6.7	21	27	30
Warfarin glucuronide[36]	2/1	8.86E+00	1.66E+01	2.44E+01	485.14479	6.1	14	14	14

Table S3: Descriptive statistics of the prescribed parent and conjugated drugs, including detection rate (DR), theoretical precursor mz (Prec m/z) and the average match probability of the spectral library search algorithm.

**Compound**	**DR**	**Signal Intensity**			**Prec**	**RT**	**AMP**		
	MS1/MS2	Min	Median	Max	m/z	[min]	Min	Median	Max
Betaxolol	4/4	3.88E+02	2.70E+03	6.83E+03	308.22257	6.6	21	28	30
Betaxolol glucuronide[37]	3/3	6.67E+02	1.74E+03	2.05E+03	484.25467	5.9	15	16	17
Bisoprolol	8/7	1.00E+03	3.44E+03	5.37E+03	326.23313	5.9	24	27	28
Bisoprolol glucuronide[38]	8/7	9.15E+01	3.27E+02	6.77E+02	502.26524	5.3	12	14	15
Carvedilol	6/6	6.06E+01	8.23E+01	3.96E+02	407.19708	8.3	27	33	38
Carvedilol glucuronide[22]	6/6	5.40E+01	3.40E+02	1.50E+03	583.22919	6.9	16	19	22
Diltiazem	1/1	8.43E+03	8.43E+03	8.43E+03	415.16915	7.6	24	24	24
Diltiazem glucuronide[39]	1/1	1.59E+03	1.59E+03	1.59E+03	591.20126	6.3	27	27	27
Doxazosin	11/11	1.41E+01	1.83E+02	4.08E+02	452.19339	7.2	10	33	42
Doxazosin glucuronide	7/2	9.68E+00	1.75E+01	3.58E+01	628.22550	5.6	9	14	16
Gliclazide	12/12	6.40E+00	4.80E+00	2.00E+02	324.13819	8.8	37	44	48
Gliclazide glucuronide[40]	3/3	1.90E+00	2.00E+01	2.50E+01	500.17029	4.9	8	27	39
Indapamide	9/9	1.52E+01	6.48E+01	1.09E+02	366.06791	7.4	14	24	30
Indapamide glucuronide[41]	9/6	1.20E+01	2.02E+01	3.69E+01	542.10002	5.9	5	7	9
Perindopril	2/2	3.64E+02	8.35E+02	1.31E+03	369.23895	6.5	31	33	33
Perindopril glucuronide[22]	4/4	1.13E+02	1.41E+03	6.61E+03	545.27105	5.5	24	28	29
Losartan	3/3	2.07E+02	2.31E+02	5.76E+03	423.17001	7.9	35	37	38
Losartan glucuronide[10]	3/3	6.09E+01	2.60E+02	3.88E+03	599.20212	7.3	10	21	26
Metoprolol	9/9	8.38E+01	2.82E+03	4.66E+03	268.19127	5.0	17	22	31
Metoprolol glucuronide[42]	9/9	2.13E+01	1.34E+03	1.93E+03	444.22337	4.5	9	13	17
Rilmenidine	4/4	7.47E+01	4.44E+02	5.83E+02	181.13409	4.5	27	32	37
Rilmenidine glucuronide	4/4	1.60E+01	1.49E+02	2.46E+02	357.16619	4.1	6	10	12
Rosuvastatin	10/10	3.29E+01	1.17E+03	7.75E+03	482.17611	8.0	27	32	36
Rosuvastatin glucuronide[43]	9/4	4.19E+00	3.19E+01	1.03E+02	658.20821	6.9	5	15	20
Sotalol	1/1	8.19E+03	8.19E+03	8.19E+03	273.12729	3.1	43	43	43
Sotalol glucuronide[33]	1/1	3.09E+02	3.09E+02	3.09E+02	449.15939	3.0	31	31	31
Telmisartan	2/2	6.54E+01	9.96E+01	1.34E+02	515.24470	9.7	50	54	56
Telmisartan glucuronide[22]	2/1	9.18E+02	1.11E+03	1.31E+03	691.27681	7.5	48	48	48
Vildagliptine	4/4	4.02E+01	3.59E+02	4.45E+03	304.20250	3.5	24	41	52
Vildagliptine glucuronide[44]	3/2	1.02E+01	8.60E+01	9.13E+02	480.23461	3.2	25	27	28
Irbesartan	5/5	1.50E+01	3.40E+02	6.27E+02	429.24028	8.5	15	43	52
Irbesartan glucuronide[22]	5/4	8.96E+01	3.41E+03	9.21E+03	605.27239	7.5	15	18	20
Cilazapril	1/1	8.98E+02	8.98E+02	8.98E+02	418.23420	7.5	21	21	21
Cilazapril glucuronide[45]	1/1	3.36E+02	3.36E+02	3.36E+02	594.26630	6.4	10	10	10
Atenolol	2/2	4.18E+03	4.67E+03	5.17E+03	267.17087	3.0	36	36	36
Atenolol glucuronide[46]	2/2	4.62E+01	2.76E+02	5.06E+02	443.20297	3.0	21	30	34
Ramipril	5/4	8.57E+00	1.15E+02	7.73E+02	417.23895	7.5	30	38	41
Ramipril glucuronide[22]	4/4	1.74E+01	2.07E+02	1.61E+03	593.27105	6.3	12	27	34
Nebivolol	5/3	4.24E+00	1.92E+01	3.22E+02	406.18299	8.3	5	21	29
Nebivolol glucuronide[47]	7/6	1.70E+01	1.78E+02	3.18E+03	582.21509	7.2	8	12	16
Codeine	2/2	3.98E+03	4.05E+03	4.12E+03	300.15997	3.8	39	39	39
Codeine glucuronide[29]	2/2	4.04E+04	4.25E+04	4.46E+04	476.19207	3.5	27	28	28
Diclofenac	2/2	5.99E+00	8.97E+00	1.20E+01	296.02451	9.5	32	36	40
Diclofenac glucuronide[22]	2/2	3.05E+01	7.60E+01	1.22E+02	472.05661	7.6	24	25	25
Dihydrocodeine	1/1	1.54E+04	1.54E+04	1.54E+04	302.17562	3.7	43	43	43
Dihydrocodeine glucuronide[48]	1/1	1.71E+04	1.71E+04	1.71E+04	478.20772	3.5	31	31	31
Dihydromorphine	2/2	5.21E+01	1.76E+03	3.46E+03	288.15997	3.5	18	29	35
Dihydromorphine glucuronide[49]	2/2	8.76E+02	1.58E+03	2.28E+03	464.19207	6.3	8	11	12
Morphine	2/2	8.66E+02	9.13E+02	9.59E+02	286.14432	3.5	8	17	22
Morphine glucuronide[22]	2/2	6.24E+02	8.42E+02	1.06E+03	462.17642	3.2	7	19	29
Quetiapine	1/1	7.94E+01	7.94E+01	7.94E+01	384.17457	7.2	45	45	45
Quetiapine glucuronide[22]	1/1	1.30E+03	1.30E+03	1.30E+03	560.20668	6.1	48	48	48

S5 Head-tailplots of detected glucuronidated pharmaceuticals in urine data set

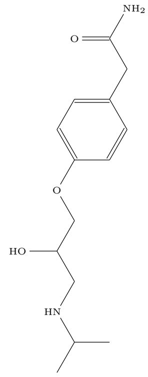

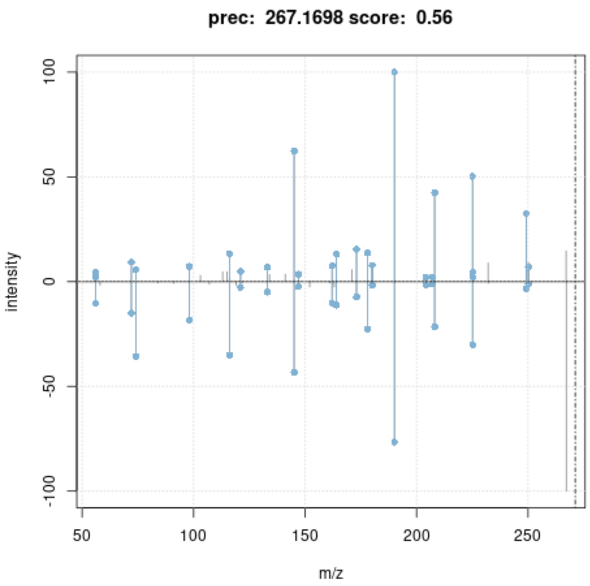

Figure S40: Spectra comparison between in-silico deconjugated spectra and reference spectra for atenolol.

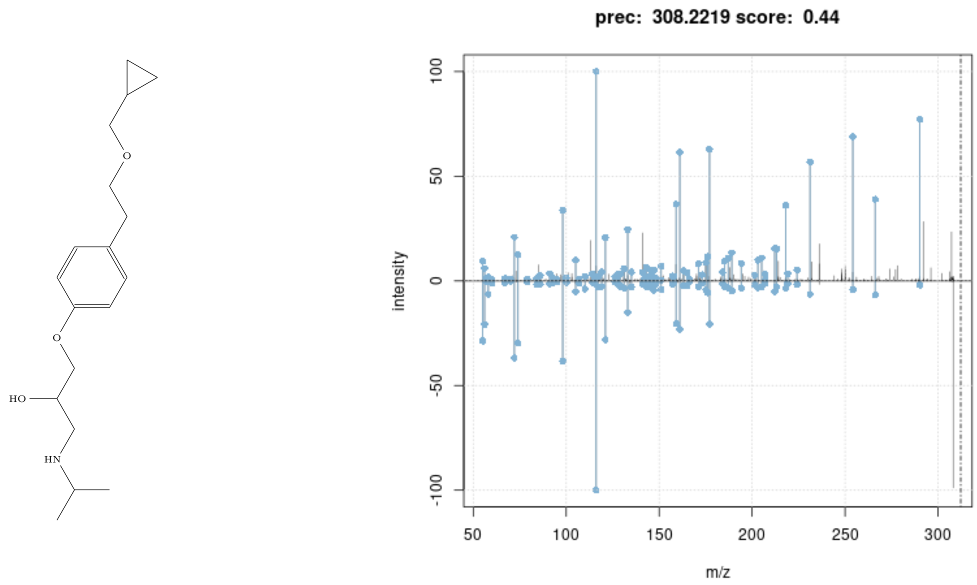

Figure S41: Spectra comparison between in-silico deconjugated spectra and reference spectra for betaxolol.

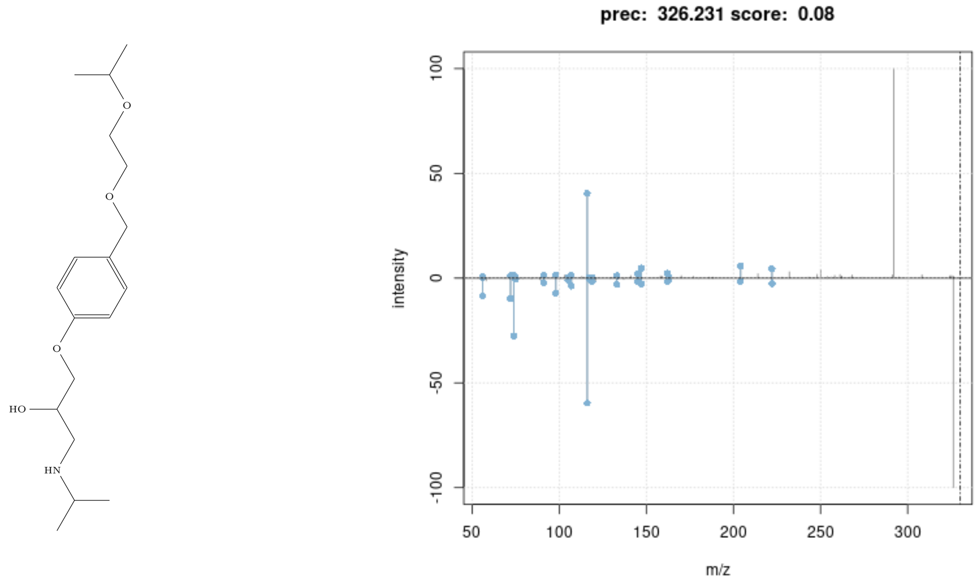

Figure S42: Spectra comparison between in-silico deconjugated spectra and reference spectra for bisoprolol.

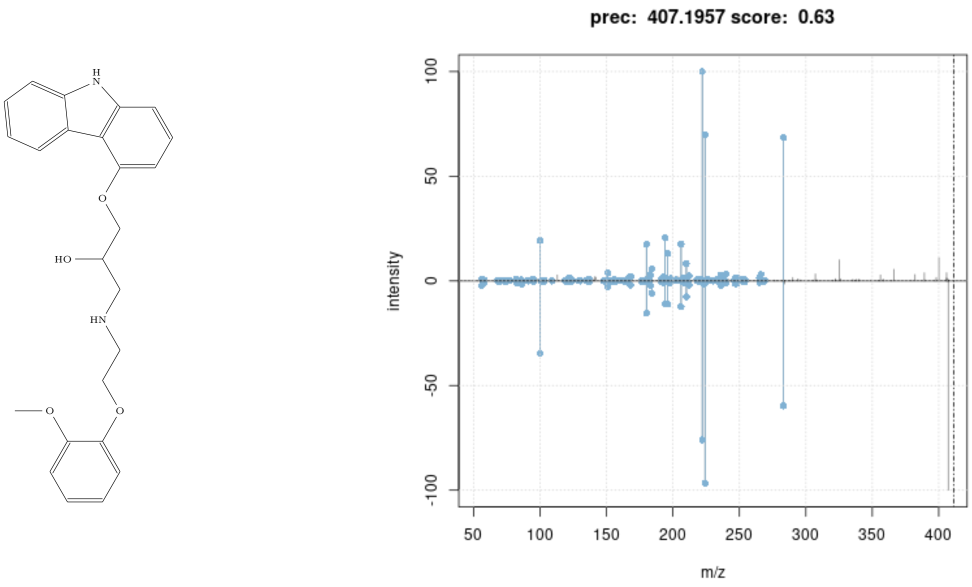

Figure S43: Spectra comparison between in-silico deconjugated spectra and reference spectra for carvedilol.

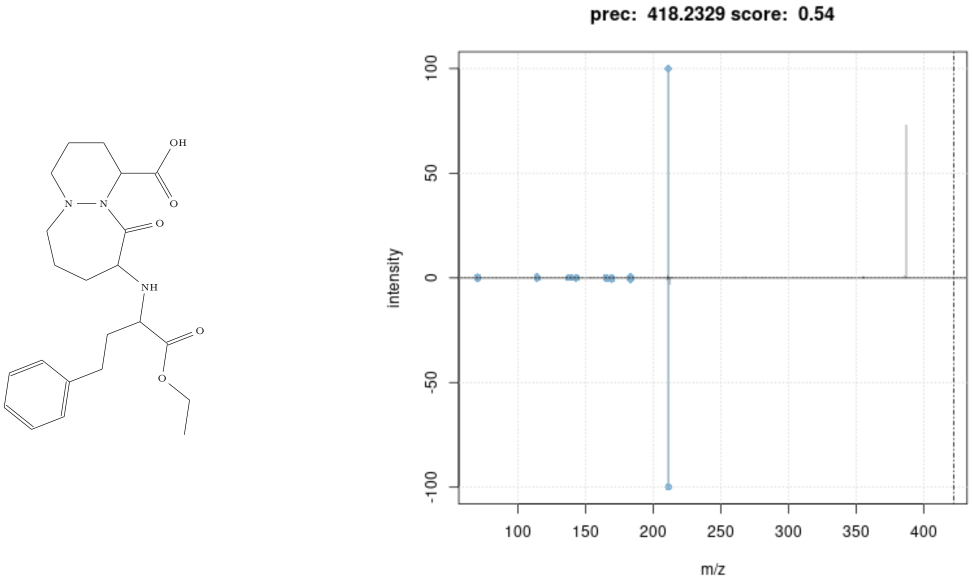

Figure S44: Spectra comparison between in-silico deconjugated spectra and reference spectra for cilazapril.

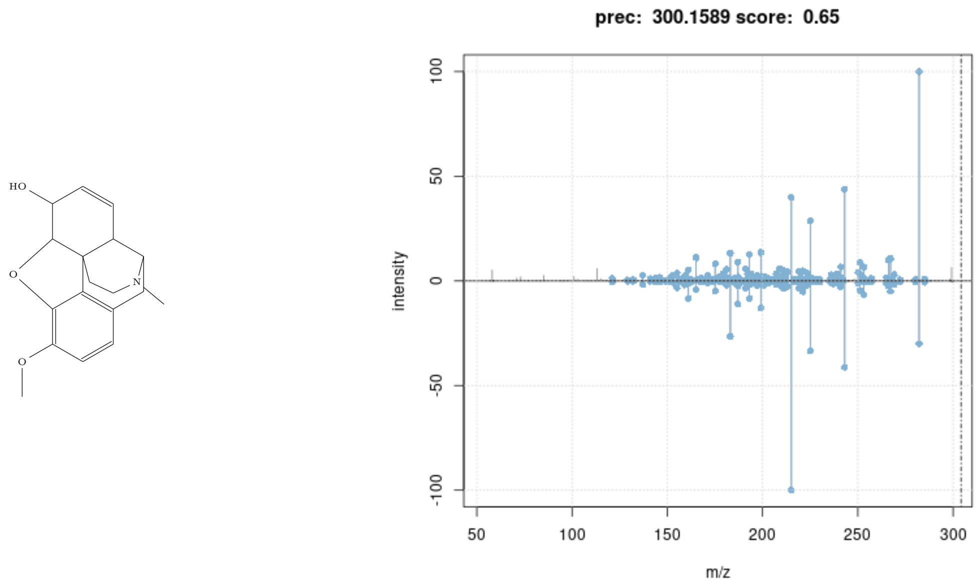

Figure S45: Spectra comparison between in-silico deconjugated spectra and reference spectra for codeine.

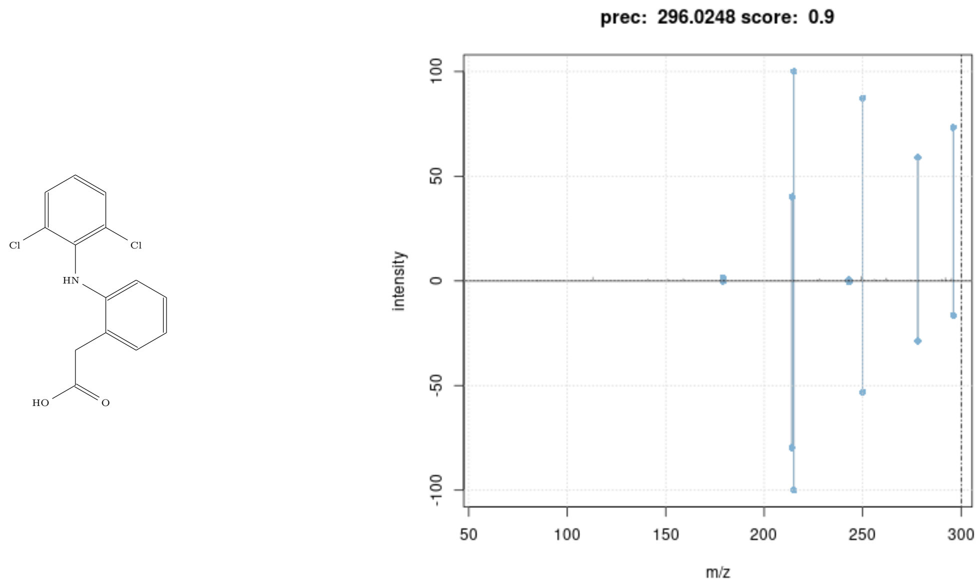

Figure S46: Spectra comparison between in-silico deconjugated spectra and reference spectra for diclofenac.

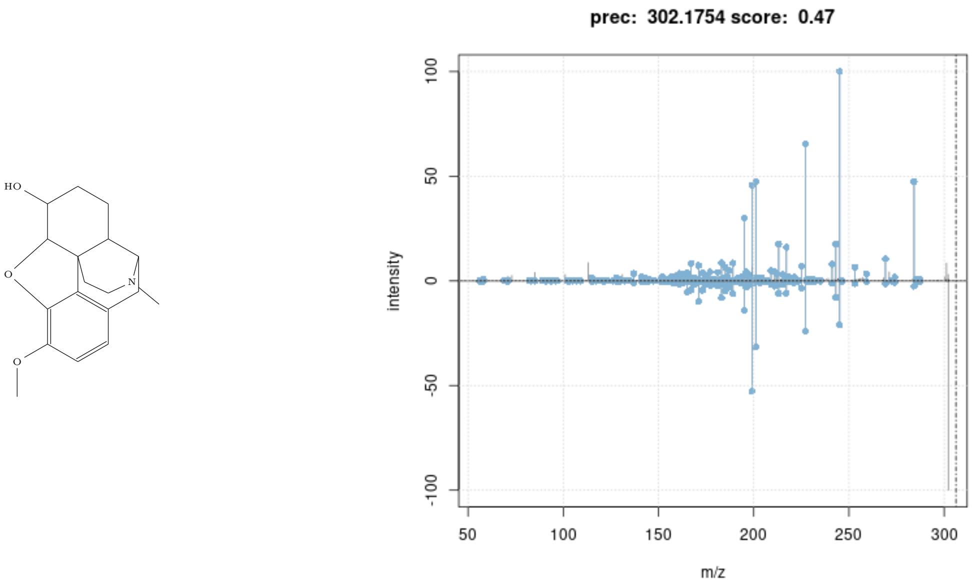

Figure S47: Spectra comparison between in-silico deconjugated spectra and reference spectra for dihydrocodeine.

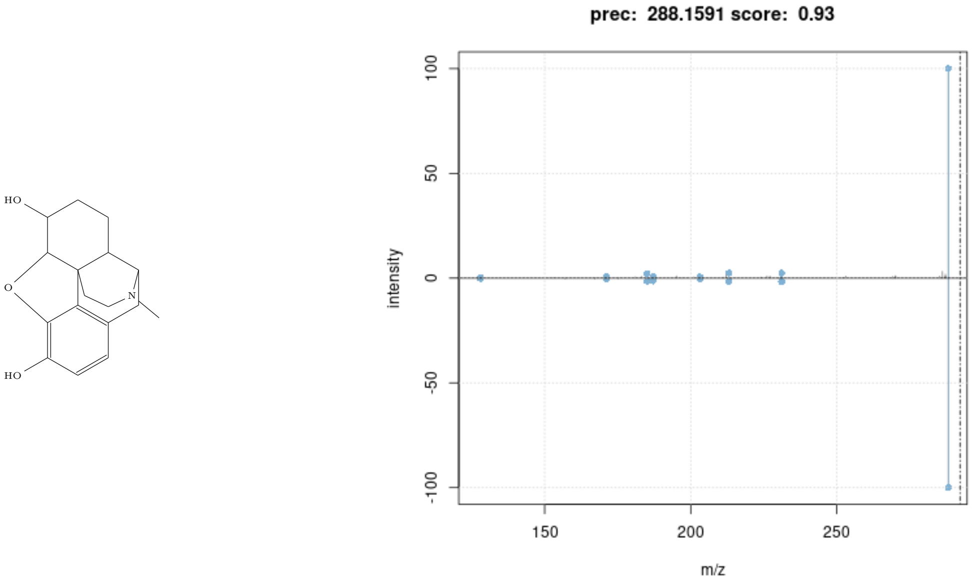

Figure S48: Spectra comparison between in-silico deconjugated spectra and reference spectra for dihydromorphine.

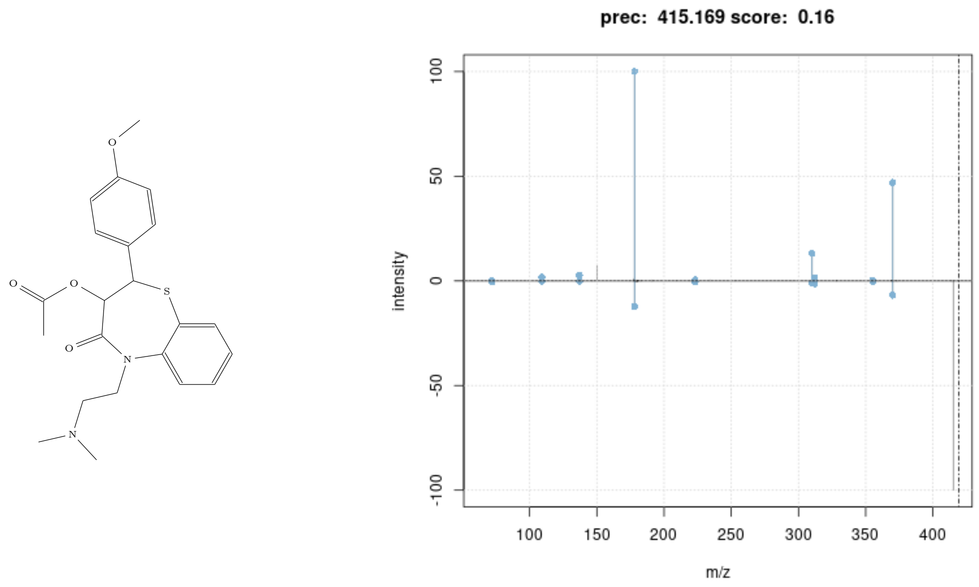

Figure S49: Spectra comparison between in-silico deconjugated spectra and reference spectra for diltiazem.

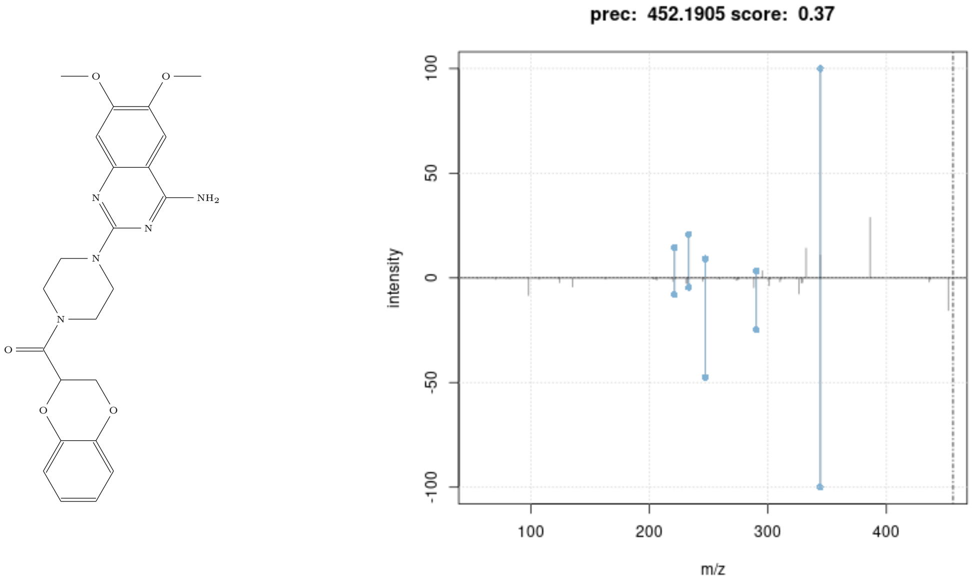

Figure S50: Spectra comparison between in-silico deconjugated spectra and reference spectra for doxazosin.

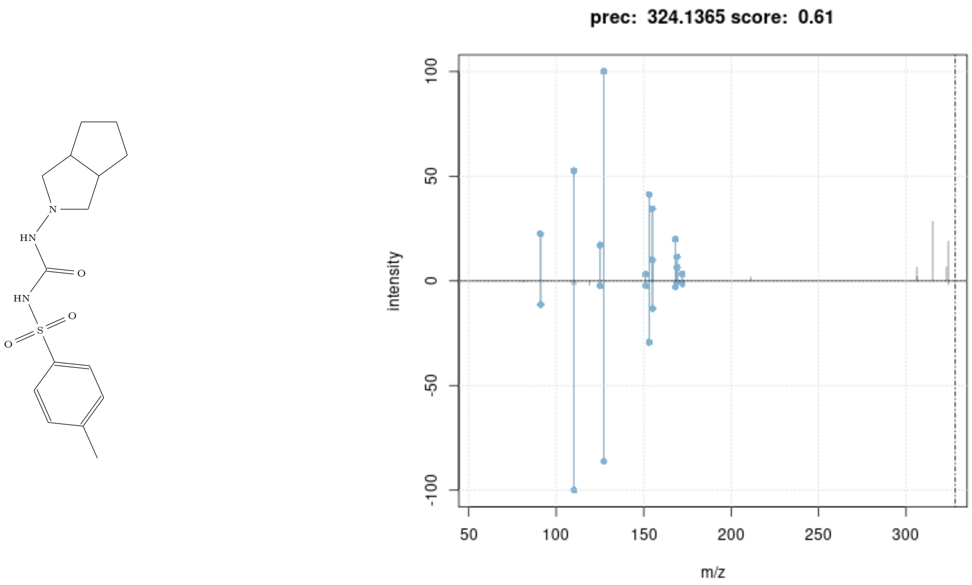

Figure S51: Spectra comparison between in-silico deconjugated spectra and reference spectra for gliclazide.

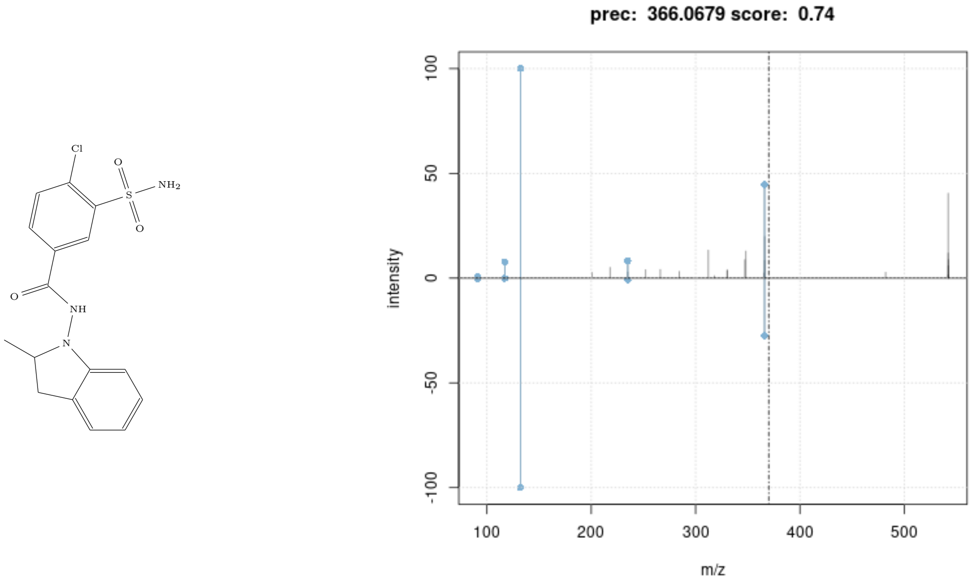

Figure S52: Spectra comparison between in-silico deconjugated spectra and reference spectra for indapamide.

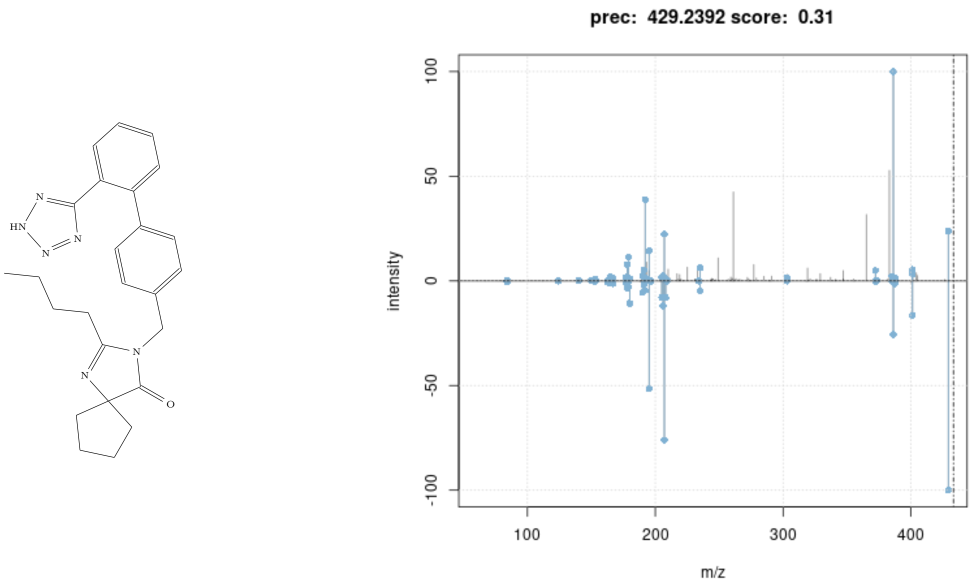

Figure S53: Spectra comparison between in-silico deconjugated spectra and reference spectra for irbesartan.

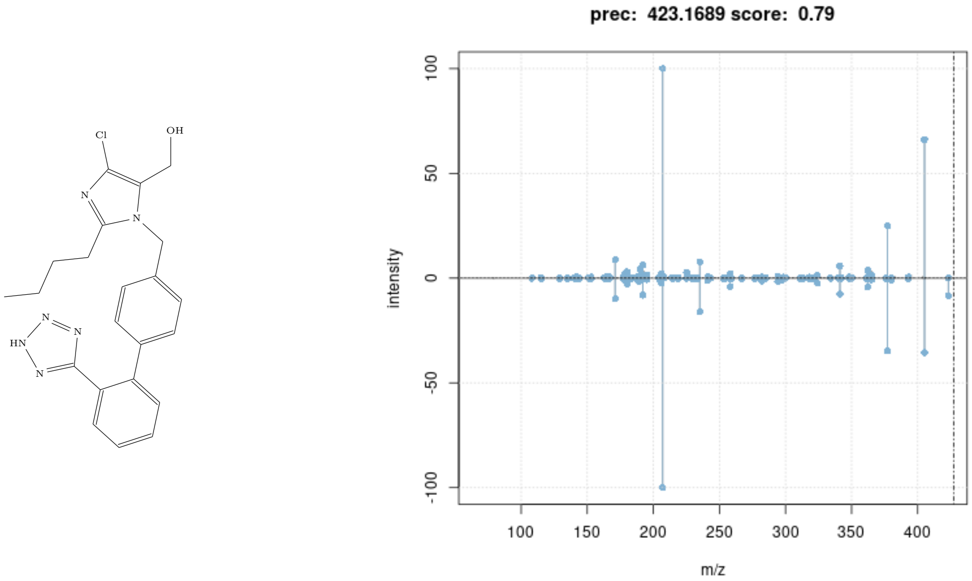

Figure S54: Spectra comparison between in-silico deconjugated spectra and reference spectra for losartan.

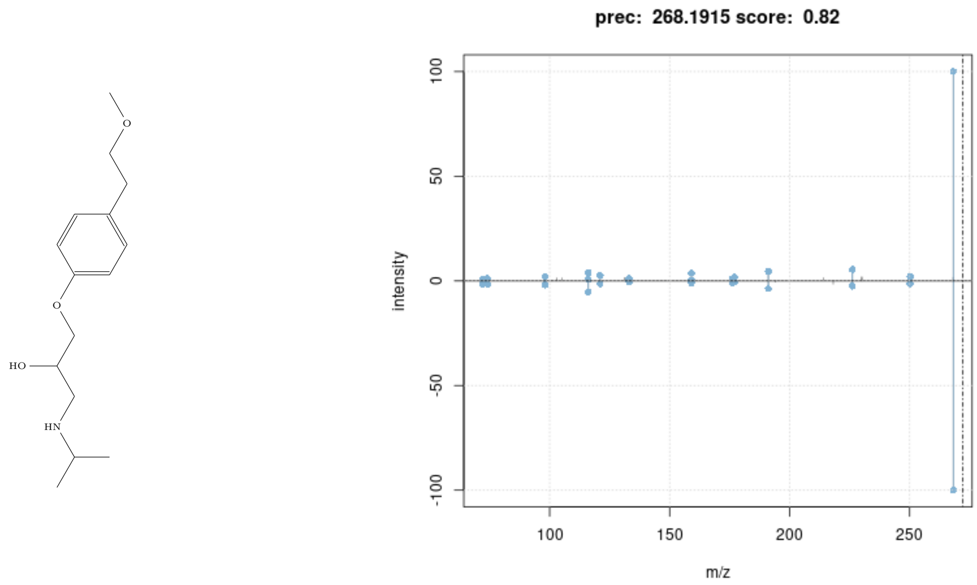

Figure S55: Spectra comparison between in-silico deconjugated spectra and reference spectra for metoprolol.

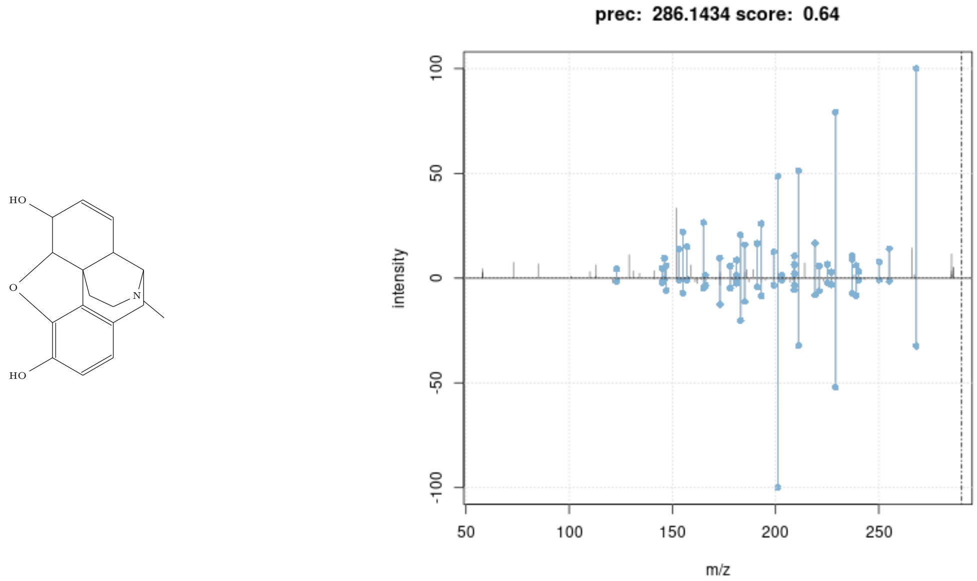

Figure S56: Spectra comparison between in-silico deconjugated spectra and reference spectra for morphine.

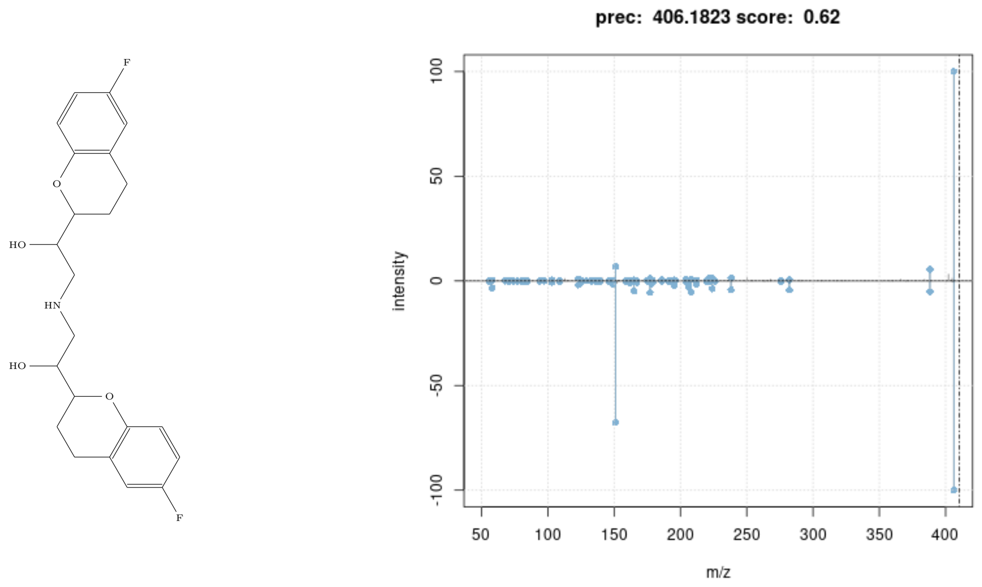

Figure S57: Spectra comparison between in-silico deconjugated spectra and reference spectra for nebivolol.

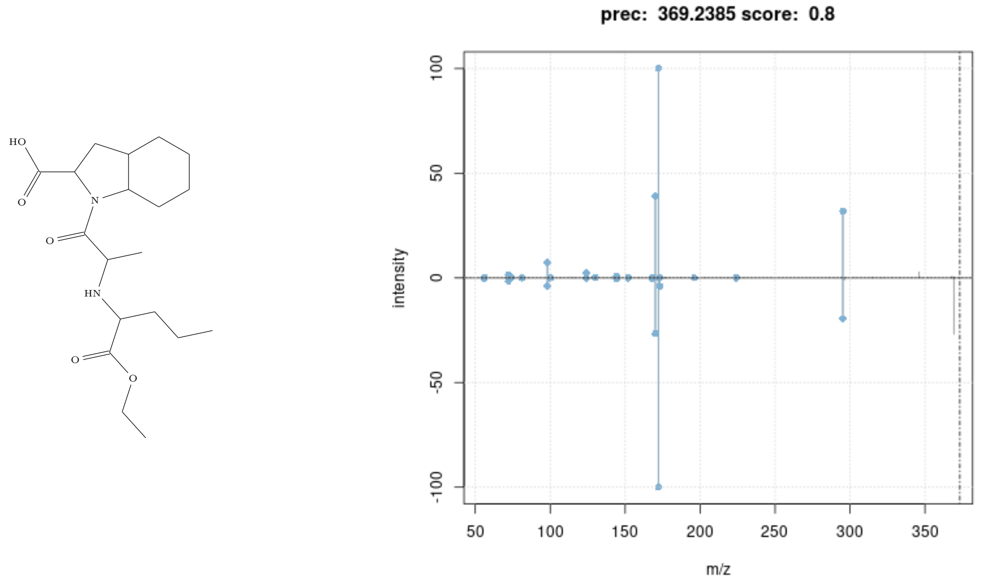

Figure S58: Spectra comparison between in-silico deconjugated spectra and reference spectra for perindopril.

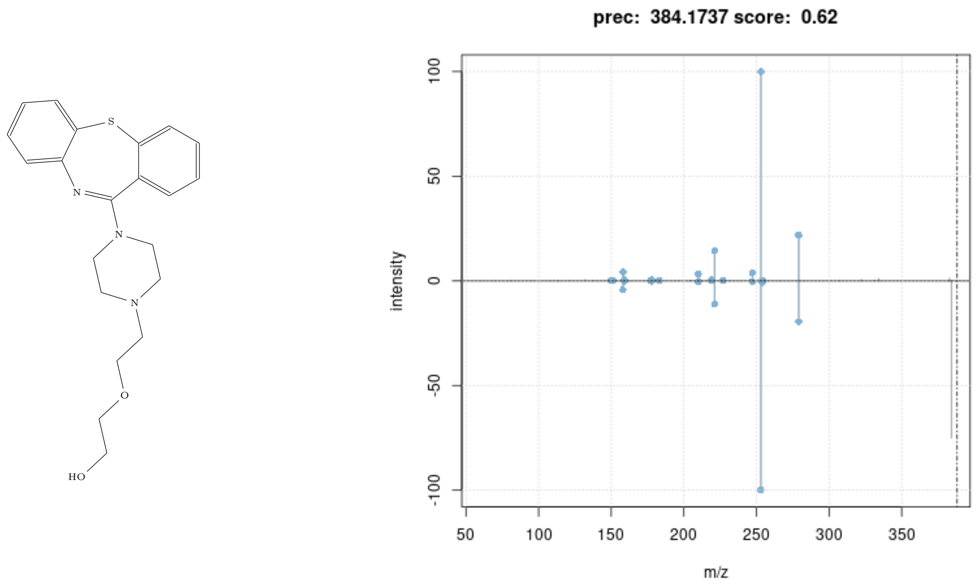

Figure S59: Spectra comparison between in-silico deconjugated spectra and reference spectra for quetiapine.

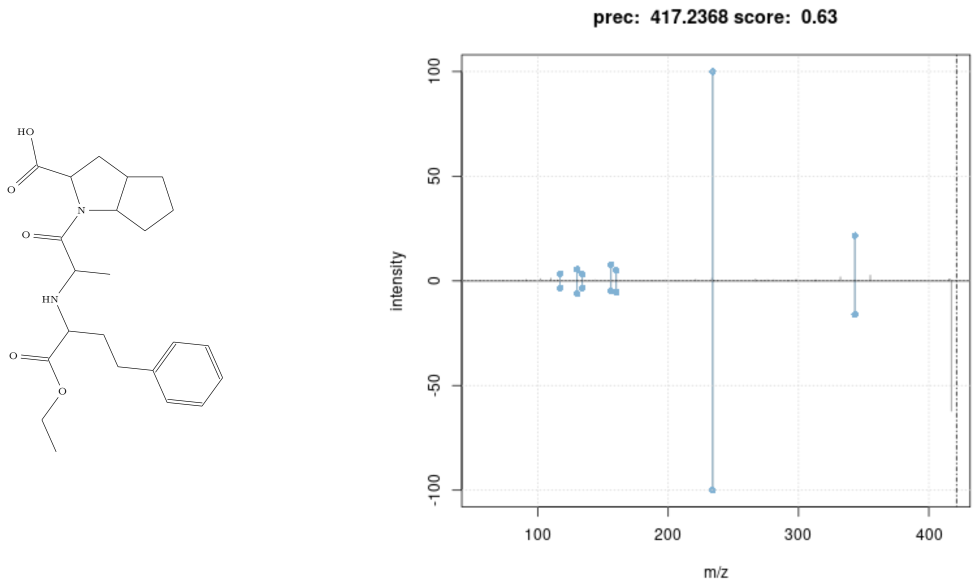

Figure S60: Spectra comparison between in-silico deconjugated spectra and reference spectra for ramipril.

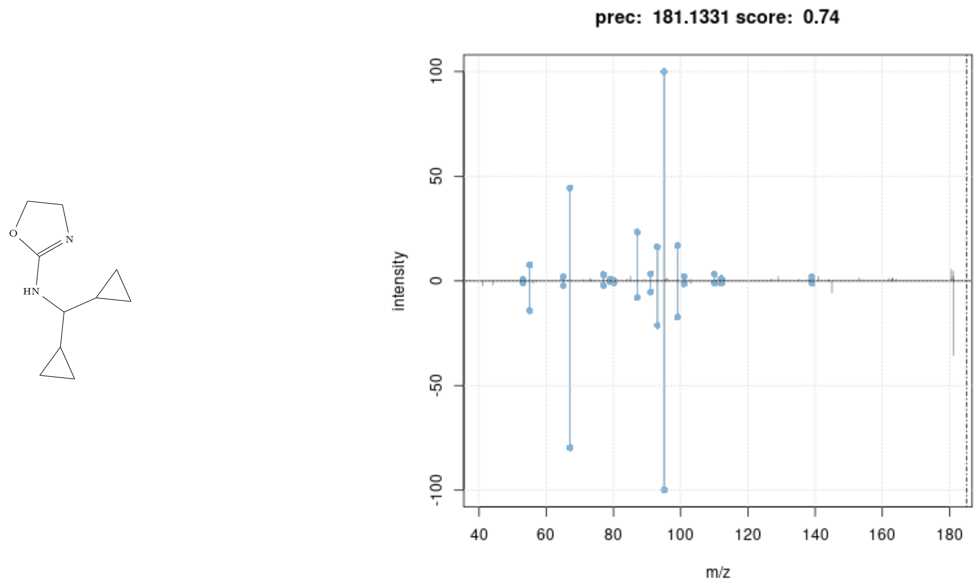

Figure S61: Spectra comparison between in-silico deconjugated spectra and reference spectra for rilmenidine.

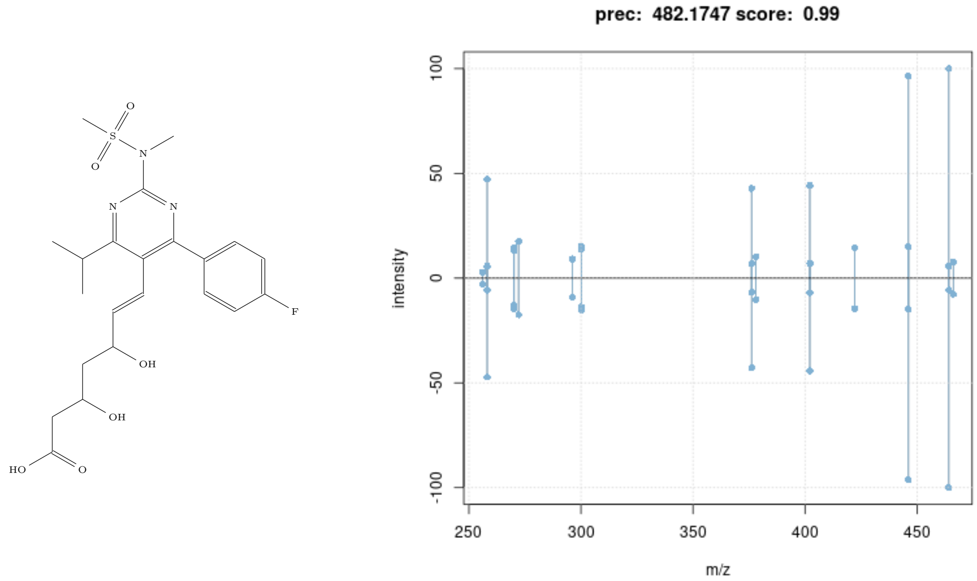

Figure S62: Spectra comparison between in-silico deconjugated spectra and reference spectra for rosuvastatin.

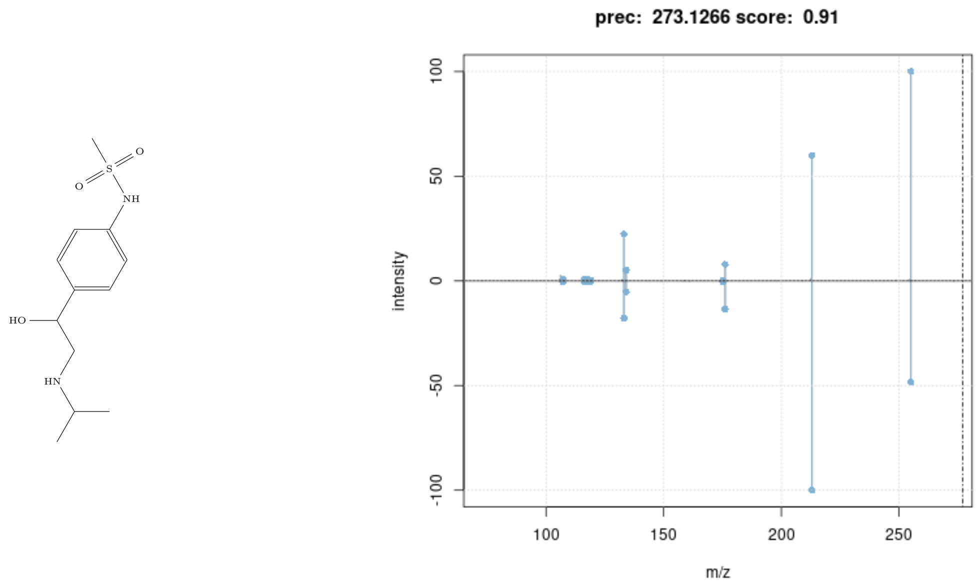

Figure S63: Spectra comparison between in-silico deconjugated spectra and reference spectra for sotalol.

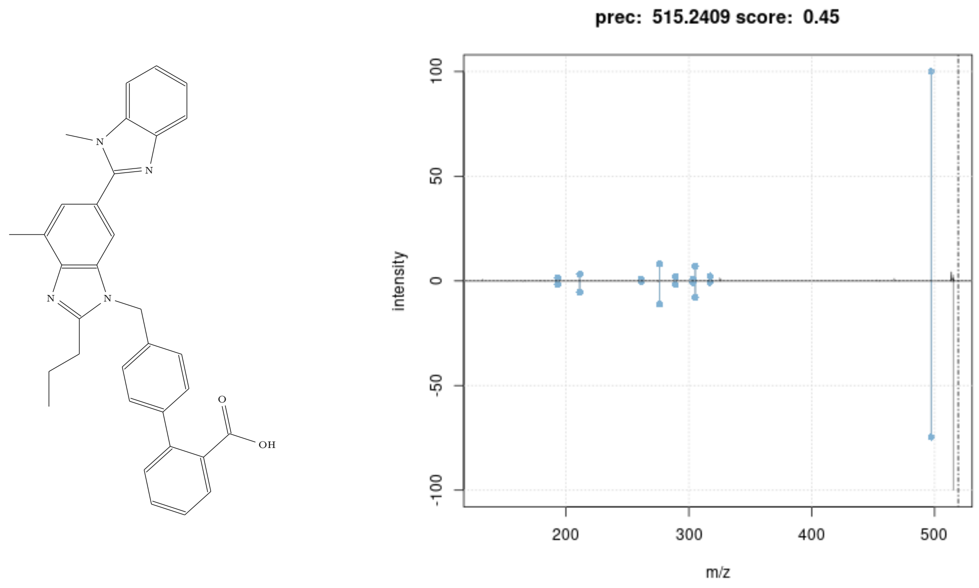

Figure S64: Spectra comparison between in-silico deconjugated spectra and reference spectra for telmisartan.

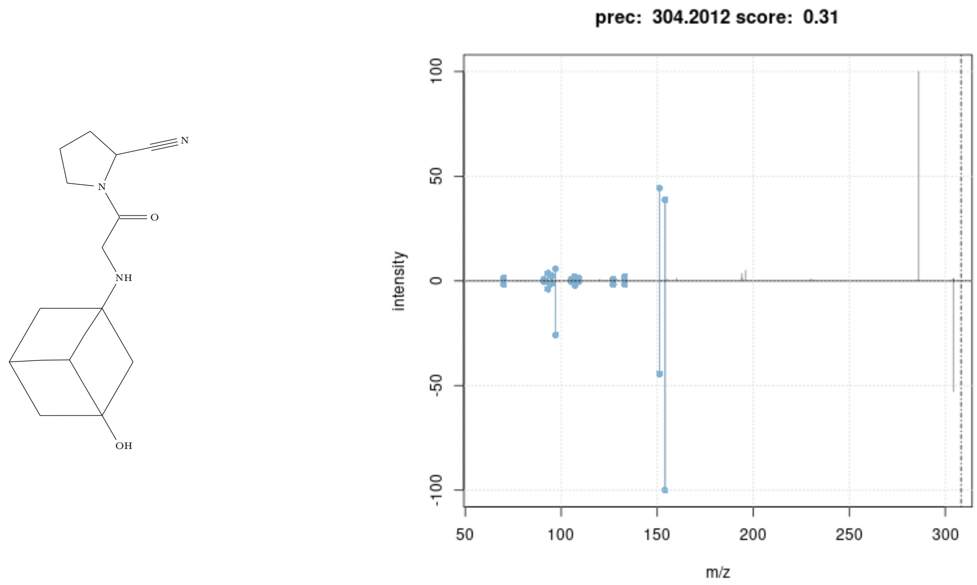

Figure S65: Spectra comparison between in-silico deconjugated spectra and reference spectra for vildagliptine.

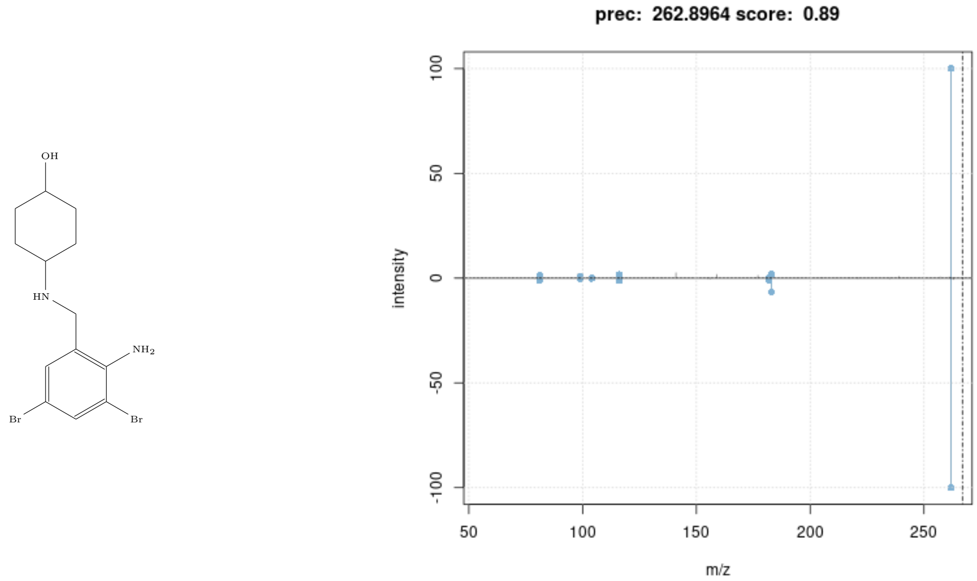

Figure S66: Spectra comparison between in-silico deconjugated spectra and reference spectra for ambroxol.

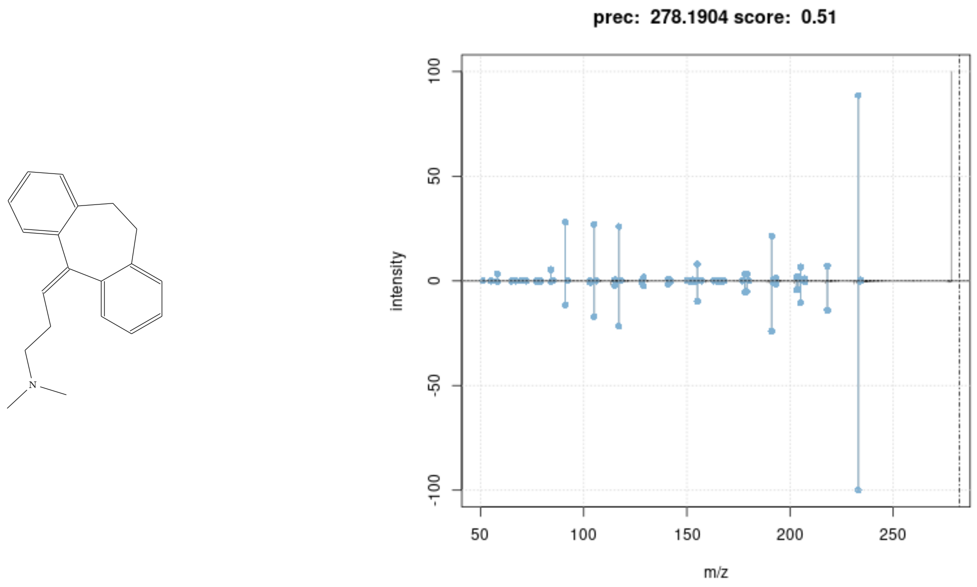

Figure S67: Spectra comparison between in-silico deconjugated spectra and reference spectra for amytriptyline.

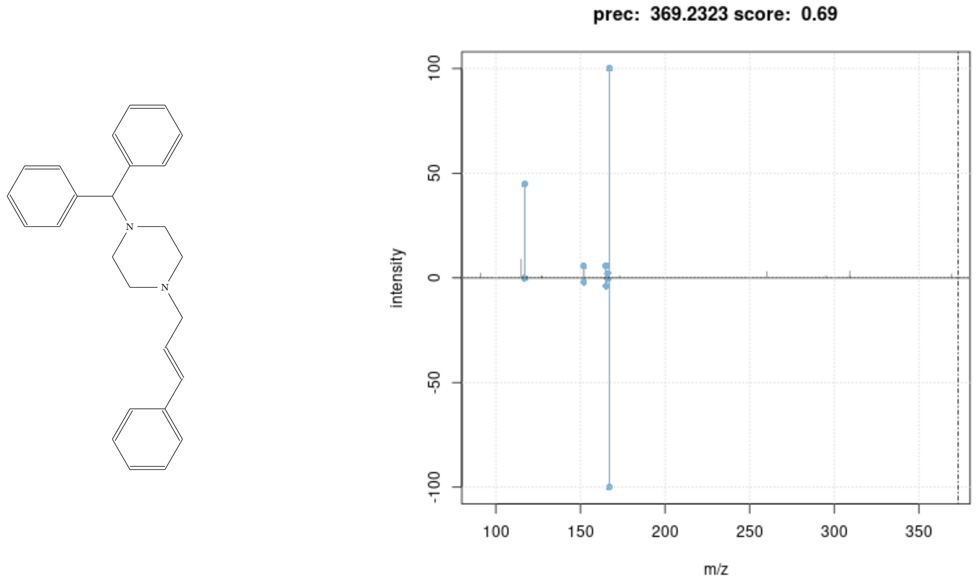

Figure S68: Spectra comparison between in-silico deconjugated spectra and reference spectra for cinnarizine.

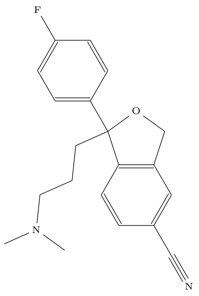

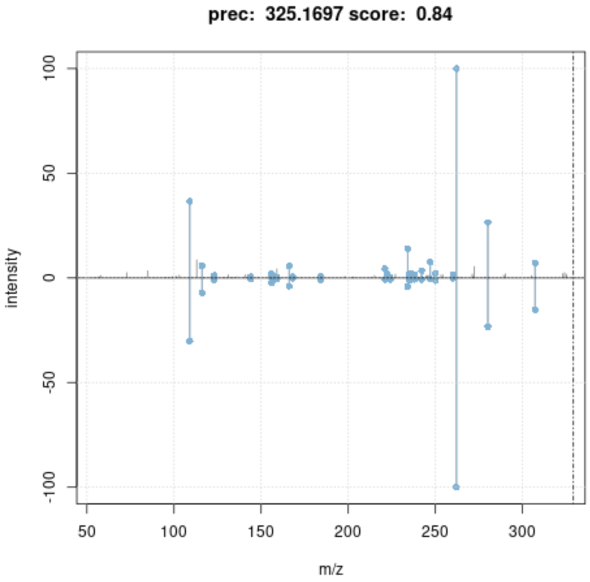

Figure S69: Spectra comparison between in-silico deconjugated spectra and reference spectra for citalopram.

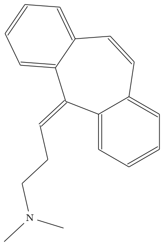

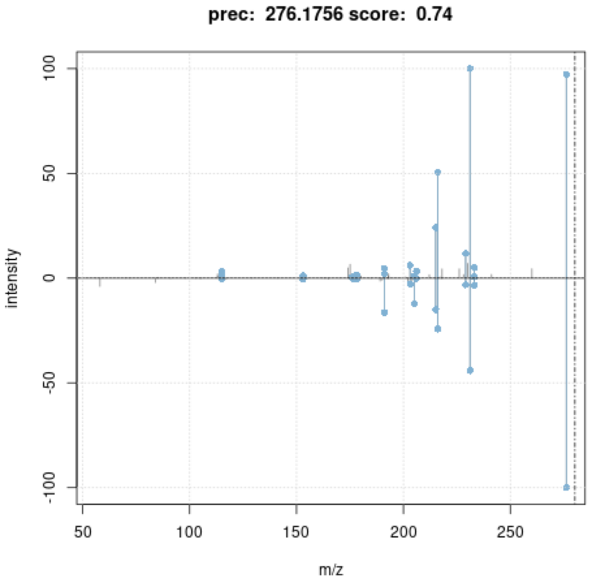

Figure S70: Spectra comparison between in-silico deconjugated spectra and reference spectra for cyclobenzaprine.

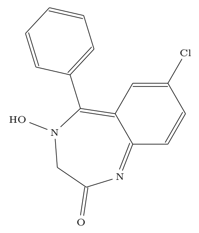

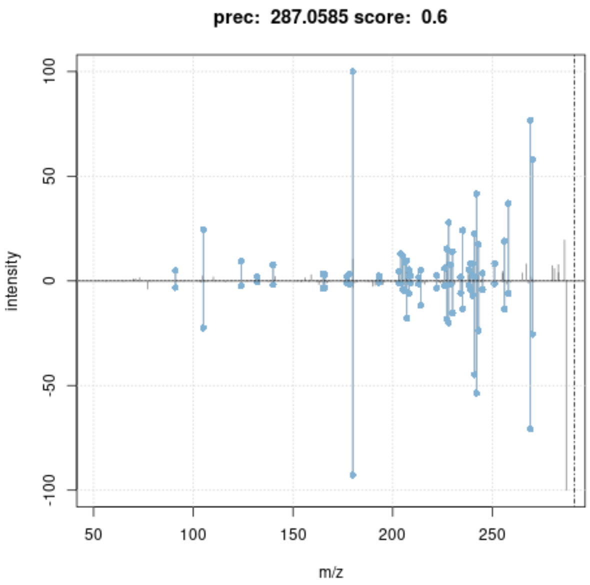

Figure S71: Spectra comparison between in-silico deconjugated spectra and reference spectra for demoxepam.

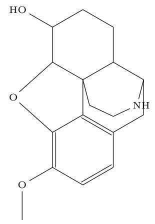

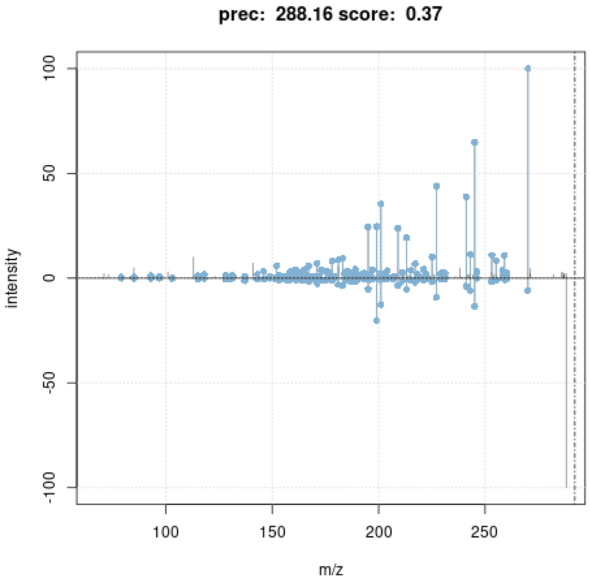

Figure S72: Spectra comparison between in-silico deconjugated spectra and reference spectra for desmethyldihydrocodeine.

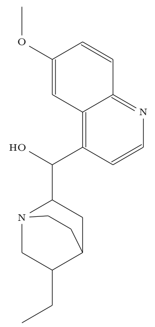

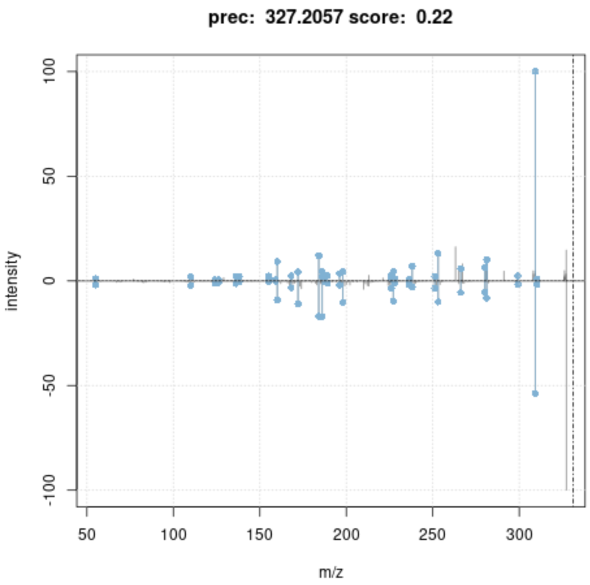

Figure S73: Spectra comparison between in-silico deconjugated spectra and reference spectra for hydroquinine.

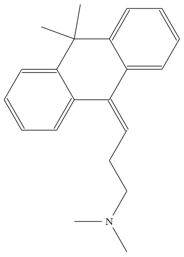

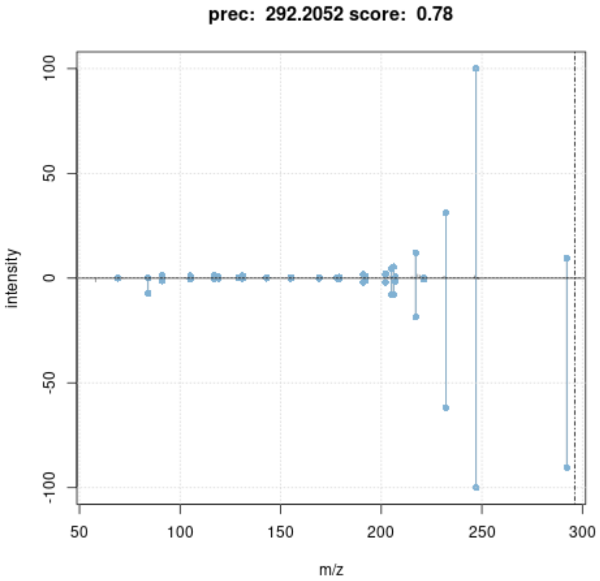

Figure S74: Spectra comparison between in-silico deconjugated spectra and reference spectra for melitracene.

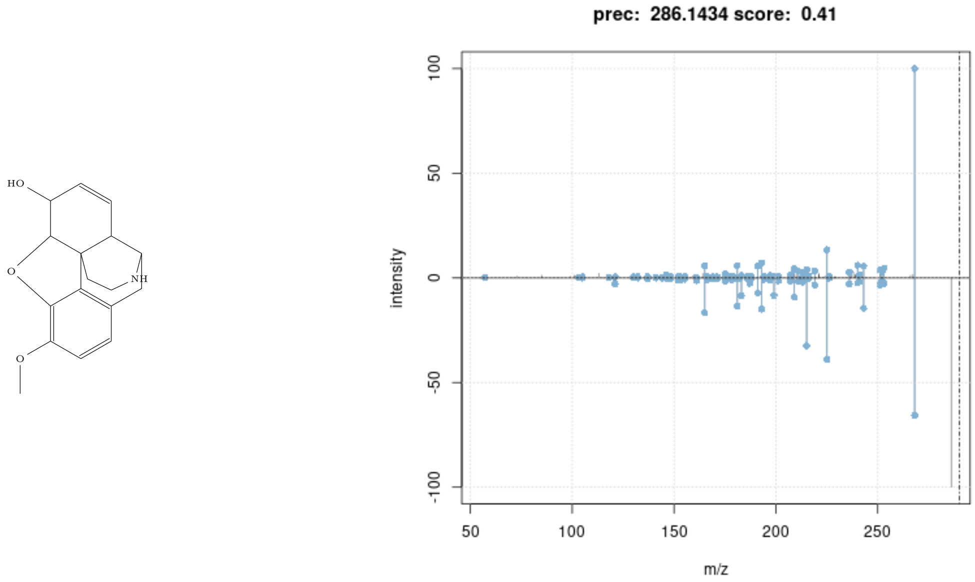

Figure S75: Spectra comparison between in-silico deconjugated spectra and reference spectra for norcodeine.

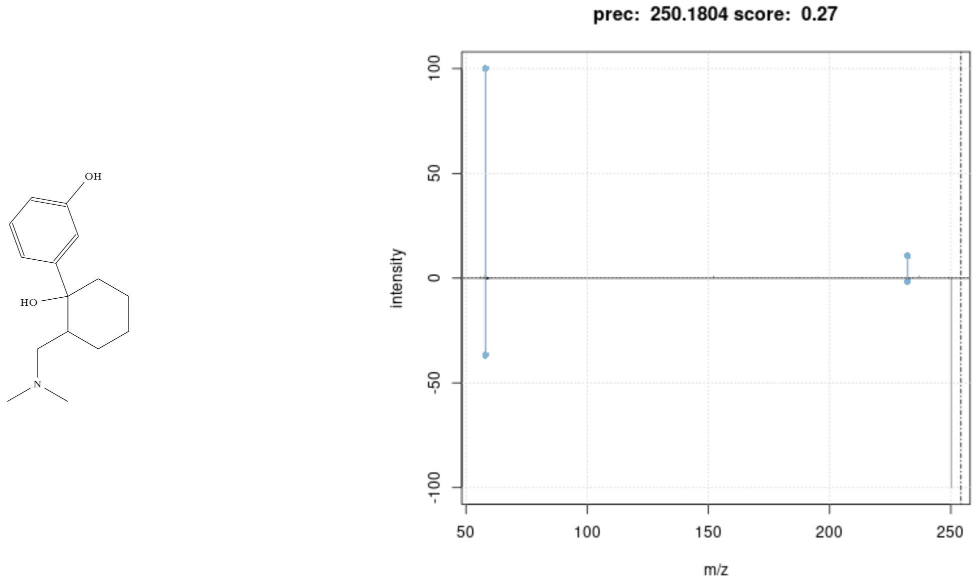

Figure S76: Spectra comparison between in-silico deconjugated spectra and reference spectra for o-desmethyltramadol.

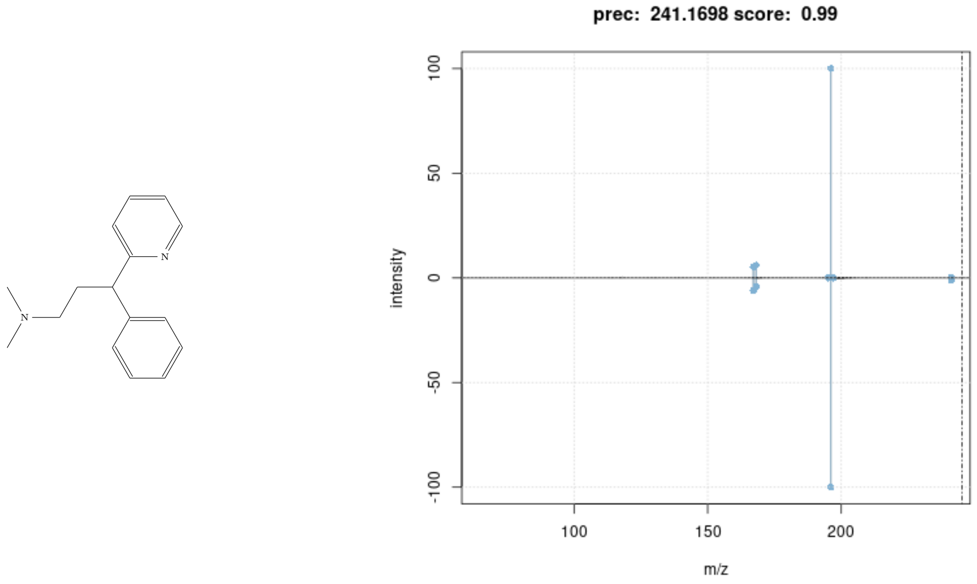

Figure S77: Spectra comparison between in-silico deconjugated spectra and reference spectra for pheniramine.

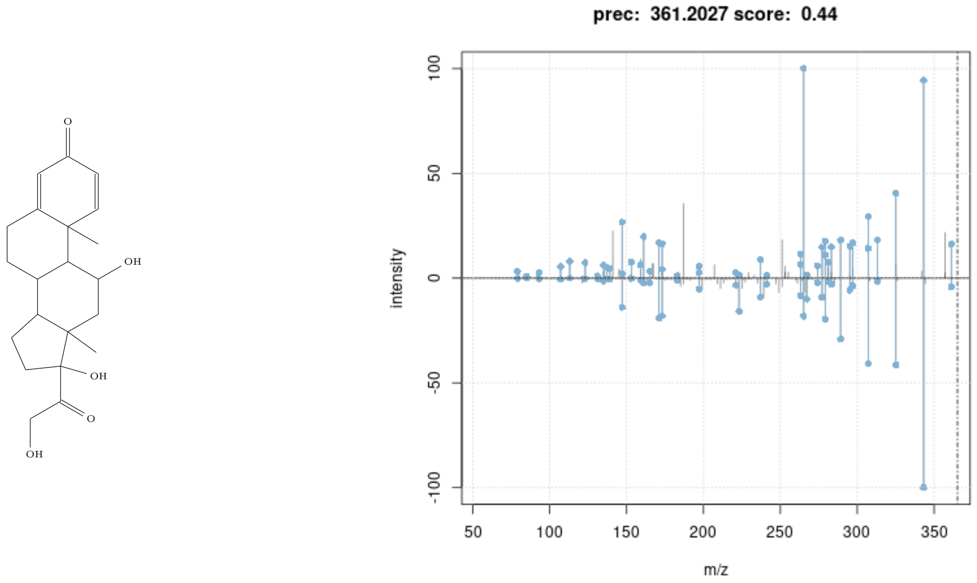

Figure S78: Spectra comparison between in-silico deconjugated spectra and reference spectra for prednisolone.

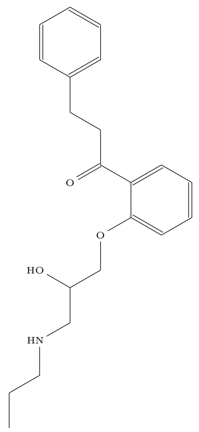

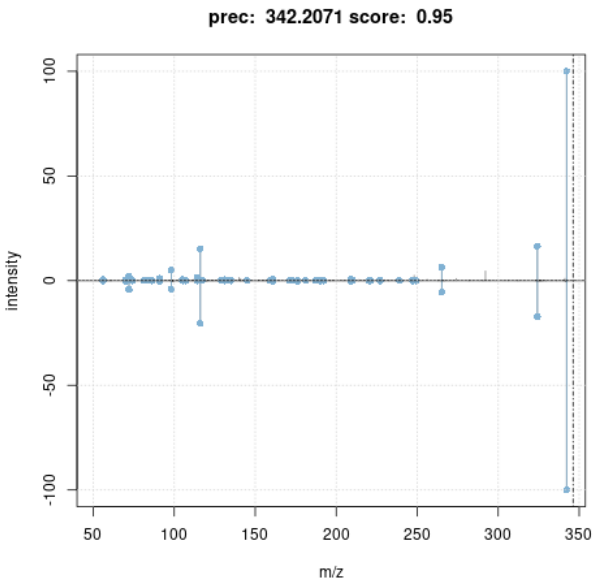

Figure S79: Spectra comparison between in-silico deconjugated spectra and reference spectra for propafenone.

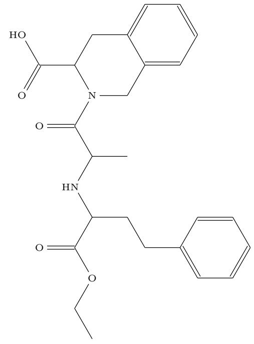

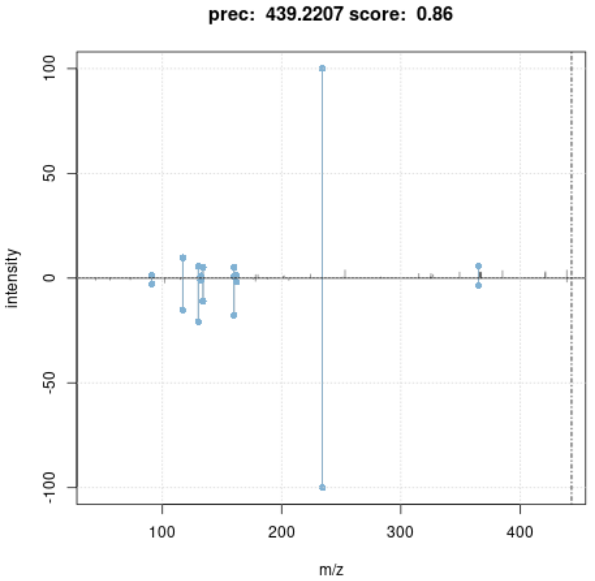

Figure S80: Spectra comparison between in-silico deconjugated spectra and reference spectra for quinapril.

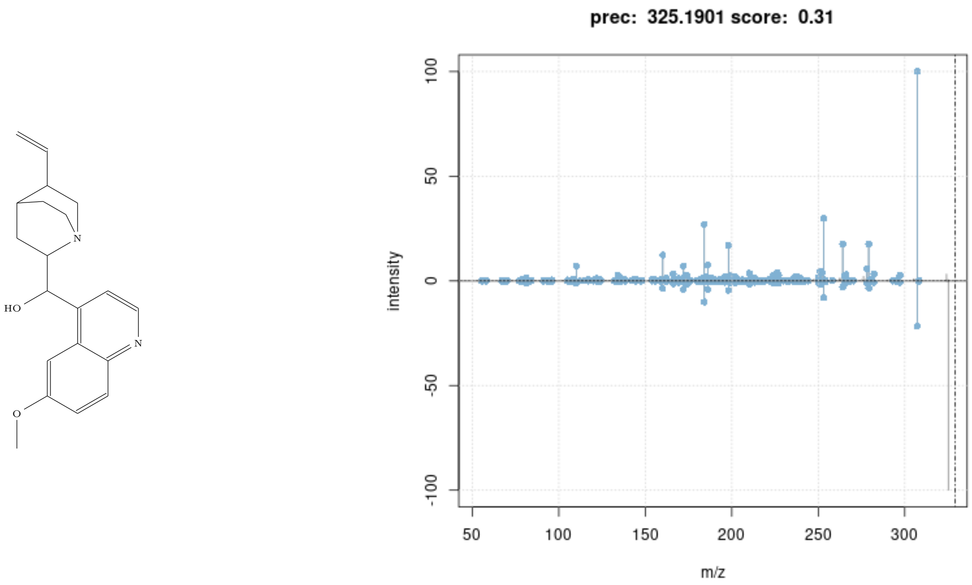

Figure S81: Spectra comparison between in-silico deconjugated spectra and reference spectra for quinidine.

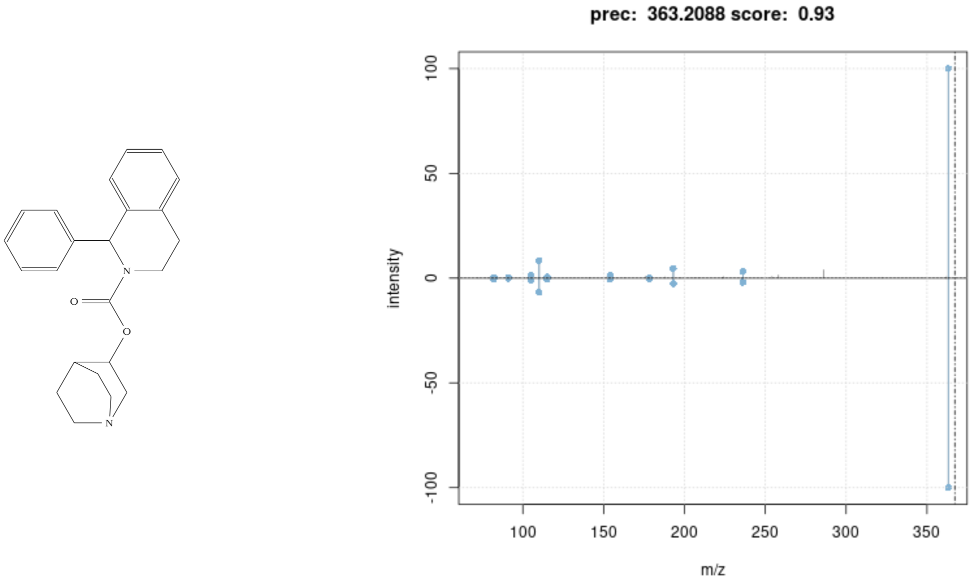

Figure S82: Spectra comparison between in-silico deconjugated spectra and reference spectra for solifenacin.

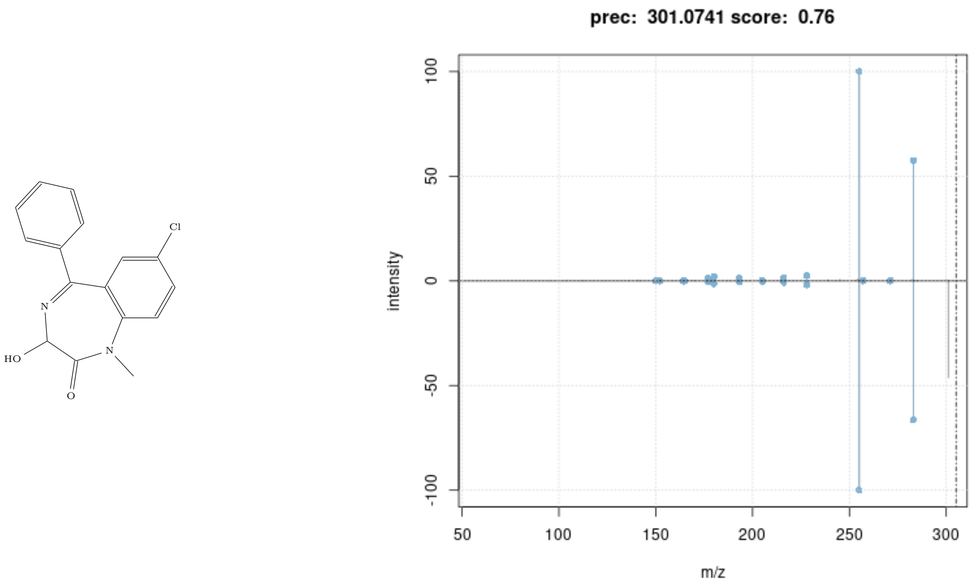

Figure S83: Spectra comparison between in-silico deconjugated spectra and reference spectra for temazepam.

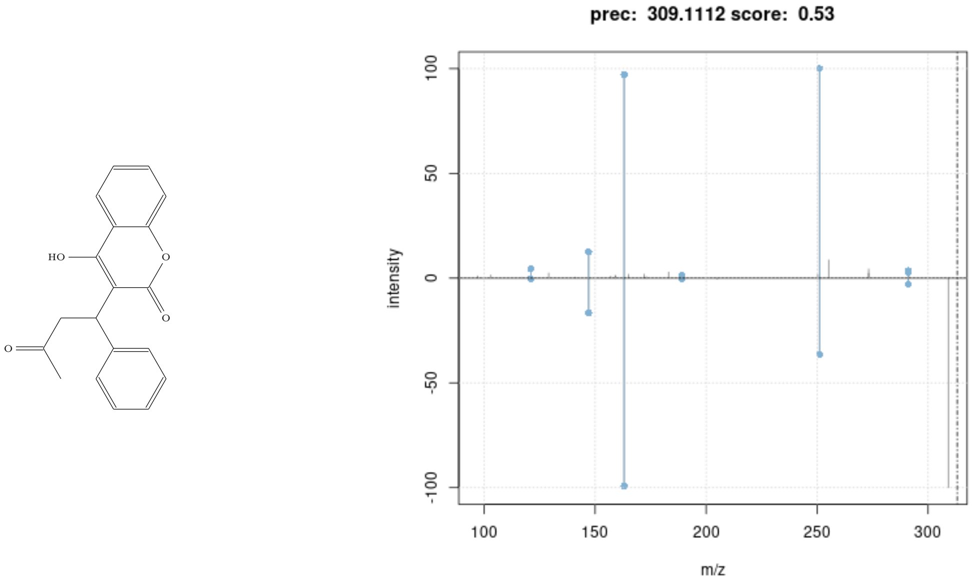

Figure S84: Spectra comparison between in-silico deconjugated spectra and reference spectra for warfarin.

References

(1) Degawa, M.; Shoji, Y.; Masuko, K.; Hashimoto, Y. Mutagenicity of Metabolites of Carcinogenic Aminoazo Dyes. *Cancer Letters* **1979**, *8*, 71–76.

(2) Dahl-Puustinen, M.-L.; Åberg-Wistedt, A.; Bertilsson, L. Glucuronidation of Amitriptyline in Man in Vivo. *Pharmacology & Toxicology* **1989**, *65*, 37–39.

(3) Abshagen, U.; Bablok, W.; Koch, K.; Lang, P. D.; Schmidt, H. A. E.; Senn, M.; Stork, H. Disposition Pharmacokinetics of Bezafibrate in Man. *European Journal of Clinical Pharmacology* **1979**, *16*, 31–38.

(4) Caldwell, G., Yan, Z., Eds. *Optimization in Drug Discovery: In Vitro Methods*, second edition ed.; Methods in Pharmacology and Toxicology; Humana Press: New York, 2014.

(5) Shelnutt, S. R.; Cimino, C. O.; Wiggins, P. A.; Ronis, M. J.; Badger, T. M. Pharmacokinetics of the Glucuronide and Sulfate Conjugates of Genistein and Daidzein in Men and Women after Consumption of a Soy Beverage. *The American Journal of Clinical Nutrition* **2002**, *76*, 588–594.

(6) Breyer-Pfaff, U.; Fischer, D.; Winne, D. Biphasic Kinetics of Quaternary Ammonium Glucuronide Formation from Amitriptyline and Diphenhydramine in Human Liver Microsomes. *Drug Metabolism and Disposition* **1997**, *25*, 340.

(7) Chambers, J. E.; Greim, H.; Kendall, R. J.; Segner, H.; Sharpe, R. M.; Van Der Kraak, G. Human and Ecological Risk Assessment of a Crop Protection Chemical: A Case Study with the Azole Fungicide Epoxiconazole. *Critical Reviews in Toxicology* **2014**, *44*, 176–210.

(8) Friedecký, D.; Mičová, K.; Faber, E.; Hrdá, M.; Široká, J.; Adam, T. Detailed Study of Imatinib Metabolization Using High-Resolution Mass Spectrometry. *Journal of Chromatography A* **2015**, *1409*, 173–181.

(9) Niemeijer, N. R.; Gerding, T. K.; De Zeeuw, R. A. Glucuronidation of Labetalol at the Two Hydroxy Positions by Bovine Liver Microsomes. Isolation, Purification, and Structure Elucidation of the Glucuronides of Labetalol. *Drug Metabolism and Disposition: The Biological Fate of Chemicals* **1991 Jan-Feb**, *19*, 20–23.

(10) Chen, M. In *Handbook of Metabolic Pathways of Xenobiotics*; Lee, P. W., Aizawa, H., Gan, L. L., Prakash, C., Zhong, D., Eds.; John Wiley & Sons, Ltd: Chichester, UK, 2013; pp 1–5.

(11) McGurk, K. A.; Remmel, R. P.; Hosagrahara, V. P.; Tosh, D.; Burchell, B. Reactivity of Mefenamic Acid 1-o-Acyl Glucuronide with Proteins in Vitro and Ex Vivo. *Drug Metabolism and Disposition: The Biological Fate of Chemicals* **1996**, *24*, 842–849.

(12) Vignali, C.; Groppi, A.; Brandolini, F.; Avato, F. M.; Talarico, A.; Gaudio, R. M.; Morini, L. Mirtazapine Fatal Poisoning. *Forensic Science International* **2017**, *276*, e8–e12.

(13) Cardoso, J. d. O.; Oliveira, R. V.; Lu, J. B. L.; Desta, Z. In Vitro Metabolism of Montelukast by Cytochrome P450s and UDP-Glucuronosyltransferases. *Drug Metabolism and Disposition* **2015**, *43*, 1905–1916.

(14) Sisenwine, S. F.; Tio, C. O.; Hadley, F. V.; Liu, A. L.; Kimmel, H. B.; Ruelius, H. W. Species-Related Differences in the Stereoselective Glucuronidation of Oxazepam. *Drug Metabolism and Disposition* **1982**, *10*, 605.

(15) Shen, Z.; Reed, J. R.; Creighton, M.; Liu, D. Q.; Tang, Y. S.; Hora, D. F.; Feeney, W.; Szewczyk, J.; Bakhtiar, R.; Franklin, R. B.; Vincent, S. H. Identification of Novel Metabolites of Pioglitazone in Rat and Dog. *Xenobiotica* **2003**, *33*, 499–509.

(16) Midha, K. K.; Roscoe, R. M. H.; Wilson, T. W.; Cooper, J. K.; Loo, J. C. K.; Ho-Ngoc, A.; McGilveray, I. J. Pharmacokinetics of Glucuronidation of Propranolol Fol-

lowing Oral Administration in Humans. *Biopharmaceutics & Drug Disposition* **1983**, *4*, 331–338.

(17) Joint Meeting of the FAO Panel of Experts on Pesticide Residues in Food and the Environment and the WHO Core Assessment Group on Pesticide Residues, . . R., Italy; World Health Organization,; Food and Agriculture Organization of the United Nations,; FAO Panel of Experts on Pesticide Residues in Food and the Environment,; WHO Expert Group on Pesticide Residues, *Pesticide Residues in Food - 2008: Toxicological Evaluations : Joint Meeting of the FAO Panel of Experts on Pesticide Residues in Food and the Environment and the WHO Core Assessment Group on Pesticide Residues, Rome, Italy, 9-18 September 2008*; World Health Organization: Geneva, Switzerland, 2006.

(18) Sun, D.; Jones, N. R.; Manni, A.; Lazarus, P. Characterization of Raloxifene Glucuronidation: Potential Role of UGT1A8 Genotype on Raloxifene Metabolism *In Vivo*. *Cancer Prevention Research* **2013**, *6*, 719–730.

(19) Kaku, T.; Ogura, K.; Nishiyama, T.; Ohnuma, T.; Muro, K.; Hiratsuka, A. Quaternary Ammonium-Linked Glucuronidation of Tamoxifen by Human Liver Microsomes and UDP-Glucuronosyltransferase 1A4. *Biochemical Pharmacology* **2004**, *67*, 2093–2102.

(20) Mercadante, R.; Polledri, E.; Scurati, S.; Moretto, A.; Fustinoni, S. Identification and Quantification of Metabolites of the Fungicide Tebuconazole in Human Urine. *Chemical Research in Toxicology* **2014**, *27*, 1943–1949.

(21) Ebner, T.; Heinzel, G.; Prox, A.; Beschke, K.; Wachsmuth, H. Disposition and Chemical Stability of Telmisartan 1-<em>O</Em>-Acylglucuronide. *Drug Metabolism and Disposition* **1999**, *27*, 1143.

(22) Rxlist.Com. 2017. RxList - The Internet Drug Index for Prescription Drugs, Medications and Pill Identifier. [Online].

(23) Zumoff, B.; Rosenfeld, R. S.; Friedman, M.; Byers, S. O.; Rosenman, R. H.; Hellman, L. Elevated Daytime Urinary Excretion of Testosterone Glucuronide in Men with the Type A Behavior Pattern:. *Psychosomatic Medicine* **1984**, *46*, 223–225.

(24) Kuuranne, T.; Kurkela, M.; Thevis, M.; Schänzer, W.; Finel, M.; Kostiainen, R. GLUCURONIDATION OF ANABOLIC ANDROGENIC STEROIDS BY RECOMBINANT HUMAN UDP-GLUCURONOSYLTRANSFERASES. *Drug Metabolism and Disposition* **2003**, *31*, 1117.

(25) Jauch, R.; Bozler, G.; Hammer, R.; Koss, F. W.; Karlsson, M.; Vitek, E.; Häring, I.; Beschke, K.; Hadamovsky, S.; Maass, D.; Wollmann, R. [Ambroxol, studies of biotransformation in man and determination in biological samples (author's transl)]. *Arzneimittel-Forschung* **1978**, *28*, 904–911.

(26) El-Sayed, G. O.; Yasin, S. A.; El Badawy, A. A. Voltammetric Behavior and Determination of Cinnarizine in Pharmaceutical Formulations and Serum. *Analytical Letters* **2008**, *41*, 3021–3033.

(27) Dalgaard, L.; Larsen, C. Metabolism and Excretion of Citalopram in Man: Identification of O-Acyl- and N-Glucuronides. *Xenobiotica* **1999**, *29*, 1033–1041.

(28) Dinis-Oliveira, R. J. Metabolic Profile of Oxazepam and Related Benzodiazepines: Clinical and Forensic Aspects. *Drug Metabolism Reviews* **2017**, *49*, 451–463.

(29) Yue, Q.; Svensson, J.; Alm, C.; Sjoqvist, F.; Sawe, J. Interindividual and Interethnic Differences in the Demethylation and Glucuronidation of Codeine. *British Journal of Clinical Pharmacology* **1989**, *28*, 629–637.

(30) Glöckl, I.; Blaschke, G.; Veit, M. Validated Methods for Direct Determination of Hydroquinone Glucuronide and Sulfate in Human Urine after Oral Intake of Bearberry Leaf Extract by Capillary Zone Electrophoresis. *Journal of Chromatography B: Biomedical Sciences and Applications* **2001**, *761*, 261–266.

(31) Innocenti, F.; Yodergraber, A.; Ramirez, J.; Ratain, M. In Vitro Glucuronidation of Prednisone. *Clinical Pharmacology & Therapeutics* **2005**, *77*, P36–P36.

(32) Luo, H.; Hawes, E. M.; McKay, G.; Korchinski, E. D.; Midha, K. K. Glucuronidation of Aliphatic Tertiary Amines, a General Phenomenon in the Metabolism of H-Antihistamines in Humans. *Xenobiotica* **1991**, *21*, 1281–1288.

(33) Xie, S.; Zeng, S. Stereoselective Glucuronidation of Propafenone and Its Analogues by Human Recombinant UGT1A9. *Chemical and Pharmaceutical Bulletin* **2010**, *58*, 879–883.

(34) Zhang, H.; Coville, P. F.; Walker, R. J.; Miners, J. O.; Birkett, D. J.; Wanwimolruk, S. Evidence for Involvement of Human CYP3A in the 3-hydroxylation of Quinine. *British Journal of Clinical Pharmacology* **1997**, *43*, 245–252.

(35) Lehtonen, P.; Sten, T.; Aitio, O.; Kurkela, M.; Vuorensola, K.; Finel, M.; Kostiainen, R. Glucuronidation of Racemic O-Desmethyltramadol, the Active Metabolite of Tramadol. *European Journal of Pharmaceutical Sciences* **2010**, *41*, 523–530.

(36) Jones, D. R.; Moran, J. H.; Miller, G. P. Warfarin and UDP-Glucuronosyltransferases: Writing a New Chapter of Metabolism. *Drug Metabolism Reviews* **2010**, *42*, 55–61.

(37) Semenistaya, E. N.; Savel'eva, N. B.; Sobolevskii, T. G.; Rodchenkov, G. M. Direct Determination of Beta-Blockers and Their Metabolites in Urine by Liquid Chromatography Coupled with Tandem Mass Spectrometry. *Journal of Analytical Chemistry* **2013**, *68*, 173–182.

(38) Chen, M. In *Handbook of Metabolic Pathways of Xenobiotics*; Lee, P. W., Aizawa, H., Gan, L. L., Prakash, C., Zhong, D., Eds.; John Wiley & Sons, Ltd: Chichester, UK, 2013; pp 1–3.

(39) Sugawara, Y.; Nakamura, S.; Usuki, S.; Ito, Y.; Suzuki, T.; Ohashi, M.; Harigaya, S. Metabolism of Diltiazem. II. Metabolic Profile in Rat, Dog and Man. *Journal of Pharmacobio-Dynamics* **1988**, *11*, 224–233.

(40) Oida, T.; Yoshida, K.; Kagemoto, A.; Sekine, Y.; Higashijima, T. The Metabolism of Gliclazide in Man. *Xenobiotica* **1985**, *15*, 87–96.

(41) Caruso, F. S.; Szabadi, R. R.; Vukovich, R. A. Pharmacokinetics and Clinical Pharmacology of Indapamide. *American Heart Journal* **1983**, *106*, 212–220.

(42) Brocker, C. N.; Velenosi, T.; Flaten, H. K.; McWilliams, G.; McDaniel, K.; Shelton, S. K.; Saben, J.; Krausz, K. W.; Gonzalez, F. J.; Monte, A. A. Metabolomic Profiling of Metoprolol Hypertension Treatment Reveals Altered Gut Microbiota-Derived Urinary Metabolites. *Human Genomics* **2020**, *14*, 10.

(43) McTaggart, F. Comparative Pharmacology of Rosuvastatin. *Atherosclerosis Supplements* **2003**, *4*, 9–14.

(44) Fredenhagen, A.; Kühnöl, J.; Kittelmann, M.; Oberer, L. Gas-Phase Rearrangement of the O -Glucuronide of Vildagliptin Forms Product-Ion Fragments Suggesting Wrongly an N -Glucuronide. *Drug Metabolism and Disposition* **2019**, *47*, 189–193.

(45) Dynorm 2,5 - Fachinformation. https://imedikament.de/dynorm-2-5/fachinformation?doc=1.

(46) Reeves, P. R.; McAinsh, J.; McIntosh, D. A. D.; Winrow, M. J. Metabolism of Atenolol in Man. *Xenobiotica* **1978**, *8*, 313–320.

(47) Vieira, C. P.; Neves, D. V.; Cesarino, E. J.; Rocha, A.; Poirier, S.; Lanchote, V. L. An Indirect Stereoselective Analysis of Nebivolol Glucuronides in Plasma by LC–MS/MS: Application to Clinical Pharmacokinetics. *Journal of Pharmaceutical and Biomedical Analysis* **2017**, *144*, 25–30.

(48) Kirkwood, L. C.; Nation, R. L.; Somogyi*, A. A. GLUCURONIDATION OF DIHYDROCODEINE BY HUMAN LIVER MICROSOMES AND THE EFFECT OF INHIBITORS. *Clinical and Experimental Pharmacology and Physiology* **1998**, *25*, 266–270.

(49) Zheng, M.; McErlane, K. M.; Ong, M. C. Hydromorphone Metabolites: Isolation and Identification from Pooled Urine Samples of a Cancer Patient. *Xenobiotica* **2002**, *32*, 427–439.
